
JOURNAL INFORMATION
==============================
NLM Title Abbreviation: Can J Pain
Journal ID: Can J Pain
NLM Journal ID: 101720589

Canadian Journal of Pain

EISSN: 2474-0527
Publisher: Taylor & Francis

ARTICLE INFORMATION
==============================
PMCID: PMC8210881
DOI: 10.1080/24740527.2021.1914233
Article ID: 1914233
Article version: 1
Article version: Version of Record
Subjects: Abstract, Research Poster Abstracts

Research Poster Abstracts

Research Poster Abstracts

Canadian Journal of Pain

Electronic publication date: 2021 Apr 22
Publication date: 2021
Volume: 5
Issue: 2
First page: A48
Last page: A137
Copyright: © 2021 The Author(s). Published with license by Taylor & Francis Group, LLC.
Copyright year: 2021
Copyright holder: The Author(s)
License: This is an Open Access article distributed under the terms of the Creative Commons Attribution License (http://creativecommons.org/licenses/by/4.0/), which permits unrestricted use, distribution, and reproduction in any medium, provided the original work is properly cited.
License URL: https://creativecommons.org/licenses/by/4.0/

Figure count: 0
Page count: 90

==============================
CANADIAN JOURNAL OF PAIN
2021, VOL. 5, NO. 2, A48–A137
https://doi.org/10.1080/24740527.2021.1914233

Taylor & Francis
Taylor & Francis Group

OPEN ACCESS Check for updates

### The effects of a physical training intervention on pain, disability and psychological factors in patients with chronic low back pain

Erika Gentile[a], Florian Bobeuf[b], Anna Bendas[a], Anaïs Lépine Lopez[a], Benjamin Pageaux[c], Louis Bherer[d], Julien Cohen-Adad[b], Pierre Rainville[b], and Mathieu Roy[e]

[a]McGill University, Department of Psychology, Montreal, Quebec, Canada; [b]Centre de Recherche de l'Institut Universitaire de Gériatrie de Montréal, Montreal, Quebec, Canada; [c]Université de Montréal, Department of Kinesiology & Centre de Recherche de l'Institut Universitaire de Gériatrie de Montréal, Montreal, Quebec, Canada; [d]Centre de Recherche de & l'Institut Universitaire de Gériatrie de Montréal l'Institut de Cardiologie de Montréal, Montreal, Quebec, Canada; [e]McGill University, Department of Psychology & Centre de recherche de l'Institut universitaire de gériatrie de Montréal, Montreal, Quebec, Canada

Physical training has shown benefits in pain management among patients with chronic low back pain (**CLBP**), as it has improved pain severity, disability, quality of life, and physical and psychological functioning. However, there is limited empirical evidence examining how training is having such effects. The present study aimed to test the impact of a physical training intervention on back pain intensity, perceived disability, and psychological variables and to examine potential mediating factors. Fifty patients (33 female, 17 male, age range 21-74 years) with CLBP participated in the study and were either tested before and after the training program (training group) or after a similar period without training (wait list control group). Measurements include self-reported back pain intensity, perceived disability, and psychological variables (i.e., pain catastrophizing, kinesiophobia, depression, and anxiety). Concordant with other studies, patients in the training group reported lower back pain intensity [F(1, 46)=21.247, p=.000], perceived disability [F (1, 47)=11.761, p=.001], pain catastrophizing [F(1, 46) =4.560, p=.038], kinesiophobia [F(1, 47)=5.309, p=.026], and depressive symptoms [F(1, 42)=5.053, p=.030]. Further, changes in back pain intensity significantly mediated the effects of group on perceived disability (b= -2.4978, SE = 1.314, 95% CI= -5.4526, -.3264) while changes in pain catastrophizing and kinesiophobia partially mediated this effect. These results demonstrate the therapeutic effects of our training intervention in patients with CLBP and provide insight into some of the mechanisms mediating these changes.

**Introduction/AimChronic**: low back pain (**CLBP**) is a major cause of disability globally and in Canada. Current treatments either lack efficacy, have dose-limiting side-effects or involve the use of addictive substances. Fortunately, physical exercise is a welcome exception. Indeed, according to a recent systematic review conducted by the American College of Physicians, physical exercise therapy is one of the few interventions reliably showing positive effects in the management of chronic low back pain. More specifically, it has been shown to improve pain severity, disability, overall quality of life, and physical and psychological functioning. Therefore, it has been strongly recommended as a first-line treatment for CLBP. However, there is limited empirical evidence examining the underlying mechanisms of such therapeutic effects, which hampers the development of effective chronic pain treatment. The present study aimed to test the impact of a physical exercise intervention on pain, perceived disability, and psychological variables and to examine potential mediating factors.

**Methods**: Fifty patients (33 female, 17 male, age range 21-74 years) with CLBP participated in the study. Among these patients, a subset ($n_{training}$=34) underwent a physical training program comprising of three, one-hour weekly sessions of combined moderate-to-high intensity aerobic and resistance training. They were assessed immediately before and after training on self-reported back pain intensity, perceived disability, and psychological variables (i.e., pain catastrophizing, kinesiophobia, depression, and anxiety). The others ($n_{waitlist}$=16) underwent baseline and post-training assessments on the same measures but completed the intervention at a later date.

**Results**: The findings of the present study demonstrate post-training effects on pain [F(1, 46)=21.247, p=.000], perceived disability [F(1, 47)=11.761, p=.001], and psychological variables – pain catastrophizing [F(1, 46)=4.560, p=.038)], kinesiophobia [F(1, 47)=5.309, p=.026], and depression [F(1, 42)=5.053, p=.030], while controlling for gender. Simple mediation analyses revealed that changes in low back pain intensity significantly mediated the effect of group on perceived disability (b= -2.4978, SE = 1.314, 95% CI= -5.4526, -.3264) and that changes in self-reported pain catastrophizing and kinesiophobia partially mediated this effect.

**Discussion/Conclusions**: The present study provides converging evidence that a non-pharmacological approach to pain management may be beneficial in reducing back pain intensity and disability as well as improve psychological variables among patients suffering from

CLBP. An understanding of the underlying mechanisms implicated in the therapeutic effects of training will aid in identifying and modifying the characteristics of the training program to achieve maximal effectiveness for each patient. Further, since current effective treatment options for pain management remain scarce, results from this study can aid in the development of novel non-pharmacological treatments that would harness the same mechanisms as our physical training program.

### ORCID

Florian Bobeuf http://orcid.org/0000-0002-4798-1091
Anna Bendas http://orcid.org/0000-0002-2903-4015
Mathieu Roy http://orcid.org/0000-0002-3335-445X

### Conflict of Interest

None.

### Sources of Financial Support

This project is funded by the Quebec Bio-Imaging Network: Pilot Grant, and the Canadian Institutes of Health Research: Project Grant.

© 2021 The Author(s). Published with license by Taylor & Francis Group, LLC.
This is an Open Access article distributed under the terms of the Creative Commons Attribution License (http://creativecommons.org/licenses/by/4.0/), which permits unrestricted use, distribution, and reproduction in any medium, provided the original work is properly cited.

### The Chronic Pain Network's (CPN) Clinical Research Network (CRN)

Ian Gilron[a], Chris DeBow[b], Norm Buckley[c], Jennifer Anthonypillai[d], Krista Baerg[e], Amel Bagdhadli[f], Jillian Banfield[g], Etienne Bisson[h], Stephanie Blackman[i], Fiona Campbell[j], Jessie Dhillon[k], Scott Duggan[h], Renee El-Gabalawy[k], Allen Finley[l], Lauren Harris[i], Keith Jarvi[m], Madison Kennedy[n], Susan Lau[o], Gabrielle Logan[k], Mary Lynch[p], Aaron MacInnes[j], Casey McMahon[e], Melanie Noel[q], Gabrielle Page[f], Anne Marie Pinard[r], Patricia Poulin[s], Saifee Rashiq[t], Nivez Rasic, Danielle Rice, Tiffany Rice, Rachel Roy[k], Yoram Shir[x], Jennifer Stinson[i], Sylvie Toupin[x], Elodie Traverse[r], Michelle Verrier[t], Marc White[y], and Ramesh Zacharias[z]

[a]Queen's University, Biomedical and Molecular Science, Kingston, Ontario, Canada; [b]Chronic Pain Network, Patient Partner, Halifax, Nova Scotia, Canada; [c]McMaster University, Anesthesiology, Hamilton, Ontario, Canada; [d]Hamilton Health Sciences, Department of Anesthesiology, Hamilton, Ontario, Canada; [e]University of Saskatchewan, Department of Pediatrics, Saskatoon, Saskatchewan, Canada; [f]Université de Montréal, Anesthesiology and Pain Medicine/Research Center of the Centre hospitalier de l'Université de Montréal, Montreal, Quebec, Canada; [g]Dalhousie University, Pain & Perioperative Medicine, Halifax, Nova Scotia, Canada; [h]Queen's University, Anesthesiology and Perioperative Medicine, Kingston, Ontario, Canada; [i]The Hospital for Sick Children, Anesthesia and Pain Medicine, Toronto, Ontario, Canada; [j]University of British Columbia, Department of Anesthesiology, Vancouver, British Columbia, Canada; [k]University of Manitoba, Anesthesia and Perioperative Medicine, Winnipeg, Manitoba, Canada; [l]Dalhousie University, Department of Anesthesia, Halifax, Nova Scotia, Canada; [m]Mount Sinai Hospital, Department of Surgery, University of Toronto, Ontario, Canada; [n]Alberta Children's Hospital, Department of Anesthesia, Calgary, Alberta, Canada; [o]Mount Sinai Hospital, Division of Urology, Toronto, Ontario, Canada; [p]Dalhousie University, Department of Anesthesia, Pain Management and Perioperative Medicine, Psychiatry and Pharmacology, Halifax, Nova Scotia, Canada; [q]University of Calgary, Department of Psychology, Calgary, Alberta, Canada; [r]département d'anesthésiologie et de soins intensifs, Université Laval; [s]University of Ottawa, Anesthesiology and Pain Medicine, Ottawa, Ontario, Canada; [t]University of Alberta, Department of Anesthesiology & Pain Medicine, Edmonton, Alberta, Canada; [u]Alberta Children's Hospital, Department of Anesthesiology, Calgary, Alberta, Canada; [v]The Ottawa Hospital, Department of Anesthesiology and Pain Medicine, Ottawa, Ontario, Canada; [w]University of Calgary, Department of Anesthesiology, Perioperative and Pain Medicine, Alberta Children's Hospital, Calgary, Alberta, Canada; [x]McGill University, The Alan Edwards Pain Management Unit, Montreal, Quebec, Canada; [y]Work Wellness and Disability Prevention Institute, CEO and President, Vancouver, British Columbia, Canada; [z]McMaster University, Department of Anesthesia, Hamilton, Ontario, Canada

**Introduction/Aim**: The CRN produces real-world pain research within the CPN. Patients referred to CRN sites: A) may be excellent research participants, and B) could ultimately benefit from advances of such research. A shared infrastructure could facilitate cost-effective multicenter research. This emerging network will provide synergies that combine: 1) skilled academic clinicians who identify new testable hypotheses, 2) experienced researchers who provide research methodology expertise, 3) efficient high-quality clinics that enhance study participant recruitment.

**Methods**: We have established a sustainable funding model for the CRN. The Vision is for a network of real-world clinics dedicated to the research of the Canadian Chronic Pain Network. The path to excellence is to ensure that every network member: 1) has something valuable to offer, 2) has a vested interest in overall network success, 3) interacts effectively with the rest of the network.

**Results**: Site investigators and coordinators have been identified for 14 sites (9 adult; 3 pediatric; 2 pediatric-adult). To date, 7 multicenter studies involving 2 or more CRN sites are already underway with others being planned – including a Canada-wide, national pain registry. Furthermore, over 100 local site studies have begun since the establishment of the CRN and most of these new studies would not have taken place without the CRN.

**Discussion/Conclusions**: To date, this CRN is the largest national clinical pain research network in Canada and we are in the process of establishing a sustainability model that will allow this network to expand and evolve for years to come.

### ORCID

Krista Baerg http://orcid.org/0000-0001-5192-7285
Fiona Campbell http://orcid.org/0000-0001-6482-0743
Allen Finley http://orcid.org/0000-0003-4579-7749
Melanie Noel http://orcid.org/0000-0003-3752-8055
Jennifer Stinson http://orcid.org/0000-0002-9969-8052

**CONTACT** Ian Gilron gilroni@queensu.ca

© 2021 The Author(s). Published with license by Taylor & Francis Group, LLC.
This is an Open Access article distributed under the terms of the Creative Commons Attribution License (http://creativecommons.org/licenses/by/4.0/), which permits unrestricted use, distribution, and reproduction in any medium, provided the original work is properly cited.

### Examining pain symptoms in persistent genital arousal disorder/genitopelvic dysesthesia (PGAD/GPD) with the fear avoidance model

Robyn A. Jackowich[a] and Caroline F. Pukall[a]

[a]Queen's University, Kingston, Ontario, Canada

**Introduction/Aim**: PGAD/GPD is a distressing, but poorly understood condition characterized by persistent, unwanted sensations of genital arousal in the absence of psychological desire. Up to 44% of those with PGAD/GPD report painful symptoms of genital arousal, however, very little is known about the experience of pain in individuals with this condition. The present study examined pain characteristics in PGAD/GPD and, using the framework of the Fear Avoidance Model, investigated associations among PGAD/GPD symptom distress and cognitive/emotional variables.

**Methods**: 345 individuals (312 women, 33 men) with current symptoms of PGAD/GPD completed an online survey of PGAD/GPD symptoms, symptom catastrophizing (PCS), hypervigilance (PVAQ), and symptom anxiety (PASS-20).

**Results**: 81% ($n$ = 253/313) of participants described their symptoms as painful (mean = 4.41; 0: no pain to 10: extremely painful). Associated pain symptoms/painful activities included: painful orgasms (43%, $n$ = 134/315), pain with sexual activity (58%; $n$ = 174/298), and painful urination (53%, $n$ = 170/318). 30% ($n$ = 103/345) reported experiencing other forms of chronic pain. Significant associations were found among PGAD/GPD symptom distress and 1) symptom catastrophizing ($r$ =.605), 2) hypervigilance to symptoms ($r$ = .318), and 3) symptom anxiety ($r$ = .400; all $p$'s <.001).

**Discussion/Conclusion**: PGAD/GPD is a distressing condition that is often described as painful and frequently associated with other pain conditions. In line with current consensus in the field, PGAD/GPD is likely best conceptualized as a 'dysesthesia' (unwanted, unpleasant sensation). Future research and interventions may benefit from being guided by a chronic pain framework.

### ORCID

Caroline F. Pukall http://orcid.org/0000-0003-1553-6118

© 2021 The Author(s). Published with license by Taylor & Francis Group, LLC.
This is an Open Access article distributed under the terms of the Creative Commons Attribution License (http://creativecommons.org/licenses/by/4.0/), which permits unrestricted use, distribution, and reproduction in any medium, provided the original work is properly cited.

### Music-induced analgesia: Comparing musicotherapy, emotional dimensions of music, and preferred music

Anaïs Lépine Lopez[a], Darius Valevicius[a], Ajar Diushekeeva[b], and Mathieu Roy[a]

[a]McGill University, Psychology, Montreal, Quebec, Canada; [b]Université de Montréal, Psychologie, Montreal, Quebec, Canada

**Introduction/Aim**: In recent years, many researchers have been trying to find alternatives to pain medication, and music has been shown to induce analgesia. However, few studies have attempted to identify which type of musical approach are the most predictive of analgesia. In this study, we compared three types of approaches: 1) gradual entrainment towards relaxation as implemented in Music Care (https://www.music-care.com/en/index.html), 2) pre-defined optimal emotional dimensions based on a previous study, and 3) self-selected preferred music.

**Methods:** Participants (n = 65) were asked to listen to different musical excerpts while receiving painful thermal stimulations. They listened to their two favourite songs, a musical excerpt from the musicotherapy program called MusicCare, and songs that represent the best and the worst combinations of emotional dimensions for analgesia, developed in our lab.

**Discussion/Conclusions:** Results show show that there was a significant reduction of pain when participants listened to their favourite songs compared to silence. However, we found no significant pain reduction for the other conditions. Moreover, these results are predicted by chills for pain intensity, chills and valence for pain unpleasantness. These interesting findings suggest that rewarding preferences in music are crucial for pain analgesia and should be considered in clinical settings and future research.

### ORCID

Mathieu Roy http://orcid.org/0000-0002-3335-445X

© 2021 The Author(s). Published with license by Taylor & Francis Group, LLC.
This is an Open Access article distributed under the terms of the Creative Commons Attribution License (http://creativecommons.org/licenses/by/4.0/), which permits unrestricted use, distribution, and reproduction in any medium, provided the original work is properly cited.

### Accessing evidence-based practices for children's pain management: differences in stakeholders' needs and barriers

Nicole E. MacKenzie[a], Christine T. Chambers[b], Kathryn Birnie[c], Jennifer Parker[d], Samina Ali[e], Fiona Campbell[f], Bonnie Stevens[g], G. Allen Finley[h], Jennifer Stinson[i], Doug Maynard[j], Dawn Richards[k], Isabel Jordan[l], Erin Aubrey[m], Chad Larabie[n], Justina Marianayagam[o], Joanne Parker[p], Paula Robeson[q], Elise Reiter[r], and Aimee White[s]

[a]Dalhousie University, Psychology and Neuroscience, Halifax, Nova Scotia, Canada; [b]Dalhousie University, Psychology and Neuroscience, Halifax, Nova Scotia, Canada; [c]University of Calgary, Anesthesiology, Calgary, Alberta, Canada; [d]IWK Health Centre, Centre for Pediatric Pain Research, Halifax, Nova Scotia, Canada; [e]University of Alberta, Pediatrics, Faculty of Medicine & Dentistry, Edmonton, Alberta, Canada; [f]University of Toronto, Anesthesia, Toronto, Ontario, Canada; [g]University of Toronto, Nursing, Toronto, Ontario, Canada; [h]Dalhousie University, Anesthesia, Halifax, Nova Scotia, Canada; [i]Hospital for Sick Children and University of Toronto, Lawrence S. Bloomberg Faculty of Nursing, Toronto, Ontario, Canada; [j]Children's Healthcare Canada, Ottawa, Ontario, Canada; [k]Patient and Family Partner, Toronto, Ontario, Canada; [l]Patient and Family Partner, Squamish, British Columbia, Canada; [m]Solutions for Kids in Pain, Halifax, Nova Scotia, Canada; [n]Solutions for Kids in Pain, Toronto, Ontario, Canada; [o]Northern Ontario School of Medicine, Thunder Bay, Ontario, Canada; [p]Solutions for Kids in Pain, Halifax, Nova Scotia, Canada; [q]Solutions for Kids in Pain, Ottawa, Ontario, Canada; [r]Solutions for Kids in Pain, University of Alberta, Edmonton, Alberta, Canada; [s]Solutions for Kids in Pain, Halifax, Nova Scotia, Canada

**Introduction/Aim**: Knowledge mobilization (KMb) focuses on improving uptake of evidence-based practices (EBPs) through engagement with healthcare professionals (HCPs), researchers, and patients/caregivers. EBP implementation is influenced by an organization's inner-setting (e.g., resources) and outer-setting (e.g., connectedness), as per the Consolidated Framework for Implementation Research (CFIR). The aim was to assess differences in needs and barriers by stakeholder type, within inner- and outer-settings.

**Methods**: Participants completed an online needs assessment regarding stakeholder needs and barriers to EBPs for children's pain management via Solutions for Kids in Pain (SKIP), a national KMb network. The survey consisted of closed-ended questions regarding frequency and type of needs and barriers.

**Results**: 630 stakeholders self-identified as HCPs (62.9%, $n$=396), patients/caregivers (26.0%, $n$=164), and researchers (11.1%, $n$=70). A one-way ANOVA indicated no significant group differences between the frequency of barriers, $F(2)=0.65$, $p=0.52$, or the primary barrier type, identified by stakeholders as knowledge ($\chi^2(2)=0.05$, $p=0.98$). However, EBP accessibility differed significantly, $F(2)=4.60$, $p=0.01$, with researchers reporting poorer accessibility compared to HCPs ($MD=0.63$, $p=0.04$). Chi-square tests indicated significant differences in stakeholder needs to access EBPs, with researchers wanting centralized resource access (52.9%, $n$=37, $\chi^2(2)=5.80$, $p=0.05$), and HCPs and patients/caregivers preferring tools targeting their respective stakeholder groups (HCPs: 61.4%, $n$=243, $\chi^2(2)=36.65$, $p<0.001$; patients/caregivers: 53%, $n$=87, $\chi^2(2)=34.70$, $p<0.001$).

**Discussion/Conclusions**: While stakeholders experience similar outer-setting barriers to accessing EBPs to manage children's pain (e.g., poor accessibility), their access needs differ within the inner-setting (e.g., stakeholder-specific tools). These differences must be considered when developing interventions supporting EBP implementation for children's pain management.

### ORCID

Nicole E. MacKenzie http://orcid.org/0000-0002-7097-5706
Christine T. Chambers http://orcid.org/0000-0002-7138-916X
Kathryn Birnie http://orcid.org/0000-0002-8223-8834
Samina Ali http://orcid.org/0000-0002-0595-364X
Fiona Campbell http://orcid.org/0000-0001-6482-0743
Bonnie Stevens http://orcid.org/0000-0001-5387-2302
G. Allen Finley http://orcid.org/0000-0003-4579-7749
Jennifer Stinson http://orcid.org/0000-0002-9969-8052
Dawn Richards http://orcid.org/0000-0003-1151-0826
Elise Reiter http://orcid.org/0000-0002-6432-7913

© 2021 The Author(s). Published with license by Taylor & Francis Group, LLC.
This is an Open Access article distributed under the terms of the Creative Commons Attribution License (http://creativecommons.org/licenses/by/4.0/), which permits unrestricted use, distribution, and reproduction in any medium, provided the original work is properly cited.

### A systematic review and meta-analysis of bullying victimization and chronic pain

Teresa Marin[a], Jill A. Hayden[b], Rebecca Lewinson[a], Quenby Mahood[c], Debra Pepler[a], and Joel Katz[a]

[a]York University, Department of Psychology, Toronto, ON, Canada; [b]Dalhousie University, Department of Community Health and Epidemiology, Faculty of Medicine, Halifax, NS; [c]SickKids Hospital, Toronto, ON, Canada

**Introduction/Aim**: This systematic review examines the relationship between childhood bullying victimization and the development and persistence of pain, with a focus on the temporal nature of this relationship, as well as potential moderating factors.

**Methods**: We included prospective cohort studies of the relationship between childhood bullying victimization and pain. We included studies with a measure of victimization obtained in childhood (< 19 years) and pain outcomes (pain intensity, pain frequency, or the presence of a chronic pain condition) measured at least three months after. We searched Medline, EMBASE, PsycINFO, and CINAHL for relevant studies. We used standard methodological procedures consistent with Cochrane reviews of prognosis studies.

**Results**: We included four longitudinal studies (6,275 participants). The mean age of participants at baseline ranged from 10 to 14 years and the follow-up periods ranged from six months to 12 years. Two of the four studies were judged as having high risk of bias. Meta-analysis of results from four studies revealed increased risk of pain among victimized compared to non-victimized youth (adjusted OR [95% CI] = 1.45 [1.06-1.97], but the effect size was small and not clinically important.

**Discussion/Conclusions**: Study findings were limited by the dearth of evidence from prospective studies. Meta-analytic findings showed that victimization may incur some risk for later pain, although the evidence was judged to be very low quality. High quality studies that measure and report the nuances of bullying victimization and the presence or absence of bodily harm are needed to test the proposed moderator models.

© 2021 The Author(s). Published with license by Taylor & Francis Group, LLC.
This is an Open Access article distributed under the terms of the Creative Commons Attribution License (http://creativecommons.org/licenses/by/4.0/), which permits unrestricted use, distribution, and reproduction in any medium, provided the original work is properly cited.

### Chronic pain and the covid-19 pandemic: examining psychological distress, physical activity, and exercise self-efficacy

Valentina Mihajlovic[a], Madelaine Gierc[b], and Dean Tripp[c]

[a]Queen's University, Psychology, Kingston, Ontario, Canada; [b]University of British Columbia, Kinesiology, Vancouver, British Columbia, Canada; [c]Queen's University, Psychology, Anesthesiology & Urology, Kingston, Ontario, Canada

**Introduction/Aim**: Reduced physical activity (PA) during the COVID-19 ("COVID") pandemic may exacerbate the effects of psychological distress observed worldwide. However, little COVID-PA research has explored this effect on individuals experiencing chronic pain. Therefore, the present study examined psychological distress and PA in individuals with and without chronic pain during the first six months of the COVID pandemic. It was hypothesized that individuals with chronic pain would demonstrate significantly poorer outcomes. The present study also examined the effect of exercise self-efficacy (ESE) on PA during this time. It was hypothesized that individuals with chronic pain would report lower ESE than those without chronic pain, but that ESE would be positively associated with self-reported engagement in PA in both groups.

**Methods**: Two hundred and seventy-five individuals completed an online survey assessing COVID-related distress, general stress, mood, pain, PA, and ESE. One fifth of the sample self-identified as living with chronic pain.

**Results**: Relative to the non-chronic pain group, the chronic pain group reported significantly greater COVID-specific distress and general stress, and lower mood and ESE. No differences in PA were observed. ESE was not significantly associated with self-reported PA in the chronic pain group; however, this relationship was significant in the non-chronic pain group.

**Discussion/Conclusions**:Individuals with chronic pain appear to be disproportionately impacted by COVID and may benefit from targeted psychosocial supports. Engagement in PA was partially dependent on individuals' perceived ability to be physically active during COVID. Future research aimed at understanding COVID-specific barriers to PA is warranted.

**CONTACT** Valentina Mihajlovic valentina.mihajlovic@queensu.ca

© 2021 The Author(s). Published with license by Taylor & Francis Group, LLC.
This is an Open Access article distributed under the terms of the Creative Commons Attribution License (http://creativecommons.org/licenses/by/4.0/), which permits unrestricted use, distribution, and reproduction in any medium, provided the original work is properly cited.

### Preliminary analysis of coping strategies utilized in pediatric patients with complex pain

Sarah Moroziuk[a], Casey McMahon[b], Prosanta Mondal[c], and Krista Baerg[d]

[a]University of Saskatchewan, College of Medicine, Saskatoon, Saskatchewan, Canada; [b]University of Saskatchewan, Department of Pediatrics, Saskatoon, Saskatchewan, Canada; [c]University of Saskatchewan, Clinical Research Support Unit, Saskatoon, Saskatchewan, Canada; [d]University of Saskatchewan, Department of Pediatrics, Saskatoon, Saskatchewan, Saskatchewan Health Authority – Saskatoon, Canada

**Introduction/Aim**: Chronic pain affects 25-35% of youth and negatively impacts physical, psychological and social functioning. Many individuals adopt coping strategies to help mitigate the effects pain has on their daily lives. For patients attending the local Interdisciplinary Pediatric Complex Pain Clinic, a registry database was established to better understand how chronic pain is managed. Preliminary analysis aims to describe coping strategies utilized by patients and assess the relationship between coping strategies and functional disability, school attendance and pain interference.

**Methods**: Forty-four participants from the Pediatric Complex Pain Clinic consented to being a part of a larger registry database. This involved completing self-assessment measures upon intake. Demographics, parental report of school attendance, and scores from the Functional Disability Inventory (FDI), PROMIS Pediatric Pain Interference, and PedsQL Pain Coping Inventory were exported for preliminary analysis.

**Results**: Participants were aged 9.4–18.3 years (mean=14.6, SD=2.3), 32 (72%) were female. Mean school days missed per month was 8.1 (SD=8.5, range=1–25). PROMIS Pain Interference scores are strongly correlated with total FDI scores ($r=+0.75$; $p<0.0001$). Total Pain Coping is moderately correlated with PROMIS Pain Interference ($r=+0.44$; $p<0.005$), weakly correlated with total FDI ($r=+0.42$; $p<0.05$) and not correlated with days of school missed ($r=+0.04$; $p<0.83$).

**Discussion/Conclusions**: Preliminary analysis identified a relationship between total pain coping, pain interference and total FDI scores. It was expected that increased pain coping scores would be associated with lower pain interference and functional impairment. Further exploration is needed to identify specific coping strategies associated with decreased pain interference, functional disability and school absenteeism.

© 2021 The Author(s). Published with license by Taylor & Francis Group, LLC.
This is an Open Access article distributed under the terms of the Creative Commons Attribution License (http://creativecommons.org/licenses/by/4.0/), which permits unrestricted use, distribution, and reproduction in any medium, provided the original work is properly cited.

### Understanding the experiences of patients with pain using cannabis: a qualitative systematic review

Jeremy Y Ng, Amanda Abrams, and Ananya Pathak

Department of Health Research Methods, Evidence, and Impact, Faculty of Health Sciences, McMaster University, Hamilton, Ontario, Canada.

**Introduction/Aim:** Cannabis is becoming an increasingly common option utilized by patients to manage pain, despite this being supported by a low level of research evidence. It is imperative that healthcare providers, researchers, and patients are informed of the benefits and risks of using cannabis to manage pain. The aim of this qualitative systematic review was to understand the experiences of patients with pain using cannabis.

**Methods**: MEDLINE, EMBASE, AMED and CINAHL databases were searched from inception to May 2020. Eligible studies included those providing qualitative data relating to patient use of cannabis for the management of pain of any type. Relevant data was extracted from each eligible article, and a thematic analysis was performed to identify themes and subthemes.

**Results**: Of 1741 search results, 8 articles were deemed eligible and included in the present review. Four main themes emerged as follows: 1) difficulties with cannabis use, 2) advantages of cannabis use, 3) awareness and experimentation with cannabis, and 4) stigma and lack of knowledge.

**Discussion/Conclusions**: Our findings revealed that patients reported experiencing a number of side effects from using cannabis, but also felt that there were benefits associated with use in reducing their pain. Other topics identified by patients which warrant further exploration include strategies to destigmatize the use of cannabis for medical purposes, and safe and effective cannabis dosing for different pain conditions. Based on the limited number of qualitative studies identified, further research that examines the themes captured relating to the experiences of patients using cannabis for pain is warranted.

### ORCID

Jeremy Y Ng http://orcid.org/0000-0003-0031-5873

© 2021 The Author(s). Published with license by Taylor & Francis Group, LLC.
This is an Open Access article distributed under the terms of the Creative Commons Attribution License (http://creativecommons.org/licenses/by/4.0/), which permits unrestricted use, distribution, and reproduction in any medium, provided the original work is properly cited.

### A systematic review and quality assessment of complementary and alternative medicine mention and recommendations in neck pain clinical practice guidelines

Jeremy Y Ng and Mitali Uppal

Department of Health Research Methods, Evidence, and Impact, Faculty of Health Sciences, McMaster University, Hamilton, Ontario, Canada

**Introduction/Aim**: Patients experiencing neck pain (NP) commonly use complementary and alternative medicine (CAM), especially when standard treatment of care has failed to provide relief. Many clinicians lack the adequate knowledge and expertise to counsel patients surrounding the safe and effective use of CAM, and rely on evidence-based clinical practice guidelines (CPGs). The purpose of this review was to identify the quantity and assess the quality of CPGs for the treatment and/or management of NP.

**Methods**: Searches were conducted on MEDLINE, EMBASE and CINAHL databases, and the Guidelines International Network and the National Centre for Complementary and Integrative Health websites from 2009-2020. Eligible CPGs provided recommendations for the treatment and/or management of NP. Those found to contain CAM recommendations were assessed twice and in duplicate using the AGREE II instrument, once for the overall CPG and once for the CAM section.

**Results**: From 643 unique search results, 9 CPGs mentioned CAM, and 8 made CAM recommendations. Scaled domain percentages from highest to lowest were: scope and purpose (93.4% overall, 93.06% CAM), stakeholder involvement (81.6% overall, 81.94% CAM), rigour of development (70.83% overall, 66.27% CAM), clarity of presentation (64.93% overall, 60.76% CAM), editorial independence

(47.92% overall, 45.31% CAM), and applicability (39.32% overall, 33.59% CAM). Quality varied within and across guidelines.

**Discussion/Conclusions**: The majority of NP guidelines provided CAM recommendations, however, their quality are lower than overall recommendations across a number of domains. Clinicians may consult the CPGs identified by this review to understand what CAMs are recommended for the treatment/management of NP.

**ORCID**

Jeremy Y Ng http://orcid.org/0000-0003-0031-5873

© 2021 The Author(s). Published with license by Taylor & Francis Group, LLC.
This is an Open Access article distributed under the terms of the Creative Commons Attribution License (http://creativecommons.org/licenses/by/4.0/), which permits unrestricted use, distribution, and reproduction in any medium, provided the original work is properly cited.

### Baseline predictors of a disability at 3-month after back pain: A comparative analytical view of minimal clinically important change in disability versus deficit in this change

Zakir Uddin, Arthur Woznowski-Vu, and Timothy H. Wideman

McGill University, School of Physical and Occupational Therapy, Montreal, Quebec, Canada

**Introduction/Aim:** Prognostic factor detection is an important focus in back pain research since a complicated relationship exists between physical activity and disability without empirical evidence. A group of back pain patients may not have a minimal clinically important change (MCIC) in disability. The study is aimed to detect the baseline predictors of disability at 3-month after back pain by comparing MCIC in disability and MCIC deficit groups and examine whether pain experience (severity and interference) can predict disability beyond psychological, sensitivity, sociodemographic and personal factors.

**Methods:** This study included 106 adult participants with back pain<6 months in a longitudinal observational design (baseline lab session and outcome evaluation at 3-months).

The measures are disability outcome, pain experience (severity and interference), psychological factors, pressure pain sensitivity, and sociodemographic and personal factors. Based on pain disability index (PDI) change cut-off, the two groups: (1) PDI change ≥ 9.5 (MCIC); (2) PDI change< 9.5 (deficit in MCIC). T-test, correlation and binary logistic regression analyses compared the predictive value of disability in MCIC and MCIC deficit groups. The hierarchical regression analysis evaluated whether pain experience variables contributed to unique predictive value beyond other baseline factors.

**Results:** The group differences (between MCIC in disability and MCIC deficit groups) on pain duration ($p<.05$), pain affective interference ($p<.05$), pain physical interference ($p<.01$). The predictors of reporting deficit in MCIC of PDI were pain severity (OR 1.64, 95% CI:1.11-2.44) and pain physical interference (OR .66, 95% CI: .47-.92). The significant predictors of disability (in total sample) were pain duration ($\beta = .179$, $t = 2.121$, $p < .05$) and pain severity ($\beta = .482$, $t = 3.759$, $p < .01$). The pain experience variables contributed an additional 15.4% variance beyond the other baseline factors.

**Discussion/Conclusions:** Pain severity is the unique predictor of disability for the MCIC deficit group and total sample beyond the other baseline factors. Pain physical interference is a predictor of reporting deficit in MCIC of disability. Pain duration also a predictor of disability at 3-month after back pain. There are theoretical and clinical implications of these findings.

© 2021 The Author(s). Published with license by Taylor & Francis Group, LLC.
This is an Open Access article distributed under the terms of the Creative Commons Attribution License (http://creativecommons.org/licenses/by/4.0/), which permits unrestricted use, distribution, and reproduction in any medium, provided the original work is properly cited.

### Examining the Relationships Between Neonatal Pain-Related Facial Actions and Cortical Activity

Oana Bucsea[a], Ilana Shiff[a], Laura Jones[b], Lorenzo Fabrizi[b], Maria Fitzgerald[b], Mohammed Rupawala[b], Kimberley Whitehead[b], Maria Pureza Laudiano-Dray[b], and Rebecca Pillai Riddell[b]

[a]York University, Psychology, Toronto, Ontario, Canada; [b]University College London, Neuroscience, Physiology and Pharmacology, London, UK

**Introduction/Aim**: Past research examining the links between neonates' pain-related cortical activity and facial actions has yielded mixed results. Using a more comprehensive approach to the measurement of both pain-related facial actions and cortical activity, the goal of the current study was to gain a better understanding of how these two pain-related responses are related in preterm and full-term neonates.

**Methods**: Participants consisted of 41 late preterm and 37 full-term neonates, ranging in age from 0-14 days. Pain-related facial actions were micro-coded on a second-by-second basis using three constellations of facial actions to assess which is most optimal for capturing the full range of neonates' pain-related facial expressions. Cortical activity was assessed using Global Field Power (GFP), a measurement of activity across the whole EEG array on the scalp.

**Results**: Results indicated that a cluster of three pain-related facial actions (brow bulge, eye squeeze, and naso-labial furrow), coded on a second-by-second basis, best captured the distribution in neonates' pain-related facial expressions. GFP analyses showed that differences in pain-related facial activity were linked to distinctions in underlying cortical processes, a topic which has been disputed due to mixed findings yielded by previous studies.

**Discussion/Conclusions**: This study has implications for future research practices examining neonatal pain-related brain-behaviour relationships. Employing these more comprehensive measurements of cortical and behavioural pain responses in research will be integral for furthering our understanding of brain-behaviour relationships in hospitalized infants frequently exposed to painful medical procedures, ultimately laying the foundation for the development of evidence-based pain assessment tools in infancy.

### ORCID

Rebecca Pillai Riddell http://orcid.org/0000-0003-3990-3680

© 2021 The Author(s). Published with license by Taylor & Francis Group, LLC.
This is an Open Access article distributed under the terms of the Creative Commons Attribution License (http://creativecommons.org/licenses/by/4.0/), which permits unrestricted use, distribution, and reproduction in any medium, provided the original work is properly cited.

### Pain expectancies, altered pain sensitivity and work status in individuals with chronic low back pain

Junie S. Carriere[a], Blake Boehme[b], Marie-France Coutu[c], Marie-José Durand[c], Marise Cornelius[a], and Robert R. Edwards[a]

[a]Brigham and Women's Hospital Pain Management Center, Harvard Medical School, Department of Anesthesiology, Boston, Massachusetts, USA; [b]York University, Department of Psychology, North York, Ontario, Canada; [c]Université de Sherbrooke, Center for Action in Work Disability Prevention and Rehabilitation, Hôpital Charles-LeMoyne Research Center, Longueuil, Quebec, Canada

**Introduction/Aim**: Research has shown that pain expectancies are strongly associated with work disability in individuals with chronic pain. However, little is known about the psychophysical processes by which pain expectancies may impact work disability. This cross-sectional study assessed the association between pain expectancies, temporal summation of pain and work status in a sample of individuals with chronic low back pain. Temporal summation of pain is a primary marker for central nervous system sensitization.

**Methods**: This study consisted of 301 chronic low back pain patients recruited from the Pain Management Center at Brigham and Women's Hospital in Chestnut Hill, Massachusetts. Participants had a diagnosis of back pain with or without radicular symptoms and reported experiencing pain for at least 6 months. Participants completed self-report sociodemographic measures, as well as measures of current pain intensity, pain expectancies and current work status before undergoing standardized psychophysical pain-testing procedures designed to assess temporal summation of mechanical pain.

**Results**: Pearson correlations and independent samples t-tests revealed that pain expectancies were significantly associated with temporal summation of pain and work status in chronic low back pain patients. Logistic regression analyses demonstrated that pain sensitivity mediated the relationship between pain expectancies and work status in chronic low back pain patients.

**Discussion/Conclusions**: The findings from this study suggest that altered pain sensitivity may be a mechanism by which pain expectancies are associated with work status in chronic low back pain patients. Our findings hint at a potential synergistic effect of psychological and physiological processes in shaping individuals' risk for disability. These findings are limited by the correlational nature of this study.

**ORCID**

Blake Boehme http://orcid.org/0000-0002-6972-8695

© 2021 The Author(s). Published with license by Taylor & Francis Group, LLC.
This is an Open Access article distributed under the terms of the Creative Commons Attribution License (http://creativecommons.org/licenses/by/4.0/), which permits unrestricted use, distribution, and reproduction in any medium, provided the original work is properly cited.

### The role of presurgical pain expectancies in predicting postoperative opioid use and dose following total knee arthroplasty

Junie S. Carriere[a], Marc Olivier Martel[b], Sabine Bou-Saba[a], Marise Cornelius[c], Claudia Campbell[c], Jennifer A. Haythornthwaite[a], and Robert R. Edwards[a]

[a]Brigham and Women's Hospital Pain Management Center, Harvard Medical School, Department of Anesthesiology, Boston, Massachusetts, USA; [b]American University of Beirut, Department of Psychology, Beirut, Lebanon; [c]Johns Hopkins University School of Medicine, Department of Psychiatry & Behavioral Sciences, Baltimore, Maryland, USA

**Introduction/Aim**: Research has accumulated to suggest that expectancies are a risk factor for poor recovery outcomes in individuals with severe knee osteoarthritis. Although a number of studies have examined the relationship between expectancies and postsurgical pain and function, little research has examined the influence of expectancies on opioid use. The present study examined the role of pain expectancies in the prediction of postoperative opioid use and dose in individuals underdoing total knee arthroplasty.

**Methods**: The study sample consisted of 308 individuals (59.5% women) with end-stage osteoarthritis of the knee who were scheduled for a total knee arthroplasty at the Johns Hopkins Department of Orthopedic Surgery or the Brigham and Women's Hospital. Participants completed measures of preoperative pain severity, disability, opioid use and pain expectancies. Postoperative data was collected regarding opioid use and dose 3 days following surgery.

**Results**: Pearson correlations and independent samples t-tests revealed that preoperative pain expectancies were significantly associated with postoperative opioid use and dose. Logistic regression analyses revealed that preoperative pain expectancies significantly predicted postoperative opioid use 3 days following surgery, and multiple regression analyses revealed that preoperative pain expectancies predicted opioid dose 3 days following surgery. Regression analyses controlled for age, sex, body-mass index, surgery duration, preoperative pain and function and preoperative opioid use.

**Discussion/Conclusions**: The findings of this study suggest that increasing our understanding of the role of pain expectancies in individuals with severe knee osteoarthritis might contribute to the development of novel interventions aimed at promoting successful recovery after total knee arthroplasty.

**ORCID**

Jennifer A. Haythornthwaite http://orcid.org/0000-0002-5924-7580

© 2021 The Author(s). Published with license by Taylor & Francis Group, LLC.
This is an Open Access article distributed under the terms of the Creative Commons Attribution License (http://creativecommons.org/licenses/by/4.0/), which permits unrestricted use, distribution, and reproduction in any medium, provided the original work is properly cited.

### Promoting safer opioid prescribing with cost-free access to an accredited online opioid self-assessment program

Yalnee Shantharam[a], Metasebia Assefa[a], Ralph Fabico[a], John Flannery[a], and Andrea Furlan[b]

[a]Toronto Rehabilitation Institute-University Health Network, Toronto, Ontario, Canada; [b]University of Toronto, Department of Medicine, Toronto, Ontario, Canada

**Introduction/Aim**: The Online Opioid Self-Assessment Program (SAP) is a 3 hour accredited course that was created using the recommendations in the 2017 Canadian Opioid Guideline. This program has been distributed to all Canadian provinces at no cost, to prescribers, learners and educators including remote areas. In this study, we examined the pre and post test scores of participants completing the Online Opioid

SAP to measure whether there was an improvement in scores after participation in the program.

**Methods**: This accredited program was developed using five assessment areas that include general knowledge, titration of opioids, optimization of non-opioid treatments, tapering/switching/rotating opioids, and prevention of opioid use disorder and overdose. The program was promoted through targeted emails to university program directors, physician email databases, and shared through personal networks and social media platforms. Participants were required to complete a baseline pre-test, the 3 hour Online Opioid SAP and a post-test following completion of the program.

**Results**: From October 1, 2019-October 31, 2020, 545 participants have enrolled in the Online Opioid SAP. We collected 201 completed pre and post-test participant data. The mean pre-test scores were 45.7% and the mean post-test scores were 78.1%, and this change was significant at $p < 0.05$.

**Discussion/Conclusions**: This study shows a significant improvement in participant knowledge from baseline scores to the post-test after completing the program. Free, remote access to the online program has made it possible for prescribers and learners to learn about safer opioid prescribing without the barrier of location and cost.

### ORCID

John Flannery http://orcid.org/0000-0003-3924-4283
Andrea Furlan http://orcid.org/0000-0001-6138-8510

© 2021 The Author(s). Published with license by Taylor & Francis Group, LLC.
This is an Open Access article distributed under the terms of the Creative Commons Attribution License (http://creativecommons.org/licenses/by/4.0/), which permits unrestricted use, distribution, and reproduction in any medium, provided the original work is properly cited.

### Antinociceptive effects of extract of *Stemodia maritima* and the diterpene stemodin in a rodent model of temporomandibular joint inflammatory pain

Hellíada Vasconcelos Chaves[a], Maria Ester Frota Fernandes[b], Mirna Marques Bezerra[a], Francisco Isaac Fernandes Gomes[a], José Leonaldo Miranda Azevedo[c], Daniele Rocha do Val[d], Felipe Dantas Silveira[e], Dina Andressa Martins Monteiro[a], Sebastião Carlos de Sousa Oliveira[a], Francisco Geraldo Barbosa[f], Jair Mafezoli[f], Virgínia Cláudia Carneiro Girão[c], Vicente de Paulo Teixeira Pinto[g], and Barry John Sessle[a]

[a]Federal University of Ceará, Faculty of Dentistry, Sobral, Ceará, Brazil; [b]Federal University of Ceará, Health Sciences Graduate Programme, Sobral, Ceará, Brazil; [c]Federal University of Ceará, Morphofunctional Sciences Graduate Programme, Fortaleza, Ceará, Brazil; [d]Federal University of Ceará, Morphofunctional Sciences Graduate Programme, Fortaleza, Ceará, Brazil; [e]Federal University of Ceará, Dentistry Graduate Programme, Fortaleza, Ceará,Brazil; [f]Federal University of Ceará, Dentistry Graduate Programme, Fortaleza, Ceará,Brazil; [g]Federal University of Ceará, Dentistry Graduate Programme, Fortaleza, Ceará,Brazil

**Introduction/Aim**: Inflammatory-related pain of the temporomandibular joint (TMJ) involves complex pathophysiological mechanisms, and it is difficult to treat. The ethanolic extract of the plant *Stemodia maritima* (ESM) has been used as an antiinflammatory agent, however its potential as an analgesic agent has not been studied. This study evaluated the antinociceptive effects of ESM and the isolated compound stemodin in a rat model of TMJ pain.

**Methods**: Male Wistar rats were pre-treated with ESM (0.1, 1, or 10 μg/kg; p.o.), stemodin (0.1 or 1 μg/kg; p.o.) or vehicle 60 minutes before TMJ formalin injection, and formalin-evoked nociceptive behaviours of scratching and head flinches were quantified. A plasma extravasation assay of TMJ periarticular inflammation, and the nociceptive behavior related to pharmacological inhibition of heme oxygenase-1and nitric oxide (NO) pathways and opioid receptors were evaluated, and an ELISA assay of IL-1β levels in the trigeminal ganglion was also performed. Normality was evaluated by the Shapiro-Wilk test. The homogeneity of variances for all variables was tested by Levene's test, followed by a post-hoc test where indicated.

**Results**: ESM and stemodin pre-treatments significantly reduced periarticular plasma extravasation and attenuated nociceptive behaviour in a dose-dependent manner ($p<0.05$). Pharmacological inhibition of HO-1 and NO pathways as well as opioid receptors did not prevent the antinociception evoked by ESM and stemodin ($p>0.05$), but did decrease IL-1β levels in the trigeminal ganglia ($p<0.05$).

**Discussion/Conclusions**: ESM and stemodin afforded antinociception and anti-inflammatory effects in an experimental model of TMJ pain via inhibition of the levels of IL-1β.

© 2021 The Author(s). Published with license by Taylor & Francis Group, LLC.
This is an Open Access article distributed under the terms of the Creative Commons Attribution License (http://creativecommons.org/licenses/by/4.0/), which permits unrestricted use, distribution, and reproduction in any medium, provided the original work is properly cited.

### Executive functioning in youth with and without chronic pain: a comparative analysis

Kailyn Turner[a], Gabrielle Wilcox[a], David Nordstokke[a], Bruce Dick[b], Meadow Schroeder[a], and Melanie Noel[c]

[a]University of Calgary, Werklund School of Education, Calgary, Alberta, Canada; [b]University of Alberta, Departments of Anesthesiology & Pain Medicine, Psychiatry, and Pediatrics, Edmonton, Alberta, Canada; [c]University of Calgary, Department of Psychology, Calgary, Alberta, Canada

**Introduction/Aim**: Preliminary research in youth with chronic pain suggests differences in attention and working memory, which has been similarly demonstrated in adults with chronic pain. There is little research on other aspects of executive functioning (EF) in this population despite its critical role in problem solving, school functioning, and coping. This study aimed to examine differences in several aspects of EF between youth with and without chronic pain using performance-based neuropsychological tests and a behaviour rating scale.

**Methods**: Participants completed ratings of pain; physical, emotional, social, and school functioning; sleep quality; medication; and a general intelligence screener. Standardized tests were used to examine EF with a focus on working memory, divided and alternating attention, inhibition, flexibility, incidental memory, and planning. A parent- and self-report behaviour rating of EF was administered.

**Results**: Recruitment from two tertiary-care pain clinics in Western Canada resulted in a sample of 26 youth with chronic pain (80.8% girls) and their parents. A comparison group of 30 youth without chronic pain and their parents were recruited from the community. Participants with chronic pain had significantly lower scores on several performance-based tests of working memory/divided attention, inhibition, and flexibility/alternating attention than the comparison group. Statistically significant group differences were found on behaviour ratings of emotion and cognitive regulation as well as global EF. Covariate analyses were conducted where there were significant group differences and several observed group differences remained.

**Discussion/Conclusions**: This study is one of few multidimensional examinations of EF in youth with chronic pain, using performance-based tests combined with behaviour ratings. Our findings suggest EF differences in youth with chronic pain, across a variety of domains and may suggest risk for specific cognitive processing weaknesses in this population. Medical and educational teams should work towards identification and compensatory support for EF within individualized pain management for youth.

### ORCID

Bruce Dick http://orcid.org/0000-0003-0404-4927
Meadow Schroeder http://orcid.org/0000-0002-1077-7855
Melanie Noel http://orcid.org/0000-0003-3752-8055

© 2021 The Author(s). Published with license by Taylor & Francis Group, LLC.
This is an Open Access article distributed under the terms of the Creative Commons Attribution License (http://creativecommons.org/licenses/by/4.0/), which permits unrestricted use, distribution, and reproduction in any medium, provided the original work is properly cited.

### Abnormal subgenual anterior cingulate cortex functional connectivity in carpal tunnel syndrome is influenced by sex

Natalie R. Osborne[a], Junseok A. Kim[a], Lee B. Kisler[b], Camille Fauchon[b], Rima El-Sayed[c], Dimitri J. Anastakis[d], and Karen D. Davis[e]

[a]Krembil Brain Institute, University Health Network, Institute of Medical Science, University of Toronto, Toronto, Canada; [b]Krembil Brain Institute, University Health Network, Toronto, Canada; [c]Krembil Brain Institute, University Health Network, Institute of Medical Science, University of Toronto, Toronto, Canada; [d]Department of Surgery and Institute of Medical Science, University of Toronto, Hand Program, Toronto Western Hospital, Toronto, Canada; [e] Krembil Brain Institute, University Health Network, Institute of Medical Science, and Dept. Surgery, University of Toronto, Toronto, Canada

**Introduction/Aim**: The subgenual anterior cingulate cortex (sgACC) is a key node of the descending antinociceptive system that shows sex-differences in its functional connectivity (FC) in healthy and chronic pain states. In a male-prevalent chronic pain condition, sgACC FC was found to be abnormal only in women. The aim of this study was to characterize sgACC FC in men and women with a female-dominant chronic pain condition, carpal tunnel syndrome (CTS).

**Methods**: Resting state fMRI was collected from people with CTS (n=26, 19 women; 7 men) and healthy controls (HCs, n=26, 19 women; 7 men). sgACC to whole brain functional connectivity was calculated and compared between CTS and HCs with sex included as a covariate.

Two subsequent sex-disaggregated exploratory analyses compared sgACC FC in CTS patients to same sex controls.

**Results**: sgACC FC with the frontal pole, dorsomedial, and dorsolateral prefrontal cortex (PFC) was reduced in CTS compared to HCs. When we stratified the analysis by sex, we discovered that this abnormality was driven by men with CTS.

**Discussion/Conclusions**: These findings suggest that men with CTS (a female-dominant condition) have reduced sgACC FC to the prefrontal cortex compared to healthy men. A similar pattern of reduced sgACC FC to the PFC was seen in women with a male-prevalent chronic pain condition when compared to healthy women. Due to its role in pain processing and coping, and the abnormalities in its circuitry found here, the sgACC should be considered for further study as a potential therapeutic target for neuromodulation in chronic pain.

### ORCID

Karen D. Davis http://orcid.org/0000-0003-1879-0090

© 2021 The Author(s). Published with license by Taylor & Francis Group, LLC.
This is an Open Access article distributed under the terms of the Creative Commons Attribution License (http://creativecommons.org/licenses/by/4.0/), which permits unrestricted use, distribution, and reproduction in any medium, provided the original work is properly cited.

### Interventions for depressive symptoms in people with chronic pain: a systematic review of meta-analyses

Darren K. Cheng[a], Ka Sing Paris Lai[b], Oscar Javier Pico-Espinosa[c], Danielle B. Rice[d], Chadwick Chung[e], Golale Modarresi[f], and Abhimanyu Sud[g]

[a]Sinai Health, Lunenfeld-Tanenbaum Research Institute, Bridgepoint Collaboratory for Research and Innovation, Toronto, ON, Canada; [b]Western University, Department of Psychiatry, London, ON, Canada; [c]Karolinska Institutet, Institute of Environmental Medicine, Solna, Sweden; [d]McGill University, Department of Psychology, Montreal, QC, Canada; [e]Canadian Memorial Chiropractic College, Toronto, ON, Canada; [f]University of Toronto, Centre for Addiction and Mental Health, Department of Psychiatry, Toronto, ON, Canada; [g]University of Toronto, Department of Family and Community Medicine, Toronto ON, Canada

**Introduction/Aim**: Depression is common in chronic pain, but evidence-based guidance for management is limited. This review of reviews synthesizes the effectiveness of clinical interventions for improving depressive symptoms in people with all types of chronic pain.

**Methods**: Seven electronic databases were searched on September 15, 2020. English-language systematic reviews and meta-analyses of controlled trials that reported depression outcomes for all kinds of clinical interventions on depressive outcomes in chronic pain were included. Two independent reviewers screened, extracted, and assessed quality. Data were summarized visually and narratively using standardized mean differences and 95% confidence intervals as the primary outcome of interest.

**Results**: Eighty-three reviews were selected, including 185 meta-analyses. A large proportion of reviews investigated fibromyalgia or mixed chronic pain. Psychological interventions were most commonly evaluated, followed by pharmacological and mind-body interventions. Mind-body interventions for axial pain and fibromyalgia, acceptance and commitment therapy for general chronic pain, and fluoxetine and web-based psychotherapy for fibromyalgia showed the most robust effects. Exercise for arthritis, pharmacotherapy for neuropathic pain, self-regulatory psychological for axial pain, and music therapy for general chronic pain showed large, significant effects, but estimates were derived from low- or critically low-quality reviews.

**Discussion/Conclusions**: No single intervention type demonstrates substantial superiority across different pain populations. Other dimensions beyond efficacy, such as individual preference, accessibility, safety, cost, and efficacy for non-depressive outcomes should be weighed when considering treatment options. Further effectiveness research is required for common pain types such as arthritis and axial pain, and common interventions such as opioids, anti-inflammatories and acupuncture.

### ORCID

Abhimanyu Sud http://orcid.org/0000-0001-8354-6153

© 2021 The Author(s). Published with license by Taylor & Francis Group, LLC.
This is an Open Access article distributed under the terms of the Creative Commons Attribution License (http://creativecommons.org/licenses/by/4.0/), which permits unrestricted use, distribution, and reproduction in any medium, provided the original work is properly cited.

### Genetic and pharmacological manipulation of D1 receptors in the ACC alters mechanical sensory thresholds and excitatory synaptic transmission

Soroush Darvish-Ghane[a], Clémentine Quintana[b], Jean Martin Beaulieu[c], and Loren J. Martin[a,c]

[a]Department of Cell and Systems Biology, University of Toronto, Toronto, ON M5S 3G5, Canada; [b]Department of Pharmacology and Toxicology, University of Toronto, Toronto, ON, Canada; [c]Dept. of Psychology, University of Toronto Mississauga, Mississauga, ON M9A1C5 Canada

**Introduction**: The anterior cingulate cortex (ACC) is one of the primary cortical regions involved in the affective component of pain. While evidence suggests that dopamine in the ACC reduces pain and synaptic transmission, the role of dopamine D1-like receptors remains elusive. Hence, we wanted to directly determine the contribution of ACC D1-like receptors in the modulation of sensory thresholds and synaptic activity.

**Methods**: We characterized sensory thresholds in naïve mice and mice injected with CFA following Crispr/Cas9 mediated somatic knockout of the D1 receptor (D1R) in all neuronal subtypes of the ACC. We also used slice electrophysiology to characterize the pharmacological modulation of excitatory synaptic transmission by D1-like receptors.

**Results**: Behavioural testing revealed decreased mechanical sensitivity thresholds in mice with Crispr/Cas9 knockout of D1Rs, while locomotion and anxiety phenotypes remained unchanged. By using whole-cell patch clamping on ACC brain slices from mice, we observed an inhibitory role for D1-like agonists on AMPAR mediated currents, mimicking the effects of DA on EPSCs. The inhibitory effect of D1 agonists was not blocked by D1 antagonists, but rather co-application of both ligands resulted in a synergistic and robust inhibition of AMPAR currents, suggesting potential novel effects for these drugs.

**Discussion/Conclusions**: These data provide the first evidence that D1Rs in the ACC modulate peripheral sensitivity to mechanical stimuli based on somatic genetic manipulations. In addition, correlational pharmacological data for D1Rs have revealed a potential novel inhibitory mechanism for D1Rs in the cingulate cortex on basal glutamatergic transmission.

© 2021 The Author(s). Published with license by Taylor & Francis Group, LLC.
This is an Open Access article distributed under the terms of the Creative Commons Attribution License (http://creativecommons.org/licenses/by/4.0/), which permits unrestricted use, distribution, and reproduction in any medium, provided the original work is properly cited.

### Neural mechanisms underlying sex differences in orofacial pain

Sukhbir Kaur[a], Hanna McDonald[b], Angela Lopez-Ramirez[c], Taylor Hickman[d], and Dayna L. Averitt[e]

[a]Texas Woman's University, Department of Biology, Denton, Texas, United States; [b]University of North Texas Health Science Center, Department of Medical Science, Fort Worth, Texas, United States; [c]Texas Woman's University, Department of Biology, Denton, Texas, United States; [d]Texas Woman's University, Department of Biology, Denton, Texas, United States; [e]Texas Woman's University, Department of Biology, Denton, Texas, United States

**Introduction/Aim:** Craniofacial pain disorders involving trigeminal sensory neurons disproportionately affect women and are possibly modulated by estrogen (E2) and peripheral serotonin (5HT). 5HT2A receptors co-express with transient receptor potential vanilloid 1 (TRPV1) ion channels in male rat trigeminal neurons and can sensitize TRPV1 causing enhanced calcium influx and calcitonin gene-related peptide (CGRP) release. It is unknown whether this is comparable in female rats. Previously, we found that peripheral 5HT evokes greater orofacial pain behaviors in female rats during stages of estrous cycle when E2 fluctuates rapidly. Thus, we hypothesized that **5HT2A and TRPV1 mRNA co-express in female trigeminal neurons, and E2 enhances 5HT potentiation of TRPV1 activity.**

**Methods:** 5HT-evoked orofacial nocifensive behaviors were quantified in male and female rats after M100907 pretreatment (5HT2A antagonist). Trigeminal ganglia sections were processed for *in situ* hybridization using 5HT2A and TRPV1 mRNA oligo-probes. Calcium influx and CGRP release were quantified in trigeminal neuron cultures after 5HT+E2 or M100907 pretreatment. Transcriptome changes post persistent vs acute E2 treatments were analyzed by RNA sequencing.

**Results:** Blocking peripheral 5HT2A receptors reduced 5HT-evoked nocifensive behaviors in females, not males. 5HT2A and TRPV1 mRNA co-expressed on female trigeminal neurons. Preliminary *in vitro* data indicate E2 pretreatment enhanced capsaicin-evoked calcium influx whereas M100907 pretreatment reduced capsaicin-evoked CGRP release. Several genes related to 5HT and TRPV1 were altered depending on E2 concentration.

**Discussion/Conclusions:** 5HT acts at 5HT2A receptors expressed on TRPV1 population of trigeminal sensory neurons and can be modulated by E2. This mechanism may underlie sex differences in trigeminal pain.

© 2021 The Author(s). Published with license by Taylor & Francis Group, LLC.
This is an Open Access article distributed under the terms of the Creative Commons Attribution License (http://creativecommons.org/licenses/by/4.0/), which permits unrestricted use, distribution, and reproduction in any medium, provided the original work is properly cited.

### Atlantic Canadian Mothers Knowledge and Use of Infant Pain Management

Brianna Richardson[a], Justine Dol[b], Dr. Marsha Campbell-Yeo[c], Dr. Megan Aston[d], Dr. Gail Tomblin Murphy[e], and Dr. Douglas McMillan[f]

[a]School of Nursing, Dalhousie University; Centre for Pediatric Pain Research, IWK Health Centre, Halifax, NS, Canada; [b]Faculty of Health, Dalhousie University, Halifax, NS, Canada; [c]School of Nursing, Dalhousie University; Centre for Pediatric Pain Research, IWK Health Centre, Halifax, NS, Canada; [d]School of Nursing, Dalhousie University, Halifax, NS, Canada; [e]VP Research, Innovation and Discovery & Chief Nurse Executive, Nova Scotia Health Authority, Halifax, NS, Canada; [f]Division of Neonatal Perinatal Medicine, Department of Pediatrics, Faculty of Medicine, Dalhousie University and IWK Health Centre, Halifax, NS, Canada

**Introduction/Aim**: All newborns endure painful procedures (e.g. vaccinations) and parents can effectively reduce pain (e.g. by breastfeeding), yet parents may not be aware of effective comfort strategies. With no in-depth evaluations of newborn pain management knowledge in parents from the Maritimes, we aimed to understand the current knowledge base for these mothers during the first six-months after birth.

**Methods**: In 2019, mothers (18 years+) with a newborn less than 6 months of age and from Nova Scotia (NS), New Brunswick (NB), or Prince Edward Island (PEI) completed an online survey that included a 10-item true/false questionnaire about newborn pain management knowledge and additional questions about pain management use. Descriptive statistics and comparative analyses (e.g. ANOVA) were conduct as appropriate.

**Results**: In total, 561 mothers from NS (46.5%), NB (30.5%) and PEI (23.0%) completed the survey. Maritime mothers had an average knowledge score of 6.80 (SD= 1.50). On average, more mothers knew breastfeeding was an effective comfort strategy (89.8%) but less knew about the pain-relieving effects of sucrose (41.1%). There was no significant difference in knowledge scores across provinces or other demographic factors. Mothers reported that they or their partners have previously used skin-to-skin contact (84.8%), followed by breastfeeding (65.6%) and oral sucrose (20.1%) for procedural pain management.

**Discussion/Conclusions**: This study provides insight into Atlantic Canadian mothers' current knowledge and use of pain management strategies. Preliminary analysis suggests that mothers from the Maritime provinces have a moderate understanding and use of procedural pain management in newborns.

© 2021 The Author(s). Published with license by Taylor & Francis Group, LLC.
This is an Open Access article distributed under the terms of the Creative Commons Attribution License (http://creativecommons.org/licenses/by/4.0/), which permits unrestricted use, distribution, and reproduction in any medium, provided the original work is properly cited.

### Impact of E-Learning Modules on Family Physicians' Self-efficacy Vis-à-vis Low Back Pain Patients

Évelyne Théberge[a], Benoit Martin[b], Anne Marie Pinard[c], and Danie Saucier[d]

[a]Physical Medicine and Rehabilitation, Laval University, Physical Medicine and Rehabilitation Department, Québec, Québec, Canada; [b] Physical Medicine and Rehabilitation, Laval University, Physical medicine and Rehabilitation Department, Québec, Québec, Canada; [c] Associate Professor, Laval University, Anesthesiology and Intensive Care Department, Québec, Québec, Canada; [d] Laval University, Physical Medicine and Rehabilitation Department, Institut de réadaptation en déficience physique de Québec, Québec, Québec, Canada

**Introduction/Aim**: Low back pain (LBP) is a highly prevalent condition worldwide (1). A variety of educational tools are available to physicians for the management of LBP, but many are time consuming. A possible alternative is an e-learning using short educational modules (2,3,4).

The purpose of this study is to demonstrate that a short e-learning tool is likely to increase the sense of competency of primary care physicians in the management of LBP.

**Methods**: Twenty one primary care physicians were recruited and 9 completed all surveys. They answered surveys about self-efficacy regarding LBP before ($T_0$), immediately after ($T_1$) and 3 months after ($T_2$) completion of four online modules and about modules' acceptability at $T_1$ . These surveys are adapted from the Continuing Professional Development Reaction (CPD-Reaction) tool (5) and from the Learning Object Review Instrument (LORI) (6). Each item is assessed using a 5-point likert scale (7). Date was dichotomized: scores 1 to 3 were associated with a negative sense of competency; the scores 4 and 5 with a positive feeling.

**Results**: The modules and the learning method were appreciated by the participants. Data shows that at T0, the sense of competency was moderate, and completion of the modules increases the sense of competency. This improvement persisted at 2 months.

**Discussion/Conclusions**: Using an e-Learning modules on a common pathology such as LBP tends to improve the sense of competency of primary care physicians. These online learning modules are a feasible, appreciated and useful tools and might be a simple and effective method to improve self-efficacy and in turn, patient care.

### References

(1) Hoy, D., et al. (2010). "The Epidemiology of low back pain." Best Pract Res Clin Rheumatol 24(6): 769–781.

(2) Richmond H, Hall AM, Hansen Z, Williamson E, Davies D, Lamb SE. Using mixed methods evaluation to assess the feasibility of online clinical training in evidence based interventions: a case study of cognitive behavioural treatment for low back pain. BMC medical education. 2016;16:163.

(3) Jacobs ZG, Elnicki DM, Perera S, Weiner DK. An E-learning Module on Chronic Low Back Pain in Older Adults: Effect on Medical Resident Attitudes, Confidence, Knowledge, and Clinical Skills. Pain medicine (Malden, Mass). 2018;19(6):1112–20.

(4) Ellaway, R. and K. Masters (2008). "AMEE Guide 32: e-Learning in medical education Part 1: Learning, teaching and assessment." Med Teach 30(5): 455–473.

(5) Legare F, Borduas F, Freitas A, Jacques A, Godin G, Luconi F, et al. Development of a simple 12-item theory-based instrument to assess the impact of continuing professional development on clinical behavioral intentions. PloS one. 2014;9(3):e91013.

(6) Tracey LL, John CN. A framework for evaluating the quality of multimedia learning resources. Journal of educational technology & society. 2007;10(2):44–59.

(7) Sullivan GM, Artino AR, Jr. Analyzing and interpreting data from likert-type scales. Journal of graduate medical education. 2013;5(4):541–2.

© 2021 The Author(s). Published with license by Taylor & Francis Group, LLC.
This is an Open Access article distributed under the terms of the Creative Commons Attribution License (http://creativecommons.org/licenses/by/4.0/), which permits unrestricted use, distribution, and reproduction in any medium, provided the original work is properly cited.

### Designing and evaluating VR games for Phantom Limb Pain management

Xin Tong[a], Xinxing Wang[b], Yiyang Cai[c], Owen Williamson[d], Bifa Fan[e], Diane Gromala[f], and Kunlin Wei[g]

[a]Simon Fraser University, School of Interactive Arts and Technology, BC, Canada; [b]China-Japan Friendship Hospital, Department, Beijing, China; [c]Peking University, School of Psychological and Cognitive Sciences, Beijing, China; [d]Monash University, Department of Epidemiology and Preventive Medicine, Melbourne, Australia; [e]China-Japan Friendship Hospital, Department, Beijing, China; [f]Simon Fraser University, School of Interactive Arts and Technology, BC, Canada; [g]Peking University, School of Psychological and Cognitive Sciences, Beijing, China

**Introduction/Aim**: Phantom limb pain (PLP) is a type of chronic pain that follows limb amputation, brachial plexus avulsion injury, or spinal cord injury. Treating PLP is a well-known challenge. Currently, virtual reality (VR) interventions are attracting increasing attention because they show promising analgesic effects. However, most previous studies of VR interventions were conducted with a limited number of patients in a single trial. Few studies explored questions such as how multiple VR sessions might affect pain over time, or if a patient's ability to move their phantom limb may affect their PLP.

**Methods**: In this research, we recruited five PLP patients to practice two motor tasks in multiple VR sessions over 6 weeks. In VR, patients "inhabit" a virtual body or avatar, and the movements of their intact limbs are mirrored in the avatar, providing them with the illusion that their limbs respond as if they were both intact and functional. The McGill Pain Questionnaire and 10-point VAS were used to collect pain ratings. We also conducted qualitative interviews after the entire study.

**Results**: We found that repeated exposure to our VR intervention led to reduced pain and improvements in anxiety, depression, and a sense of embodiment of the virtual body. Importantly, we also found that their ability to move their dysfunctional phantom limbs improved as quantified by shortened motor imagery time with the impaired limb.

**Discussion/Conclusions**: Although the limited sample size prevents us from performing a correlational analysis, our findings suggest that providing PLP patients with sensorimotor experience of the impaired limb in VR appears to offer long-term benefits for patients and that these benefits may be related to changes in patients control of the phantom limbs' movement.

### ORCID

Xin Tong http://orcid.org/0000-0002-8037-6301
Diane Gromala http://orcid.org/0000-0002-7737-9173
Kunlin Wei http://orcid.org/0000-0001-5098-3808

© 2021 The Author(s). Published with license by Taylor & Francis Group, LLC.
This is an Open Access article distributed under the terms of the Creative Commons Attribution License (http://creativecommons.org/licenses/by/4.0/), which permits unrestricted use, distribution, and reproduction in any medium, provided the original work is properly cited.

### What is the state of patient engagement among trainees conducting pain research in Canada? Results of a national web-based survey

Kyle Vader[a], Dawn P. Richards[b], Perri R. Tutelman[c], Catherine Paré[d], Alice Wagenaar-Tison[e], Kathryn A. Birnie[f], Christine T. Chambers[g], Kathleen Eubanks[e], Nader Ghasemlou[i], Janet Gunderson[j], Maria Hudspith[k], Therese Lane[l], Delane Linkiewich[l], and Jordan Miller[m]

[a]Queen's University, School of Rehabilitation Therapy, Kingston, Ontario, Canada; [b]Five02 Labs Inc., Toronto, Ontario, Canada; [c]Department of Psychology & Neuroscience, Dalhousie University, Halifax, Nova Scotia, Canada; [d]Department of Psychology, McGill University, Montréal, Québec, Canada; [e]Department of Anatomy, Université du Québec à Trois-Rivières, Trois-Rivières, Québec, Canada; [f]Department of Anesthesiology, Perioperative and Pain Medicine, University of Calgary, Calgary, Canada; [g]Departments of Pediatrics and Psychology & Neuroscience, Dalhousie University, Halifax, Nova Scotia; [h] Ehlers-Danlos Syndrome Canada & Chronic Pain Network, McMaster University, Hamilton, Ontario, Canada; [i]Departments of Anesthesiology and Biomedical & Molecular Sciences, Queen's University, Kingston, Ontario, Canada; [j]Saskatchewan Centre for Patient-Oriented Research & Chronic Pain Network, McMaster University, Hamilton, Ontario, Canada; [k]Pain BC, Vancouver, British Columbia; [l]Chronic Pain Network, McMaster University, Hamilton, Ontario, Canada; [m]Queen's University, School of Rehabilitation Therapy, Kingston, Ontario, Canada

**Introduction/Aim**: Although patient engagement (PE) is increasingly being recognized as an important component of pain research, little is known about PE among trainees in the field. The purpose of this study was to understand perspectives about and experiences with PE among trainees conducting pain research in Canada, to identify perceived barriers/facilitators, and to understand recommendations to improve its implementation. The research team includes patient partners, trainees, researchers, and others who facilitate PE in research.

**Methods**: A cross-sectional web-based survey (English and French) with trainees conducting pain research at Canadian academic institutions. Pain research trainees were invited to participate in the survey via targeted recruitment emails and social media posts.

**Results**: One hundred nineteen (N=119) online survey responses have been received; the majority of respondents were enrolled in a graduate program (n=75) and engaging in clinical pain research (n=77). Most respondents (n=95) indicated that PE is 'very' or 'extremely' important. Despite this, a minority of respondents (n=19) indicated that they 'often' or 'always' implement PE within their own research. The most common barrier was being 'unsure how to practically implement PE,' while 'seeing the value of PE' was the most commonly reported facilitator. Recommendations for improving the implementation of PE were diverse; however, many trainees commented on the importance of improved training opportunities.

**Discussion/Conclusions**: Despite viewing PE as an important component of pain research, few trainees regularly implement PE within their own research. Results highlight perceived barriers/facilitators to implementing PE and provide insight to inform the development of future training opportunities.

### ORCID

Kyle Vader http://orcid.org/0000-0002-0667-9726
Kathryn A. Birnie http://orcid.org/0000-0002-8223-8834
Christine T. Chambers http://orcid.org/0000-0002-7138-916X

© 2021 The Author(s). Published with license by Taylor & Francis Group, LLC.
This is an Open Access article distributed under the terms of the Creative Commons Attribution License (http://creativecommons.org/licenses/by/4.0/), which permits unrestricted use, distribution, and reproduction in any medium, provided the original work is properly cited.

### Experiences participating in a community-based exercise program tailored for adults living with chronic pain: an interpretive phenomenological analysis

Kyle Vader[a], Bayley Quinn[b], Thomas Abrams[c], Etienne J. Bisson[d], Darrien Cantelo[e], Tom Doulas[f], Schuyler Earl[g], Amy E. Latimer-Cheung[h], Jordan Miller[i], and Jennifer R. Tomasone[j]

[a]Queen's University, School of Rehabilitation Therapy, Kingston, Ontario, Canada; [b]Queen's University, School of Rehabilitation Therapy, Kingston, Ontario, Canada; [c] Queen's University, Department of Sociology, Kingston, Ontario, Canada; [d]Kingston Health Sciences Centre, Chronic Pain Clinic, Kingston, Ontario, Canada; [e]Queen's University, School of Rehabilitation Therapy, Kingston, Ontario, Canada; [f] Kingston Health Sciences Centre, Chronic Pain Clinic, Kingston, Ontario, Canada; [g] Queen's University, School of Kinesiology and Health Studies, Kingston, Ontario, Canada; [h] Queen's University, School of Kinesiology and Health Studies, Kingston, Ontario, Canada; [i] Jordan Miller, PhD, PT, Queen's University, School of Rehabilitation Therapy, Kingston, Ontario, Canada; [j] Queen's University, School of Kinesiology and Health Studies, Kingston, Ontario, Canada

**Introduction/Aim**: Regular participation in exercise can decrease pain severity and improve physical function among adults living with chronic pain. However, many adults living with chronic pain experience challenges when participating in exercise. In response, we developed an 8-week community-based exercise program that was tailored for adults living with chronic pain. The purpose of this study was to understand the experiences of adults living with chronic pain who participated in the tailored community-based exercise program.

**Methods**: We conducted semi-structured interviews with adults living with chronic pain who participated in the tailored community-based exercise program (N=7). Data were analyzed using interpretive phenomenological analysis.

**Results**: The majority of participants were women (n=5) who reported living with chronic pain for a median of 11 years. We constructed three superordinate themes (and subsequent subthemes) related to participant experiences in the tailored community-based exercise program: (1) the value of support (e.g. guidance, encouragement, and connections with peers); 2) navigating the challenges of exercise and chronic pain (e.g. fear of injury, pain flare ups, and adapting exercise); and 3) a changed outlook on living with chronic pain (e.g. improved confidence engaging in exercise, hope for the future, and changed views towards abilities). As a whole, participants described that engaging in the tailored community-based exercise program was a valuable experience that positively contributed to their chronic pain management.

**Discussion/Conclusions**: Results have implications for health care providers, researchers, and community-based organizations that are looking to develop, implement or evaluate community-based exercise programs for adults living with chronic pain.

### ORCID

Kyle Vader http://orcid.org/0000-0002-0667-9726
Amy E. Latimer-Cheung http://orcid.org/0000-0002-0442-6848
Jennifer R. Tomasone http://orcid.org/0000-0003-0446-9706

© 2021 The Author(s). Published with license by Taylor & Francis Group, LLC.
This is an Open Access article distributed under the terms of the Creative Commons Attribution License (http://creativecommons.org/licenses/by/4.0/), which permits unrestricted use, distribution, and reproduction in any medium, provided the original work is properly cited.

### Atogepant Significantly Reduces Mean Monthly Migraine Days in the Phase 3 Trial, ADVANCE

Jessica Ailani[a], Richard B. Lipton[b], Peter J. Goadsby[c], Hua Guo[d], Rosa Miceli[e], Lawrence Severt[f], Michelle Finnegan[g], and Joel M. Trugman[h]

[a]Georgetown University, Neurology, Washington, DC, USA; [b] Albert Einstein College of Medicine and Montefiore Headache Center, Neurology, Bronx, NY, USA; [c] NIHR-Wellcome Trust King's Clinical Research Facility, Neurology, London, England, UK; [d] AbbVie, Biostatistics, Madison, NJ, USA; [e] AbbVie, Clinical Development, Madison, NJ, USA; [f] AbbVie, Clinical Development, Biostatistics, Madison, NJ, USA; [g] AbbVie, Clinical Development, Biostatistics, Madison, NJ, USA; [h] JAbbVie, Clinical Development, Biostatistics, Madison, NJ, USA

**Introduction/Aim**: Atogepant is an oral, small molecule, CGRP receptor antagonist in development for the preventive treatment of migraine. We evaluated the efficacy, safety, and tolerability of atogepant over 12 weeks.

**Methods**: Phase 3 trial (NCT03777059). Adults with 4-14 migraine days per month were randomized 1:1:1:1 to once-daily atogepant 10mg, 30mg, 60mg, or placebo. The primary endpoint was a change from baseline in mean monthly migraine days (MMDs) across the 12-week treatment period. A key secondary endpoint was the proportion of participants with ≥50% reduction in their 3-month average of MMDs.

**Results**: The trial included 910 randomized participants (n=902 safety population, n=873 efficacy analysis population); >87% completed the double-blind treatment period across all groups. Mean change from baseline in MMDs: -3.69 atogepant-10mg, -3.86 atogepant-30mg, -4.20 atogepant-60mg versus -2.48 placebo (p<0.0001 all doses). The percentage of participants with ≥50% reduction in their 3-month average of MMDs: 56% atogepant-10mg, 59% atogepant-30mg, 61% atogepant-60mg versus 29% placebo (p<0.0001 all doses). AEs were reported by 52%-54% of atogepant-treated participants and 57% placebo. Most common AEs were constipation (7%-8% across doses vs 0.5% placebo) and nausea (4%-6% across doses vs 2% placebo); none were serious. Serious AEs were reported by 0.9% in atogepant 10mg and placebo; none were reported for 30mg or 60mg. Discontinuations due to AEs: 2%-4% across atogepant groups and 3% placebo. No hepatic safety issues were identified following daily dosing with atogepant.

**Discussion/Conclusions**: Atogepant provided statistically significant and clinically meaningful reductions in migraine days in the ADVANCE trial. Atogepant was safe and well-tolerated.

© 2021 The Author(s). Published with license by Taylor & Francis Group, LLC.
This is an Open Access article distributed under the terms of the Creative Commons Attribution License (http://creativecommons.org/licenses/by/4.0/), which permits unrestricted use, distribution, and reproduction in any medium, provided the original work is properly cited.

### Differential Expression of GluN2 NMDA Receptor Subunits in the Dorsal Horn of Male and Female Rats

Jennifer Armstrong, Santina Temi, Christopher Rudyk, Jeffery Landrigan, Christopher Dedek, and Michael Hildebrand

Carleton University, Neuroscience, Ottawa, Ontario, Canada

**Introduction/Aim**: N-methyl-D-aspartate receptors (NMDARs) are excitatory glutamatergic receptors located in the dorsal horn of the spinal cord. These tetrameric receptors consist of a combination of specific subunit variants; GluN2A, GluN2B, and GluN2D. Within the CNS, the function and structure of the receptor can be modified as a result of the combination of these subunits. However, despite the importance of NMDARs in pain signaling, there is little information in regard to the relative expression of GluN2 subunits in the superficial dorsal horn (SDH) in pain processing pathways. Surprisingly, the expression pattern of GluN2 in females remains completely unknown.

**Methods**: Immunohistochemical staining of male and female rats was used in conjunction with antigen retrieval, which removes cross-linkages that mask the receptor proteins at synaptic sites, as well as Tyrosine Signal Amplification which amplifies the fluorescence to aid in the quantification of subunit expression.

**Results**: In juvenile rats, we found GluN2B to be preferentially located in the superficial dorsal horn of female rats, while both GluN2B and GluN2D were preferentially located in the superficial dorsal horn in males. In addition, male rats demonstrated an increased expression of GluN2B in the medial SDH in comparison to the lateral SHD. We are actively comparing juvenile rats to adult rats to investigate developmental changes in GluN2 expression across the lifespan.

**Discussion/Conclusions**: Our findings of differential NMDAR subunit expression across dorsal horn laminae as well as sex has implications for both the understanding and treatment of pain in humans.

© 2021 The Author(s). Published with license by Taylor & Francis Group, LLC.
This is an Open Access article distributed under the terms of the Creative Commons Attribution License (http://creativecommons.org/licenses/by/4.0/), which permits unrestricted use, distribution, and reproduction in any medium, provided the original work is properly cited.

### COVID-19 impact and response by canadian pediatric pain clinics: a national survey of pediatric pain clinic medical directors

Tieghan Killackey[a], Myles Benayon[b], Melanie Noel[c], Katie Birnie[d], Manon Choiniere[e], Gabrielle Pagé, Lise Dassieu, Anaïs Lacasse, Chitra Lalloo[f], Patricia Poulin[g], Pablo Ingelmo[h], Samina Ali[i], Marco Battaglia[j], Fiona Campbell[k], Andrew Smith[l], Lauren Harris[o], Vina Mohabir[o], Isabel Jordan[m], Justina Marianayagam[n], and Jennifer Stinson[p] on behalf of COVIDChildPain Team

[a]The Hospital for Sick Children, Child Health Evaluative Sciences, Toronto, ON, Canada; [b]McMaster University, Medicine, Hamilton, Ontario, Canada; [c]University of Calgary, Department of Psychology, Calgary, Alberta, Canada; [d]University of Calgary, Department of Anesthesiology, Perioperative and Pain Medicine, Calgary, AB, Canada; [e]University of Montreal, Department of Anesthesiology, Montreal, QC, Canada; [f]The

Hospital for Sick Children, Toronto, ON, Canada; [g]University of Ottawa, Anesthesiology and Pain Medicine, Ottawa, Ontario, Canada; [h]Montreal Children's Hospital, Anesthesia and Chronic Pain Management, Montreal, QC, Canada; [i]University of Alberta, Medicine & Dentistry – Pediatrics, Edmonton, AB, Canada; [j]Division of Child and Youth Psychiatry, Toronto, ON, Canada; [k]The Hospital for Sick Children, Anesthesia and Pain Medicine, Toronto, Ontario, Canada; [l]Andrew Smith, MD, Centre for Addiction and Mental Health, Toronto, Ontario, Canada; [m]Squamish, British Columbia, Canada; [n]Northern Ontario School of Medicine, Sudbury, Ontario, Canada; [o]The Hospital for Sick Children, Child Health Evaluative Sciences, Toronto, Ontario, Canada; [p]The Hospital for Sick Children, Child Health Evaluative Sciences, Toronto, ON, Canada

**Introduction/Aim**: Due to the COVID-19 pandemic, youth are reporting greater amounts of pain complaints which, if left untreated, can lead to chronic pain (CP). This is further complicated by the discontinuation of in-person visits to pediatric pain clinics due to public health restrictions. Given 1 in 5 youth experienced CP before COVID-19, this crisis must be addressed. This study aimed to investigate the effect of the pandemic on Canada's 13 pediatric pain clinics.

**Methods**: Medical directors from 11 multi-disciplinary pediatric pain clinics and 2 specialized pediatric pain rehabilitation centres completed a cross-sectional survey assessing changes in care delivery and patient health during the COVID-19 pandemic.

**Results**: All (N=13/13, 100%) Canadian pediatric pain clinic directors completed the survey; collectively they provide care to 8268 patients annually. During the COVID-19 pandemic, clinics used video (N=13/13, 100%) and telephone (N=10/13, 77%) calling for virtual care. Although clinics (N=13/13, 100%) continued delivering physical, psychological, and/or education programs, some (N=5/13, 38%) reported longer waiting times. Furthermore, many (N=8/13, 62%) clinics stopped providing pain-relieving procedures (i.e. infusions). Clinics reported that patients experienced increased pain (N=4/13, 31%), medication use (N=5/13, 38%), depressive symptoms (N=6/13, 46%), and anxiety-related symptoms (N=5/13, 38%).

**Discussion/Conclusions**: COVID-19 has impacted pediatric pain clinics' ability to provide in-person care and forced the implementation of virtual care due to public health restrictions. This study highlights that some clinic directors have noted increased levels of pain, medication use, and mental health issues in their pediatric patients, emphasizing the need for nationwide health policy recommendations related to virtual care.

### ORCID

Tieghan Killackey http://orcid.org/0000-0003-2941-4058
Melanie Noel http://orcid.org/0000-0003-3752-8055
Manon Choiniere http://orcid.org/0000-0001-9593-8883
Lise Dassieu http://orcid.org/0000-0002-6311-3681
Anaïs Lacasse http://orcid.org/0000-0002-3992-5145
Samina Ali http://orcid.org/0000-0002-0595-364X
Jennifer Stinson http://orcid.org/0000-0002-9969-8052

© 2021 The Author(s). Published with license by Taylor & Francis Group, LLC.
This is an Open Access article distributed under the terms of the Creative Commons Attribution License (http://creativecommons.org/licenses/by/4.0/), which permits unrestricted use, distribution, and reproduction in any medium, provided the original work is properly cited.

### Ubrogepant is effective in the acute treatment of migraine with mild pain

Richard B. Lipton[a], David W. Dodick[b], Peter J. Goadsby[c], Rami Burstein[d], Aubrey M. Adams[e], Jeff Lai[f], Sung Yun Yu[g], Michelle Finnegan[g], and Joel M. Trugman[g]

[a]Albert Einstein College of Medicine and Montefiore Headache Center, Neurology, Bronx, NY, USA; [b]Mayo Clinic, Phoenix, AZ, USA; [c] NIHR-Wellcome Trust King's Clinical Research Facility, Neurology, London, England, UK; [d] Harvard Medical School, Beth Israel Deaconess Medical Center, Boston, MA, USA; [e] AbbVie, Medical Affairs, Irvine, CA, USA; [f] AbbVie, Biostatistics, Madison, NJ, USA; [g] AbbVie, Clinical Development, Madison, NJ, USA

**Introduction/Aim**: Ubrogepant, approved for the acute treatment of migraine, demonstrated efficacy in treating migraine with moderate/severe pain. Clinical guidance recommends treatment when pain is mild, a strategy studied here for ubrogepant.

**Methods**: Phase-3, open-label, 52-week trial (NCT02873221). Adults randomized 1:1:1 to usual care, or in blinded fashion to ubrogepant-50mg or ubrogepant-100mg, treated up to 8 migraine attacks every 4 weeks. Efficacy measures, collected only for ubrogepant, included 2-hour pain freedom and absence of migraine-associated symptoms.

**Results**: Participants' (n=808) mean age was 42 years; most were white and female. During the trial, 21,454 attacks were treated. Pain free rates were higher for the treatment of mild versus moderate/severe pain (average percentage across all treated attacks): 39% vs 19% ubrogepant-50mg (P<0.0001) and 43% vs. 21% ubrogepant-100mg (P<0.0001). Absence of photophobia was achieved in a higher proportion of attacks treated with mild pain versus moderate/severe pain: 55% both doses vs 34% both doses (P<0.0001). Absence of phonophobia was achieved in a higher proportion of attacks treated with mild pain versus moderate/severe pain: 64% vs 42% ubrogepant-50mg (P<0.0001) and 70% vs 45% ubrogepant-100mg (P<0.0001). Absence of nausea was achieved in a higher proportion of attacks treated with mild pain versus moderate/severe pain: 83% vs 67% ubrogepant-50mg (P<0.0001) and 82% vs 68% ubrogepant-100mg (P<0.0001). Overall, 10% of participants reported treatment-related adverse events; one was considered serious (exacerbation of sinus tachycardia).

**Discussion/Conclusions**: Treating migraine patients with ubrogepant when headaches are mild rather than moderate/severe increases the likelihood of rendering them free of pain and associated symptoms.

© 2021 The Author(s). Published with license by Taylor & Francis Group, LLC.
This is an Open Access article distributed under the terms of the Creative Commons Attribution License (http://creativecommons.org/licenses/by/4.0/), which permits unrestricted use, distribution, and reproduction in any medium, provided the original work is properly cited.

### Motivations and expectations for using cannabis products to treat pain in humans and dogs: a mixed methods study

Jean E. Wallace[a], Lori R. Kogan[b], Eloise C. J. Carr[c], and Peter W. Hellyer[d]

[a]Department of Sociology, University of Calgary, 2500 University Drive NW, Calgary, AB Canada T2N 1N4; [b]College of Veterinary Medicine and Biomedical Sciences, Colorado State University, Fort Collins, CO USA 80523; [c]Faculty of Nursing, University of Calgary, 2500 University Dr NW, Calgary, AB Canada T2N 1N4; [d]College of Veterinary Medicine and Biomedical Sciences, Colorado State University, Fort Collins, CO USA 80523

**Introduction/Aim**: More people are using cannabis to treat their own or their dog's chronic pain. This study identifies the reasons people use cannabis products to treat their own pain or their dog's pain and explores whether these products have fulfilled their expectations.

**Methods**: An online survey was used to collect quantitative and qualitative self-report data on respondents' perceptions, motivations and expectations about their or their dog's chronic pain and cannabis use. Quantitative responses from the two groups were compared using Chi-Square tests and qualitative data were analyzed using a thematic analysis.

**Results**: Human patients (N = 313) and dog owners (N = 204) reported similar motivations for using cannabis products, with more popular reasons being that cannabis products are natural, preferred over conventional medication, are believed to be a good treatment option for pain. Similar proportions of human patients and dog owners reported that the use of cannabis products fulfilled their expectations (86% vs. 82% respectively, $\chi^2$ (1, 200) = .59, p = .32). The qualitative data revealed that their expectations were met by reducing pain, increasing relaxation, and improving sleep, coping, functionality and overall well-being.

**Discussion/Conclusions**: The results suggest that people choose cannabis products because they are natural and a possible solution to managing chronic pain when conventional medicines have not been effective. Expectations regarding pain management are generally fulfilled by these products. It is recommended that both physicians and veterinarians work towards feeling more comfortable proactively broaching the subject of cannabis use with additional training and education.

### ORCID

Lori R. Kogan http://orcid.org/0000-0002-5816-9672

© 2021 The Author(s). Published with license by Taylor & Francis Group, LLC.
This is an Open Access article distributed under the terms of the Creative Commons Attribution License (http://creativecommons.org/licenses/by/4.0/), which permits unrestricted use, distribution, and reproduction in any medium, provided the original work is properly cited.

### A social media analysis of discourses on the opioid crisis

David M. Walton ,Phd[a], Anjali Shah ,BSc[a], Marudan Sivagurunathan ,PhD[b], and Richard Booth ,PhD[c]

[a]Department of Physical Therapy, Western University, London, Ontario, Canada; [b]Department of Health and Rehabilitation Sciences, Western University, London, Ontario, Canada; [c]School of Nursing, Western University, London, Ontario, Canada

**Introduction/Aim**: The competing public health crises of opioid use disorder and chronic pain continue to contribute to significant personal and social burden. We sought to explore the nature and volume of public discourses around the opioid crisis from the end of 2018

to end of 2019 (316 days) and again for 50 days through the 2nd quarter of 2020.

**Methods**: We did so by prospectively collecting ('scraping') posts on the popular social microblogging site www.twitter.com that included any of the hashtags: #opioid, #opioids, #opioidcrisis or #opioidepidemic. Metrics included posts per user, posts per day, other associated hashtags, and network connectivity and influence. Using simple counts and descriptives we identified the top users by volume and potential reach and the most frequently associated hashtags. Qualitative content analysis categorized the hashtags by topic. Time-based regression explored trends in volume by day, and spikes were explored to determine the events that led to increase. Social Network Analysis (SNA) identified users with the largest eigenvector centrality values, an indicator of network size and influence.

**Results**: A single user accounted for 5.1% of all tweet volume, but the SNA revealed more fruitful user targets for disseminating information, including the U.S. Centers for Disease Control and Prevention. Trend in volume showed significant decrease from 2018-2020, and the emergence of #COVID19 appeared to accelerate the decrease. Common code categories related to spirituality, addiction and mental health, pain, and recovery.

**Discussion/Conclusions**: Findings are expected to be useful for those wishing to monitor the discourses and identify targets for dissemination of public education.

### ORCID

Marudan Sivagurunathan , PhD http://orcid.org/0000-0002-3359-9553
Richard Booth, PhD http://orcid.org/0000-0002-0300-9954

© 2021 The Author(s). Published with license by Taylor & Francis Group, LLC.
This is an Open Access article distributed under the terms of the Creative Commons Attribution License (http://creativecommons.org/licenses/by/4.0/), which permits unrestricted use, distribution, and reproduction in any medium, provided the original work is properly cited.

### The effects of persisting with an ineffective pain coping strategy: a pilot study

Lindsey Yessick, Kathleen Hoy, and Tim Salomons

Queen's University, Psychology, Kingston, Ontario, Canada

**Introduction/Aim**: Those who perceive a behavioral solution for removing pain will expend more effort taking action (i.e., problem-focused strategy). In contrast, those who perceive low behavioral control might adapt a more passive, emotion-focused coping strategy. The strategy that is most effective in a given situation depends on the amount of behavioral control that is available, and the individual's ability to accurately gauge that control. As such, we would predict that persistence in an ineffective problem-focused coping strategy, would increase the aversive effects of nociceptive stimulation.

**Methods**: Twelve participants received 45 painful thermal stimuli and were offered two possible coping strategies: one problem-focused strategy (a puzzle that could result in complete cessation of pain stimulation if solved) and one emotion-focused strategy (comforting sounds that would run concurrent with subsequent stimuli but reduce distress). Participants were told they could switch to the emotion-focused strategy at any point. The problem-focused strategy was manipulated to be unsolvable, such that the emotion-focused strategy was the more adaptive option.

**Results**: Individuals that persisted with the ineffective active coping strategy ($n$=6) experienced greater pain intensity ($d = 0.25$) and unpleasantness ($d = 0.20$) than those who switched to the emotion-focused strategy ($n$=6). Similarly, individuals who persisted with the active strategy experienced a stronger increase in state anxiety ($d = 1.09$).

**Discussion/Conclusions**: Individuals who persisted with an ineffective strategy for coping with experimental pain in this pilot study experienced more pain and a greater increase in anxiety. Additional data are currently being collected.

© 2021 The Author(s). Published with license by Taylor & Francis Group, LLC.
This is an Open Access article distributed under the terms of the Creative Commons Attribution License (http://creativecommons.org/licenses/by/4.0/), which permits unrestricted use, distribution, and reproduction in any medium, provided the original work is properly cited.

### Understanding Parent Behaviours in Response to Child Acute Pain: Parent Heart Rate Variability, Anxiety, and Catastrophizing

Kaytlin Constantin, Rachel Moline, Lindsay Labonte, and C. Meghan McMurtry

University of Guelph, Department of Psychology, Guelph, ON, Canada

**Introduction/Aim**: Parent behaviours strongly predict children's acute pain response; less clear are the factors shaping parent behaviours. Heart rate variability (HRV) is considered a physiological correlate of current emotion regulatory efforts (moment-to-moment changes in HRV; "state HRV") and the capacity for emotion regulation (resting HRV; "trait HRV"). We examined how parent state HRV relates to parent behaviours before and during their child's acute pain, and trait HRV as a moderator between parental state anxiety, catastrophizing and behaviours.

**Methods**: Forty-five children between 7 and 12 years of age completed the cold pressor task (CPT) in the presence of a primary caregiver. Parents completed the State-Trait Anxiety Inventory-State and the Pain Catastrophizing Scale-Parents-State. Parent HRV was examined at 30-second epochs at rest ("trait HRV"), before (i.e., "state HRV-warm"), and during their child's CPT ("state HRV-cold"). HRV was calculated using a frequency-domain measure. Parent behaviours during the CPT were video recorded and coded as "coping-promoting" (CP) or "distress-promoting" (DP) using the Child-Adult Medical Procedure Interaction Scale-Revised.

**Results**: A small to moderate negative correlation was observed between state HRV-cold and CP behaviours during CPT. Parental trait HRV moderated the association between catastrophizing and DP behaviours.

**Discussion/Conclusions**: Parents with low state HRV during child pain engaged in greater CP behaviours, suggesting that modest decreases in parent HRV in the moment may be adaptive in the immediate pain context. In contrast, parents who generally have a low (vs. high) HRV may be at risk of engaging in DP behaviours when catastrophizing about their child's pain.

### ORCID

C. Meghan McMurtry http://orcid.org/0000-0002-3278-1169

© 2021 The Author(s). Published with license by Taylor & Francis Group, LLC.
This is an Open Access article distributed under the terms of the Creative Commons Attribution License (http://creativecommons.org/licenses/by/4.0/), which permits unrestricted use, distribution, and reproduction in any medium, provided the original work is properly cited.

### Pain acceptance and catastrophizing improve after Acceptance and Commitment Therapy and Cognitive Behavioural Therapy for chronic pain

Alison Crawford[a], Etienne J. Bisson[b], Rebecca McDermott[b], Katie Root-Clarke[b], Jennifer Bossio[b], Hannah Marchand[b], Katherine Fretz[a], and Dean A. Tripp[c]

[a]Queen's University, Department of Psychology, Kingston, ON, Canada; [b]Kingston Health Sciences Centre-Hotel Dieu Hospital site, Chronic Pain Clinic, Kingston, Ontario, Canada; [c]Queen's University, Departments of Psychology, Urology, & Anesthesiology, Kingston, ON, Canada

**Introduction/Aim**: Although cognitive behavioural therapy (CBT) and acceptance and commitment therapy (ACT) are two psychological interventions that are commonly used in chronic pain populations, their specific therapeutic mechanisms are poorly understood. One of the mechanisms of ACT is acceptance, whereas improving pain catastrophizing via a reduction in this behaviour is thought to be the primary mechanism of CBT. It is unclear whether these mechanisms are specific to ACT and CBT, respectively.

**Methods**: Patients were recruited from the Kingston Health Sciences Centre – Chronic Pain Clinic in Kingston, ON. Patients were randomized into ACT (n = 34) or CBT (n = 43). Both interventions consisted of weekly, two-hour group sessions over the course of eight weeks. Patients completed a baseline questionnaire before the intervention, then completed the same questionnaire at the end of the eight-week intervention. Two mixed model, repeated measures ANOVAs were performed to assess how acceptance and catastrophizing changed over time and across psychological interventions.

**Results**: Both acceptance and catastrophizing improved at the end of the eight weeks ($F(1, 75) = 6.53$, $p = .01$, $\eta_p^2 = .08$; $F(1, 75) = 7.18$, $p = .01$, $\eta_p^2 = .09$, respectively). Both acceptance and catastrophizing improved regardless of psychological intervention received, with last observation carried forward.

**Discussion/Conclusions**: Changes in chronic pain acceptance and catastrophizing occur in both ACT and CBT. These findings suggest that the mechanisms underlying success in either intervention are not specific to the type of psychological intervention. Healthcare providers can refer patients to either intervention to improve pain related cognitions.

© 2021 Crown. Published with license by Taylor & Francis Group, LLC.
This is an Open Access article distributed under the terms of the Creative Commons Attribution License (http://creativecommons.org/licenses/by/4.0/), which permits unrestricted use, distribution, and reproduction in any medium, provided the original work is properly cited.

### The optimal difficulty level in cognitive modulation

S. Desjardins, T. A. Vogel, and M. Roy

McGill University, Psychology, Montreal, Quebec, Canada

Distraction is commonly used as a coping strategy to reduce pain. In healthy participants, performing a demanding cognitive task is associated with significant pain reduction. While it is largely assumed that increasing the difficulty of the task is associated with greater analgesic effects, the importance of task difficulty in the cognitive modulation of pain has not been systematically investigated. Our study sought to investigate the effect of task difficulty on pain inhibition through cognitive effort.

**Introduction/Aim**: Assessing the role of task difficulty in the cognitive modulation of pain.

**Methods**: Twenty-seven healthy young adults (n = 27) were recruited to participate in the study. The experiment involved two calibration procedures in which we adjusted the level of pain and the levels of difficulty of the task to each participant followed by a main behavioural task. First, we performed a sensory calibration to adjust the pain level. Participants were presented with 28 thermal stimulations and asked to rate them. These ratings were fitted into a logistic regression model to determine what temperature corresponded to 150/200 on visual analogue scale, a level deemed moderately painful. Second, we performed a cognitive calibration to adjust task difficulty. Participants performed a difficult cognitive task, the 2-back, and a staircase method was used to identify three levels of difficulty adjusted to the participants' ability to perform on the task. The three levels were low, medium and high. Finally, participants performed the main behavioural task which combined pain and cognitive effort, both of which utilized the pain level and difficulty levels from the calibration procedures.

Participants performed the 2-back, varying at three levels of difficulty, while receiving painful stimulations and subsequently rating them.

**Results**: We performed a one-way repeated measures ANOVA to assess mean differences in pain ratings at the three different levels of difficulty. Mauchly's test of sphericity indicated that the assumption of sphericity had been violated, $\chi^2(2) = 0.500$, $p < .001$, therefore a Greenhouse-Geisser correction was used ($\mathcal{E} = 0.667$). We found a significant effect of task difficulty on pain ratings, $F(2, 26) = 7.03$, $p = 0.007$. Post-hoc comparisons using the Tukey HSD test showed that while pain ratings did not significantly differ between the low (M= 160, SD = 19.7) and high (M = 160, SD = 17.6) difficulty levels, they did significantly differ between the medium (M = 154, SD = 17.1) and low levels of difficulty as well as between the medium and high levels of difficulty.

**Discussion/Conclusions**: Overall, our findings suggest that adequate task difficulty is crucial in producing optimal pain reduction. Although engaging in a cognitive task can reduce pain, participants must be sufficiently engaged in the task for it to distract them away from pain. If the difficulty of the task exceeds the participants' ability, either being too low or too high, they will not be engaged toward the task, resulting in minimal pain reduction. Alternatively, if the difficulty is moderate, striking a perfect balance between the level of challenge of the task and the participant's ability, the participants will be optimally engaged in the task, yielding a larger reduction in pain. This study has large implications for pain management. Because chronic pain patients often struggle to complete daily cognitive tasks due to the persistence of their pain, the difficulty of these tasks should be adequately adjusted such that engagement in them can distract them away from their pain.

### ORCID

M. Roy http://orcid.org/0000-0002-3335-445X

© 2021 The Author(s). Published with license by Taylor & Francis Group, LLC.
This is an Open Access article distributed under the terms of the Creative Commons Attribution License (http://creativecommons.org/licenses/by/4.0/), which permits unrestricted use, distribution, and reproduction in any medium, provided the original work is properly cited.

### What role can pharmacists play in Opioid Stewardship? - Findings from a scoping review on opioid stewardship interventions by pharmacists.

Nyasha Gondora[a], Sarah G. Versteeg[a], Caitlin Carter[a], Lisa D. Bishop[b], Beth Sproule[c], Dana Turcotte[d], Katelyn Halpape[e], Michael A. Beazely[f], Shelita Dattani, Mona Kwong[a], Lisa Nissen[g], and Feng Chang[a]

[a]University of Waterloo, Pharmacy, Waterloo, Ontario, Canada; [b]University of Toronto, Pharmacy, Toronto, Ontario, Canada; [c]University of Manitoba, Pharmacy, Manitoba, Canada; [d]University of Saskatchewan, Pharmacy, Saskatchewan, Canada; [e]Memorial University, Pharmacy, Newfoundland and Labrador, Canada; [f]Lisa Nissen, Queensland University of Technology, Pharmacy, Brisbane, Australia; [g]University of British Columbia, Pharmacy, British Columbia, Canada

**Introduction/Aim**: Sixteen million individuals worldwide have or currently suffer from, opioid use disorder. In Canada, approximately 37% of opioid-dependent patients report obtaining opioids through prescriptions. Opioids remain the gold standard for the management of acute to severe pain that is related to advanced medical illness and the management of chronic pain. Due to increased awareness of the risks associated with opioid prescriptions, inter-professional efforts have been made to combat excessive prescribing or dosing of opioids. Pharmacists are in a strategic position to facilitate effective opioid stewardship as they play a central role on healthcare teams. This review was conducted to identify and summarize the published peer-reviewed evidence on the role of pharmacists in opioid stewardship and their impact on patient outcomes.

**Methods**: The scoping review was conducted according to the methodological framework proposed by Arksey and O'Malley, which was further modified by the Joanna Briggs Institute. Six databases were searched: PubMed (MEDLINE), Ovid Embase, Ovid International Pharmaceutical Abstracts, Scopus, Cochrane Library, and APA PsycInfo.

**Results**: Seventy-seven reports were identified from ten countries around the world, showcasing interventions across diverse populations and practice settings. Results showed education and medication adjustments were the most predominant activities and nearly all interventions improved at least one outcome measure. Other areas supported by evidence include community stakeholder education, policy and guideline setting and risk assessment.

**Discussion/Conclusions**: This scoping review provides valuable insight into the effective role pharmacists play in opioid stewardship. The findings informed barriers to implementation and gaps in the literature, providing direction for future research.

### ORCID

Lisa Nissen http://orcid.org/0000-0001-5826-4605
Feng Chang http://orcid.org/0000-0003-4661-6911

© 2021 The Author(s). Published with license by Taylor & Francis Group, LLC.
This is an Open Access article distributed under the terms of the Creative Commons Attribution License (http://creativecommons.org/licenses/by/4.0/), which permits unrestricted use, distribution, and reproduction in any medium, provided the original work is properly cited.

### Evaluating peripheral nerve integrity in pediatric subjects with neuropathic pain: Application of diffusion tensor imaging and sex-based differences

Scott Holmes[a], Anastasia Karapanagou[a], Steven Staffa[a], Ronald Borra[b], Alyssa Lebel[a], David Zurakowski[a], Christine Sieberg[a], Laura Simons[c], and David Borsook[a]

[a]Boston Children's Hospital, Department of Anesthesia, Critical Care and Pain Medicine, Boston, Massachusetts, USA; [b]University Medical Center Groningen, Department of Radiology and Nuclear Medicine, Netherlands; [c] Stanford University, Department of Anesthesia, perioperative and pain medicine, Stanford, California, USA

**Introduction/Aim**: Peripheral nerve injury can lead to neuropathic pain. There remains a poor understanding regarding how to quantify nerve fiber integrity after nerve injury and the extent to which contralateral nerve fibers are changed. The aim of this investigation was to evaluate the extent to which changes in peripheral nerve fibers can be quantified in pediatric subjects with neuropathic pain using ipsilateral and contralateral diffusion tensor imaging.

**Methods**: A total of 39 participants with unilateral ankle injury and neuropathic pain and 16 age/sex matched healthy controls were recruited. DTI was performed on the affected and unaffected legs at the branching of the sciatic nerve into the fibular and tibial divisions. The pain detection questionnaire was used to quantify self-reported pain symptoms.

**Results**: Higher levels of self-reported pain were found in the ankle injury cohort than healthy controls ($p < 0.001$). Relative to healthy controls, differences in nerve fiber integrity were found in the affected and unaffected limbs of ankle injury patients ($p < 0.005$).

Elevated diffusivity was found in all three nerve divisions when comparing DTI from the injured to non-injured ankles ($p < 0.05$). A main effect of sex was found wherein all three nerve divisions showed greater diffusivity for males than females ($p < 0.001$) in affected legs compared to healthy controls.

**Discussion/Conclusions**: Findings highlight the extent of ipsilateral and contralateral changes in the peripheral nervous system resulting from unilateral ankle injury in pediatric subjects with neuropathic pain. There was an observed effect of sex on nerve fiber integrity that should be accounted for in future research.

### ORCID

Scott Holmes http://orcid.org/0000-0002-5793-7983
Laura Simons http://orcid.org/0000-0002-3395-9483

© 2021 The Author(s). Published with license by Taylor & Francis Group, LLC.
This is an Open Access article distributed under the terms of the Creative Commons Attribution License (http://creativecommons.org/licenses/by/4.0/), which permits unrestricted use, distribution, and reproduction in any medium, provided the original work is properly cited.

### Impact of the COVID-19 Pandemic on Canadian Youth Living with Chronic Pain: A National Survey

Tieghan Killackey[a], Melanie Noel[b], Katie Birnie[c], Manon Choiniere[d], Chitra Lalloo[e], Patricia Poulin[f], Pablo Ingelmo[g], Samina Ali[h], Marco Battaglia[i], Fiona Campbell[j], Andrew Smith[k], Myles Benayon[l], Isabel Jordan[m], Justina Marianayagam[n], Lauren Harris[o], Vina Mohabir[o], and Jennifer Stinson[o] on behalf of COVIDChildPain Team

[a]The Hospital for Sick Children, Child Health Evaluative Sciences, Toronto, ON, Canada; [b]University of Calgary, Department of Psychology, Calgary, Alberta, Canada; [c] University of Calgary, Department of Anesthesiology, Perioperative and Pain Medicine, Calgary, AB, Canada; [d]University of Montreal, Department of Anesthesiology, Montreal, QC, Canada; [e] The Hospital for Sick Children, Toronto, ON, Canada; [f]University of Ottawa, Anesthesiology and Pain Medicine, Ottawa, Ontario, Canada; [g]Montreal Childrens Hospital, Anesthesia and Chronic Pain Management, Montreal, QC, Canada; [h] University of Alberta, Medicine & Dentistry – Pediatrics, Edmonton, AB, Canada; [i]Division of Child and Youth Psychiatry, Toronto, ON, Canada; [j]The Hospital for Sick Children, Anesthesia and Pain Medicine, Toronto, Ontario, Canada; [k] Centre for Addiction and Mental Health, Toronto, Ontario, Canada; [l] McMaster University, Medicine, Hamilton, Ontario, Canada; [m] Squamish, British Columbia, Canada; [n]Northern Ontario School of Medicine, Sudbury, Ontario, Canada; [o]The Hospital for Sick Children, Child Health Evaluative Sciences, Toronto, Ontario, Canada

**Introduction/Aim**: Youth are reporting increased pain during the COVID-19 pandemic, and without treatment this pain can become chronic (CP; pain lasting >3 months). CP in childhood can trigger mental health issues that carry forward into adulthood. Public health restrictions related to the pandemic have led pediatric pain clinics to halt in-person appointments, delaying access to care and dramatically disrupting daily routines critical for self-management.

**Methods**: The aim of this study was to evaluate the impact of the COVID-19 pandemic and associated restrictions on 200 Canadian youth living with CP. A cross-sectional survey of Canadians ages 8-18 was distributed electronically and explored impacts on pain levels, quality of life, mental health and more.

**Results**: Survey respondents (n=46) to date were predominantly white (74%) females (89%) with a mean age of 15.9 years and moderate levels of pain interference (56%); the majority attend a multidisciplinary pain management clinic (63%). Preliminary data shows that most youth experienced worsened (50%) or unchanged (39%) levels of pain during the pandemic. 50% of participants reported moderate to severe anxiety and 35% had scores indicating possible major depressive disorder. Youth generally preferred in-person appointments as virtual care presented challenges related to communication, physical assessment, and technological difficulties.

**Discussion/Conclusions**: Overall, preliminary results demonstrate that youth living with CP have been significantly impacted by the COVID-19 pandemic and associated restrictions. There is an urgent need for policy and tailored self-management interventions to address the increasing rates of pain and mental health concerns experienced by youth.

### ORCID

Tieghan Killackey http://orcid.org/0000-0003-2941-4058
Melanie Noel http://orcid.org/0000-0003-3752-8055
Katie Birnie http://orcid.org/0000-0002-8223-8834
Manon Choiniere http://orcid.org/0000-0001-9593-8883
Samina Ali http://orcid.org/0000-0002-0595-364X
Jennifer Stinson http://orcid.org/0000-0002-9969-8052

© 2021 The Author(s). Published with license by Taylor & Francis Group, LLC.
This is an Open Access article distributed under the terms of the Creative Commons Attribution License (http://creativecommons.org/licenses/by/4.0/), which permits unrestricted use, distribution, and reproduction in any medium, provided the original work is properly cited.

### Numeric anchoring appears to inhibit the relationship between depression scores and ratings of pain intensity in others.

Rebecca E. Lewinson and Joel Katz

York University, Department of Psychology, Toronto, ON, Canada

**Introduction**: Individuals who score higher on measures of pain catastrophizing (PC), anxiety, or depression tend to infer more intense pain in others. Anchoring effects occur when exposure to a numeric quantity biases subsequent judgments of other numeric quantities, and have been shown to influence how pain is inferred. It is currently unclear whether PC, anxiety, or depression scores are associated with pain ratings in the presence of a numerical anchor.

**Methods**: 390 participants completed measures of PC, anxiety, and depression and then read a vignette describing a patient with chronic pain before being randomly assigned to one of four anchoring groups. Groups 1 and 2 spun an 11-wedge number wheel (0-10) that was programmed to stop on a high number ('8'), or a low number ('2'). Group 3 spun a similar letter wheel (A-K) that was programmed to stop on 'C' or 'I', while Group 4 did not spin a wheel. Participants were asked to rate the patient's pain intensity on a 0-10 numeric rating scale.

**Results**: Regression analyses indicated that depression scores were related to pain intensity ratings among participants in Group 3, F(2,99)= 4.771, $p$=.003, $R^2$= .088, where a one-point increase in depression scores was associated with a .129-point increase in pain intensity. Anxiety and PC scores were not significantly related to pain ratings.

**Conclusions**: The presence of a numerical anchor may negate the effect that depression has on pain inferences, given that depression scores were associated with pain ratings only in the absence of a numerical anchor.

### ORCID

Rebecca E. Lewinson http://orcid.org/0000-0001-7157-2784
Joel Katz http://orcid.org/0000-0002-8686-447X

© 2021 The Author(s). Published with license by Taylor & Francis Group, LLC.
This is an Open Access article distributed under the terms of the Creative Commons Attribution License (http://creativecommons.org/licenses/by/4.0/), which permits unrestricted use, distribution, and reproduction in any medium, provided the original work is properly cited.

### School anxiety in emerging adult college students with chronic pain

Emily A. Beckmann and Kristen E. Jastrowski Mano

University of Cincinnati, Department of Psychology, Cincinnati, Ohio, USA

**Introduction/Aim**: Anxiety is a common comorbidity among youth with chronic pain. School is a context in which youth often experience anxiety, which plays a central role in maintaining school impairment. Despite consistent evidence of elevated school anxiety and impairment in children and adolescents with chronic pain, neither has been investigated in emerging adults, despite heightened stress and anxiety being commonly reported in this population. The aim of this study was to determine whether emerging adult college students with chronic pain report worse school anxiety compared to healthy peers.

**Methods**: Emerging adult college students ($N$ = 740, $M_{age}$ = 19.8, 61.8% female, 75% Caucasian, 27.8% with CP) completed the School Anxiety Inventory – College Version (SAI-CV) and a demographic and pain history questionnaire.

**Results**: Participants with chronic pain reported significantly more school anxiety compared to healthy peers ($t(738)$ = 2.96, $p$ < .01). Specifically, those with chronic pain reported more behavioral ($t(738)$ = 3.32, $p$ = .001) and physiological ($t(738)$ = 3.12, $p$ < .01) anxiety symptoms, but not cognitive symptoms ($p$ > .05). Participants with chronic pain reported significantly more anxiety in the context of academic failure ($t(738)$ = 3.30, $p$ = .001), social evaluation ($t(738)$ = 2.31, $p$ < .05), and peer aggression ($t(738)$ = 2.28, $p$ < .05) than healthy peers.

**Discussion/Conclusions**: College students with chronic pain experience significantly more school anxiety compared to healthy peers. The impact of school anxiety on school-related functional impairment in college students with CP necessitates further investigation.

© 2021 The Author(s). Published with license by Taylor & Francis Group, LLC.
This is an Open Access article distributed under the terms of the Creative Commons Attribution License (http://creativecommons.org/licenses/by/4.0/), which permits unrestricted use, distribution, and reproduction in any medium, provided the original work is properly cited.

### Classification of qualitative fieldnotes collected during quantitative sensory testing: A step towards the development of a new mixed methods approach in pain research

Martine Bordeleau[a], Michel Prud'Homme[b], Lynn R. Gauthier[c], Catherine Estelle Ferland[d], Miroslav Backonja[e], Jan Vollert[f], Serge Marchand[g], Philip Leslie Jackson[h], Léo Cantin[i], and Guillaume Léonard[j]

[a]School of Rehabilitation, Faculty of Medicine and Health Sciences, Université de Sherbrooke, Quebec, Canada; Research Center on Aging, CIUSSS de l'Estrie-CHUS, Quebec, Canada; [b]Centre de recherche du CHU de Québec – Université Laval, Divisions of neurosciences and neurosurgery, Quebec City, Quebec, Canada; [c]McGill University, Department of Anesthesia, Montreal, Canada; Integrated Program in Neuroscience, McGill University, Canada; Shriners Hospitals for Children-Canada, Montreal, Canada; [d]University of Wisconsin Department of Neurology, Madison, Wisconsin, United States; University of Washington, Department of Neurology, Seattle, Washington, United States; [e]Imperial College London, Pain Research, Department of Surgery and Cancer, London, United Kingdom; Center of Biomedicine and Medical Technology Mannheim CBTM, Department of Neurophysiology, Medical Faculty Mannheim, Heidelberg University, Mannheim, Germany; [f]Sherbrooke University Hospital Research Center (CRCHUS), Sherbrooke, Canada; University of Sherbrooke, Department of Surgery, Faculty of Medicine, Sherbrooke, Canada; [g]Center for Interdisciplinary Research in Rehabilitation and Social Integration, Quebec City, Quebec, Canada; CERVO Brain Research Center, Québec City, Québec, Canada; Laval University, School of Psychology, Quebec City, Quebec, Canada; [h]Centre de recherche du CHU de Québec – Université Laval, Divisions of neurosciences and neurosurgery, Quebec City, Quebec, Canada; [i]Virginia Commonwealth University, Department of Physical Therapy, Richmond, Virginia, United States; [j]School of Rehabilitation, Faculty of Medicine and Health Sciences, Université de Sherbrooke, Quebec, Canada; Research Center on Aging, CIUSSS de l'Estrie-CHUS, Quebec, Canada

**Introduction/Aim**: Quantitative sensory testing (QST) is a standardized method to assess somatosensory function. The collection of qualitative information, during the QST procedure, could be an interesting way to facilitate the characterization of altered sensory perception and the identification of different pain phenotypes. The aims of this study were 1) to classify qualitative fieldnotes of sensory abnormalities collected during an independent QST study, and 2) to generate a qualitative interview guide that could be included in the traditional QST procedure as a step towards the implementation of a mixed methods approach.

**Methods**: QST data were collected from 48 chronic neuropathic pain patients treated with spinal cord stimulation (SCS). Three body areas, with or without SCS, were tested: the painful limb targeted by SCS, the contralateral area, and the ipsilateral upper limb. After each trial of each QST modality, patients were encouraged to report any sensory abnormalities they could identify with a pain quality scale or using their own words.

**Results**: Qualitative self-reported sensory abnormalities were dichotomized into two groups: altered sensory intensities and altered sensory perceptions. Altered sensory intensities were classified as sensory loss or sensory gain subgroups. Altered sensory perceptions were classified as paresthesia and dysesthesia subgroups Overall, 630 qualitative fieldnotes of altered sensations were collected: 385 on the painful limb, 173 at the contralateral area, and 72 at the ipsilateral upper limb. Based on these qualitative data, we propose a standardized method to collect qualitative data involving 9 open- and close-ended questions and 21 codes.

**Discussion/Conclusions**: Our findings have highlighted the value of qualitative sensory evaluation during QST and constitute an important milestone in the development of a mixed methods protocol in phenotyping research.

### ORCID

Jan Vollert http://orcid.org/0000-0003-0733-5201

© 2021 The Author(s). Published with license by Taylor & Francis Group, LLC.
This is an Open Access article distributed under the terms of the Creative Commons Attribution License (http://creativecommons.org/licenses/by/4.0/), which permits unrestricted use, distribution, and reproduction in any medium, provided the original work is properly cited.

### "Every furrow of her brow makes me want to stop": Qualitative analysis of mothers' experiences with their child's arthritis treatments

Yvonne N. Brandelli[a], Perri R. Tutelman[a], Christine T. Chambers[b], Jennifer A. Parker, Adam M. Huber[c], Jennifer N. Stinson[d], Emma S. Cameron[e], and Jennifer Wilson[f]

[a]Dalhousie University, Psychology and Neuroscience, Halifax, Nova Scotia, Canada; [b]Dalhousie University & IWK Health Centre, Psychology and Neuroscience & Pediatrics, Halifax, Nova Scotia, Canada; [c]Dalhousie University & IWK Health Centre, Pediatrics & Rheumatology, Halifax, Nova Scotia, Canada; [d]Lawrence S. Bloomberg Faculty of Nursing, University of Toronto and Research Institute at The Hospital for Sick Children, Toronto, Ontario, Canada; [e]Dalhousie University, School of Health and Human Performance, Halifax, Nova Scotia, Canada; [f]Cassie and Friends, Vancouver, British Columbia, Canada

**Introduction/Aim**: Juvenile Idiopathic Arthritis (JIA) is a chronic and often painful condition affecting 10,000 Canadian children. While treatments help to manage JIA, many involve procedural pain (e.g., self-injections) or side effects (e.g., abdominal pain). Parents play a key role in the administration of and adherence to JIA treatments, but little is known about their experiences in this role. The objective of this study was to explore parents' lived experiences with their child's JIA treatments.

**Methods**: In partnership with Cassie & Friends, 10 mothers of children with JIA (60% female, $M_{age}$=11.83, range=4-16 years) took part in semi-structured interviews, sharing their experiences with their child's JIA treatments. Data were analyzed using interpretative phenomenological analysis.

**Results**: Four superordinate themes were generated: (1) treatments increased mothers' advocacy and caregiver burden, consequently shifting their roles within the family; (2) treatments altered mothers' relationships in both positive (e.g., increased support) and negative (e.g., fears of harming their child) ways; (3) treatments, and their child's reactions to treatments, elicited various emotional responses in mothers (e.g., frustration, grief) which affected their well-being; and (4) treatments were a source of internal conflict for mothers (i.e., deciding how to act based on treatments benefits and harms).

**Discussion/Conclusions**: Mothers play a key role in their child's JIA treatments, and these experiences trickle down to affect them on a multitude of levels. Results highlight the value of supporting parents through these complex treatment regimens and incorporating their experiences in treatment decisions to ensure optimal outcomes for children with JIA and their families.

### ORCID

Christine T. Chambers http://orcid.org/0000-0002-7138-916X
Jennifer A. Parker http://orcid.org/0000-0001-9900-4703
Jennifer N. Stinson http://orcid.org/0000-0002-9969-8052

© 2021 The Author(s). Published with license by Taylor & Francis Group, LLC.
This is an Open Access article distributed under the terms of the Creative Commons Attribution License (http://creativecommons.org/licenses/by/4.0/), which permits unrestricted use, distribution, and reproduction in any medium, provided the original work is properly cited.

### Neuroethical issues in adopting brain imaging for personalized chronic pain management: Attitudes and demographics of people with chronic pain

Karen Deborah Davis[a], Monica de Oliveira[b], Ariana Besik[a], and Daniel Buchman[a]

[a]University Health Network, Krembil Research Institute, Toronto, Ontario, Canada; [b]Monica de Oliveira, University of Toronto, Toronto, Ontario, Canada

**Introduction/Aim**: The subjective nature of pain places a burden on individuals to convince others they have pain. Brain imaging can potentially inform pain management but raises neuroethical questions. We aimed to assess attitudes of people living with chronic pain across Canada regarding neuroethical issues in using brain imaging to detect and treat chronic pain.

**Methods**: A 41-item questionnaire assessed attitudes toward new brain imaging technologies, brain data privacy, stigma, treatment, objective representations of pain, and dismissing pain self-reports.

**Results**: We surveyed 349 people living with chronic pain. Most (>90%) were open to using brain imaging for diagnostics, prediction, and therapeutic decision-making. Although >50% of participants felt a brain scan would give them more confidence in diagnosis and treatment plans and that healthcare providers would be more likely to believe they had chronic pain, they worried that brain scans could be used to dismiss their pain self-report. Most (80%) participants believed there are policies and procedures to protect their brain data, but 40% were concerned about privacy and brain scan use against them by employers/insurers. Although >50% of participants felt a brain scan could represent their pain and suffering, 80% disagreed their pain is only real if seen in a brain scan. Demographics (education, age, province) influenced many of these findings.

**Discussion/Conclusions**: Participants recognize potential benefits of brain imaging but are concerned about data security and dismissal of their self-reported pain. These data align with pain experts' recommendations to develop brain imaging as an adjunct but not to replace pain self-reports.

### ORCID

Karen Deborah Davis http://orcid.org/0000-0003-1879-0090
Daniel Buchman http://orcid.org/0000-0001-8944-6647

© 2021 The Author(s). Published with license by Taylor & Francis Group, LLC.
This is an Open Access article distributed under the terms of the Creative Commons Attribution License (http://creativecommons.org/licenses/by/4.0/), which permits unrestricted use, distribution, and reproduction in any medium, provided the original work is properly cited.

### #ItDoesntHaveToHurt: Mapping the evolution of a hashtag to improve children's pain awareness on Twitter

Justine Dol[a], Jennifer A. Parker[a], and Christine T. Chambers[b]

[a]IWK Health Centre, Centre for Pediatric Pain Research, Halifax, NS, Canada; [b]Dalhousie University, Departments of Psychology & Neuroscience and Pediatrics, Halifax, NS, Canada

**Introduction/Aim**: Hashtags are used on social media platforms to generate and curate content on various topics. The #ItDoesntHaveToHurt hashtag was first used on Twitter related to children's pain management in November 2013 with the launch of a YouTube video for parents to manage their children's needle pain. Its use was continued with a CIHR-funded #ItDoesntHaveToHurt initiative that began in September 2015 and has since been adopted by the NCE-funded knowledge mobilization network, Solutions for Kids in Pain (SKIP). The aim of this project is to map the evolution of the #ItDoesntHaveToHurt hashtag on Twitter over a seven-year period.

**Methods**: All tweets from November 1, 2013 to October 31, 2020 using the #ItDoesntHaveToHurt hashtag were extracted from historical Twitter data. Tweets were summed by month, separating the number of original tweets, replies, and re-tweets.

**Results**: Overall, 67,964 tweets occurred over the seven years (84 months), 10,763 (15.8%) were original tweets, 46,528 (68.5%) were re-tweets, and 10,673 (15.7%) were replies. There were three peak months that corresponded with #ItDoesntHaveToHurt Twitter chats (March 2016, September 2016, and November 2017). Two other peak months corresponded with the #ItDoesntHaveToHurt launch (September 2015) and SKIP launch (April 2019).

**Discussion/Conclusions**: The #ItDoesntHaveToHurt hashtag mobilized evidence-based pediatric pain management through continued use of the hashtag on Twitter, reaching a variety of stakeholders, including patients, caregivers, health professionals, and administrators and policy makers. Hashtags can be used as an effective strategy to drive sharing of evidence-based content and amplify messages.

### ORCID

Justine Dol http://orcid.org/0000-0002-8928-7647
Jennifer A. Parker http://orcid.org/0000-0001-9900-4703
Christine T. Chambers http://orcid.org/0000-0002-7138-916X

© 2021 The Author(s). Published with license by Taylor & Francis Group, LLC.
This is an Open Access article distributed under the terms of the Creative Commons Attribution License (http://creativecommons.org/licenses/by/4.0/), which permits unrestricted use, distribution, and reproduction in any medium, provided the original work is properly cited.

### Does Technology Improve Pain Reporting Compliance and Satisfaction in Adults with Chronic Pain?

Landon Montag[a], Tracy Cupido[b], Scott Duggan[c], Christopher Haley[d], and Etienne J. Bisson[e]

[a]Queen's University, Centre for Neuroscience Studies, Kingston, Ontario, Canada; [b]Kingston Health Sciences Centre, Department of Anesthesiology and Perioperative Medicine, Kingston, Ontario, Canada; [c]Kingston Health Sciences Centre & Queen's University, Departments of Anesthesiology and Perioperative Medicine, and Biomedical and Molecular Sciences, School of Medicine, Kingston, Ontario, Canada; [d]Kingston Health Sciences Centre & Queen's University, Department of Anesthesiology and Perioperative Medicine, Kingston, Ontario, Canada; [e]Kingston Health Sciences Centre & Queen's University, Department of Anesthesiology and Perioperative Medicine, and the School of Rehabilitation Therapy, Kingston, Ontario, Canada

**Introduction/Aim**: Despite technology advances in medicine, most clinicians use unreliable paper pain diaries to assess pain treatment responses. This study aimed to determine patient responsiveness and the validity of electronic pain diaries (e-diary) compared to paper pain diaries (p-diary) following treatment.

**Methods**: Patients scheduled for a medical intervention at the Kingston Health Sciences Centre Chronic Pain Clinic were recruited and block randomized into either the p-diary or e-diary group before their treatment. Patients reported their pain intensity daily for 4 weeks post-treatment. Subsequently, compliance, satisfaction, and global impression of change were collected. Pain interference, quality of life and depression measures were also collected before and after the 4 week monitoring. Comparative analyses were performed and correlations were completed to test the concurrent and convergent validity.

**Results**: The e-diary group (n=27) had a median compliance of 75% (IQR=39-86%) and a median satisfaction of 80% (IQR=65-100%), whereas the p-diary group (n=18) was 98% (IQR=0-100%) and 80% (IQR=50-100%), respectively. Differences were not significant between groups (p≥0.05), but 78% would prefer using an e-diary. Stronger correlations were found between e-diary pain scores and outcome measures at follow-up compared to p-diary pain scores, suggesting greater concurrent and convergent validity for e-diaries.

**Discussion/Conclusions**: Since e-diaries had good patient responsiveness and greater accessibility, validity and accuracy of pain score data compared to p-diaries, their implementation in clinical settings would be beneficial.

© 2021 The Author(s). Published with license by Taylor & Francis Group, LLC.
This is an Open Access article distributed under the terms of the Creative Commons Attribution License (http://creativecommons.org/licenses/by/4.0/), which permits unrestricted use, distribution, and reproduction in any medium, provided the original work is properly cited.

### Psychosocial predictors of depressive symptoms in adolescents diagnosed with Inflammatory Bowel Disease (IBD)

Julia Moreau[a], Dean A. Tripp[b], Alison Crawford[a], Valentina Mihajlovic[c], and Katherine M. Fretz[d]

[a]Queen's University, Department of Psychology, Kingston, ON, Canada; [b]Queen's University, Departments of Psychology, Urology, & Anesthesiology, Kingston, ON, Canada; [c]Queen's University, Psychology, Kingston, Ontario, Canada; [d]Department of Psychology, Queen's University, Kingston, ON, Canada

**Introduction/Aim**: Inflammatory Bowel Disease (IBD) is a debilitating chronic medical condition characterized by severe inflammation of the gastrointestinal tract. Affecting millions of people worldwide, IBD is frequently diagnosed during adolescence. IBD is widely associated with psychosocial deterioration among young patients, especially depression. However, there are gaps of knowledge when considering factors that promote or reduce depressive symptoms. Specifically, perceived social support, personal self-esteem, and relational self-esteem are related to the relationship between perceived stress and depressive symptoms in adolescents, but the unique associations require clarification.

**Methods**: Adolescents, ages 16 through to 21 years old, participated in an online self-report survey assessing perceived stress, perceived social support, measures of self-esteem, and depressive symptoms via the online platform, Qualtrics. Cross-sectional parallel and serial mediation models examined the associations between perceived stress (predictor variable) and depressive symptoms (outcome variable), through perceived social support (mediator one), and a parallel and serial mediation with personal self-esteem (mediator two) and relational self-esteem (mediator three).

**Results**: Personal, but not relational, self-esteem significantly mediated the relationship between perceived stress and depressive symptoms. Additionally, results suggested 59% of the adolescents should be assessed for a DSM-5 diagnosis of depression.

**Discussion/Conclusions**: These novel results highlight personal self-esteem as a pivotal tenet to be considered in the treatment of alarmingly severe depressive symptoms among adolescent patients with IBD. Healthcare providers should be prepared to routinely assess, recognize, and promote efficacious treatment of adolescent depression.

© 2021 The Author(s). Published with license by Taylor & Francis Group, LLC.
This is an Open Access article distributed under the terms of the Creative Commons Attribution License (http://creativecommons.org/licenses/by/4.0/), which permits unrestricted use, distribution, and reproduction in any medium, provided the original work is properly cited.

### Astrocytic Gap Junctional Proteins are Necessary for Long-Term Potentiation (LTP) within the Spinal Cord Dorsal Horn

David Rodriguez and Robert Bonin

University of Toronto, Pharmaceutical Sciences, Toronto, Ontario, Canada

**Introduction/Aim**: The long-term potentiation (LTP) of synaptic connections between primary afferent fibers and projection neurons of the superficial dorsal horn has been closely associated with the development of hyperalgesia and allodynia. Recent investigations have demonstrated the importance of glial cells in basal neurotransmission and synaptic plasticity within the brain. Astrocytes, in particular, produce gliotransmitters that support the energetic/metabolic needs of active neurons. These cells have the ability to sense neuronal firing and they communicate with one another via gap junctional proteins (i.e. connexins and pannexins). In this study, we test the hypothesis that gap junctional proteins are necessary for proper astrocytic function and the development of spinal LTP.

**Methods**: Spinal cord explants are produced from adult C57BL/6J mice. Electrophysiological recordings of postsynaptic field potentials (fPSPs) in lamina I neurons are made using electrical stimulation of dorsal roots. After achieving a stable baseline, LTP is induced via low frequency stimulation (LFS).

**Results**: In the control group, LFS induces the potentiation of nociceptive pathways to 125% Baseline. Administration of 40μM Mefloquine (connexin blocker) occludes LTP and results in depression of synaptic strength (70% Baseline). Similarly, administration of 1mM Probenecid (pannexin blocker) prevents the induction of dorsal horn LTP (90% Baseline). Concomitant administration of L-lactate, an astrocyte-derived energy substrate, partially reverses the effect of Mefloquine and Probenecid on synaptic strength.

**Discussion/Conclusions**: Our results indicate that pharmacological blockade of gap junctional proteins affects the release of gliotransmitters, which are necessary for the expression of spinal LTP. Future experiments will focus on determining whether supplementation with other astrocyte-derived molecules can further restore synaptic potentiation.

© 2021 The Author(s). Published with license by Taylor & Francis Group, LLC.
This is an Open Access article distributed under the terms of the Creative Commons Attribution License (http://creativecommons.org/licenses/by/4.0/), which permits unrestricted use, distribution, and reproduction in any medium, provided the original work is properly cited.

### The impact of COVID-19 on youth with chronic pain: A thematic analysis

Tatiana Lund[a], Alex Neville[a], Sabine Soltani[a], Kathryn A. Birnie[a], Abbie Jordan[b], and Melanie Noel[a]

[a]Department of Psychology, University of Calgary, Calgary, Alberta, Canada; [b]Department of Psychology, University of Bath, Bath, Somerset, United Kingdom

**Introduction/Aim**: COVID-19 has had widespread implications on mental health worldwide. Several studies have noted a particular impact on the mental health of youth. However, no studies to date have examined the impact of COVID-19 on the mental health and identity of youth with chronic pain. The current study sought to fill this gap.

**Methods**: Twenty youth with chronic pain were interviewed separately by trained interviewers about their experience with chronic pain during the COVID-19 pandemic. Interview questions centered on mental health and pain. Interviews occurred between the months of June-August 2020 and were contextualized within the local and global socio-political context and timeline of COVID-19 policies/events. A thematic analytic approach was taken to the data.

**Results**: Preliminary results indicated an impact for youth in social, mental health and identity realms. Some youth described feeling increased stress and social isolation in response to restrictions, while others described feeling a sense of relief from fewer social demands. For some, pain decreased due to the comfort of staying at home, whereas others noted no change or an increase in pain. Youth described experiencing disruptions to their developmental trajectory, uncertainty about the future, and that their sense of purpose was put on hold.

**Discussion/Conclusions**: Youth with chronic pain report an impact of COVID-19 on several domains of life. As rates of COVID-19 continue to increase, qualitative data is necessary to understand the complex and ongoing impact of the pandemic on youth with chronic pain. Future research is warranted to track the impact of COVID-19 longitudinally in this population.

### ORCID

Kathryn A. Birnie http://orcid.org/0000-0002-8223-8834
Melanie Noel http://orcid.org/0000-0003-3752-8055

**CONTACT** Tatiana Lund tclund@ucalgary.ca

© 2021 The Author(s). Published with license by Taylor & Francis Group, LLC.
This is an Open Access article distributed under the terms of the Creative Commons Attribution License (http://creativecommons.org/licenses/by/4.0/), which permits unrestricted use, distribution, and reproduction in any medium, provided the original work is properly cited.

### How do Motor Vehicles Accident (MVA) victims fair in an interdisciplinary pain management program, compared to Non-MVA pain patients?

Angela Mailis[a] and S. Fatima Lakha[b]

[a]Pain and Wellness Centre, Vaughan Ontario; Dept. of Medicine, Division of Physical Medicine, University of Toronto; and Toronto Rehab Institute/ University Health Network, Toronto, Ontario, Canada; [b]Institute of Medical Science, University of Toronto, Toronto, Ontario, Canada

**Introduction/Aim**: To compare demographics and outcomes of MVA and non-MVA patients attending a community-based academic interdisciplinary pain clinic.

**Methods**: This retrospective study was conducted on 121 consecutive patients, screened for motivation and commitment and admitted to an intense pain program during 2016 –2017 (MVA n=31 and non-MVA n=90). Data collected included: a) demographics; b) pain characteristics; and c) emotional and functional status obtained by validated instruments. Means of pre-and post-program variables were compared to assess effectiveness of both groups' "journey".

**Results**: No difference was found in age, employment, or pain ratings. However, statistically significant differences at 0.05 level existed as follows: The MVA group was much more likely to have a lawyer (66.5 vs 13.3 for the non-MVA group); had greater female prevalence (1:2.9 MVA and 1:1.7 Non-MVA); more representation of Canadian born (77% MVA vs 64% non-MVA); much shorter duration of pain complaints (average pain duration 41±38 months (MVA) vs 80±48 (Non-MVA); and higher scores in Kinesiophobia, GAD (anxiety) and CESD (depression). However, outcome data indicated substantial and similar improvement in both groups- MVA vs Non-MVA: 60 vs 57% in CESD; 48 vs 51% in self-efficacy; 39 vs 46% in pain catastrophizing; 43 vs 41% in GAD; 29 vs 32% in BPI pain severity; 35 vs 39% in BPI interference score; and 14 vs 14% Kinesiophobia.

**Discussion/Conclusions**: Despite the presence of litigation that adversely affects outcomes for patients involved in car accidents, selected highly motivated MVA patients can do as well as non-MVA victims in pain management programs.

### ORCID

Angela Mailis http://orcid.org/0000-0001-5662-8989
S. Fatima Lakha http://orcid.org/0000-0002-3325-7553

**CONTACT** Angela Mailis angela.mailis@uhn.ca

© 2021 The Author(s). Published with license by Taylor & Francis Group, LLC.
This is an Open Access article distributed under the terms of the Creative Commons Attribution License (http://creativecommons.org/licenses/by/4.0/), which permits unrestricted use, distribution, and reproduction in any medium, provided the original work is properly cited.

### Pediatric chronic pain in the midst of the COVID-19 pandemic: Parent Perspectives

Alexandra Neville[a], Tatiana Lund[a], Sabine Soltani[a], Kathryn A. Birnie[b], Abbie Jordan[c], and Melanie Noel[a]

[a]Psychology Department, University of Calgary, Calgary, AB, Canada; [b]Department of Anesthesiology, Perioperative and Pain Medicine, University of Calgary, Calgary, AB, Canada; [c]Department of Psychology and Centre for Pain Research, University of Bath, Bath, UK

**Introduction/Aim**: The coronavirus 2019 (COVID-19) pandemic has impacted daily lives worldwide. Youth with chronic pain and their families now experience additional barriers and challenges. This study provided a detailed qualitative examination of experiences of the COVID-19 pandemic from the perspectives of parents of youth with chronic pain.

**Methods**: Narratives were elicited through individual interviews with twenty parents of youth with chronic pain (aged 10-18 years), recruited from a tertiary level pediatric chronic pain program. Interviews focused on exploring parents' experiences of the COVID-19 pandemic and impacts on their mental health, family, and their child's pain. Narratives were analyzed using inductive reflexive thematic analysis.

**Results**: Preliminary findings show that parents reported rapid and evolving changes to their daily lives due to the COVID-19 pandemic. Parents expressed uncertainty, grief for both the past and the future, and worry about the long-term impact these changes to daily life would have on their child. Parents described great resilience in their own and their child's ability to adapt to the challenges of living during the COVID-19 pandemic. They also described silver linings of this experience, including newfound appreciation for their life circumstances, stronger family connection, and deeper reflection on their values.

**Discussion/Conclusions**: As a result of the COVID-19 pandemic, youth with chronic pain and their parents are experiencing shifts in daily life, how they view their future, and how they cope in the present. Understanding parents' and youths' experiences of the COVID-19 pandemic is essential for supporting youth and families now and into the future.

### ORCID

Kathryn A. Birnie http://orcid.org/0000-0002-8223-8834
Melanie Noel http://orcid.org/0000-0003-3752-8055

**CONTACT** Alexandra Neville alexandra.neville@ucalgary.ca

© 2021 The Author(s). Published with license by Taylor & Francis Group, LLC.
This is an Open Access article distributed under the terms of the Creative Commons Attribution License (http://creativecommons.org/licenses/by/4.0/), which permits unrestricted use, distribution, and reproduction in any medium, provided the original work is properly cited.

### A narrative review of post-trauma resilience and optimism frameworks, and proposal of an integrated framework for musculoskeletal trauma

Wonjin Seo[a] and David Walton[b]

[a]Department of Health and Rehabilitation Sciences, Western University, London, Ontario, Canada; [b]School of Physical Therapy, Western University, London, Ontario, Canada

**Introduction/Aim**: Recovery after acute injury or trauma can be predicted through patient-reported measurement of pain severity, distress, catastrophizing, fear, and other negative cognitive constructs. These have led to accurate prognostic tools for identification of patients at greatest risk of chronic pain following acute trauma. While accurate, they are couched within traditional biomedical models of finding what is 'at fault' in a person and intervening to remove it. Our team have questioned this approach of measuring the 'terrible-ness' of such experiences, instead considering the potential for better outcomes when employing a strengths-based approach from a positive psychology lens.

**Methods**: We conducted a narrative review of existing literature about musculoskeletal (MSK) trauma, resilience and optimism. We identified three extant resilience models from major trauma (e.g., war, torture, rape) and considered how the elements of those could be integrated into acute post-MSK trauma and pain research.

**Results**: A new model of post-trauma resilience and optimism (PTRO) in MSK trauma has been proposed that considers three distinct trajectories of post-trauma recovery: exacerbation, maintenance, or improvement in pain severity, each informed by constructs of resilience and optimism. We comment on how the model could represent a new era of pain prognosis through strengths-based measurement and intervention strategies and reduce experiences of stigma in patients who are deemed high risk by traditional distress vulnerability models.

**Discussion/Conclusions**: We believe a new PTRO model holds relevance for post-MSK trauma and recovery prognosis and treatment.

**CONTACT** Wonjin Seo wseo@uwo.ca

© 2021 The Author(s). Published with license by Taylor & Francis Group, LLC.
This is an Open Access article distributed under the terms of the Creative Commons Attribution License (http://creativecommons.org/licenses/by/4.0/), which permits unrestricted use, distribution, and reproduction in any medium, provided the original work is properly cited.

### Preoperative Memory Specificity Predicts Pain Status One Month after Major Surgery

Anna Waisman[a], Valery Kleiman[b], Hance Clarke[c], P. Maxwell Slepian[d], and Joel Katz[d]

[a]Department of Psychology, York University, Toronto, Ontario, Canada; [b]Department of Anesthesia and Pain Management, Toronto General Hospital, and Altum Health, Neurology Specialty Clinic, Toronto Western Hospital, Toronto, ON, Canada; [c]Department of Anesthesia and Pain

Management, Toronto General Hospital, and Department of Anesthesiology and Pain Medicine, University of Toronto, Toronto, ON, Canada; [d]Department of Anesthesia and Pain Management, Toronto General Hospital, and Department of Psychology, York University, Toronto, ON, Canada

**Introduction**: Few studies have investigated autobiographical memory representation in individuals with chronic pain. Moreover, nothing is known about memory function and its relation to pain in individuals undergoing major surgery.

**Aim**: To examine the temporal relationship between autobiographical memory test (AMT) results in individuals scheduled for major surgery and pain status 1 month after surgery.

**Methods**: Patients were recruited as part of a larger study examining psychosocial predictors of chronic postsurgical pain (CPSP). After obtaining REB approval and consent, 97 adults (mean age=55.2±10.8 years; number of females=37) were administered the AMT before and 1 month after major surgery when they were asked to provide a specific personal memory in response to pain-related and positive cues. Memories were coded according to specificity. Pain status at the 1-month assessment was determined by patient self-report. Results show mean±sd.

**Results**: 3-way ANCOVA, using 1-month pain status, preoperative cue type, and memory category as factors, and preoperative pain status as the covariate, showed a significant 2-way 1-month pain status by preoperative memory category interaction (p=0.003). Simple effects showed that significantly fewer specific memories and significantly more general memories were generated at the preoperative assessment among patients who went on to report pain 1 month after surgery (specific: 1.59 ±1.27; general: 0.87±0.94) than those who did not report pain (specific: 2.22±1.43; general: 0.53±0.70)(both p=0.03).

**Discussion/Conclusions**: Individuals with an over-general retrieval style are more likely to experience pain after major surgery than those who recall specific memories. Autobiographical memory style might be a factor that initiates/maintains CPSP.

### ORCID

Joel Katz http://orcid.org/0000-0002-8686-447X

**CONTACT** Anna Waisman anna79@my.yorku.ca

© 2021 The Author(s). Published with license by Taylor & Francis Group, LLC.
This is an Open Access article distributed under the terms of the Creative Commons Attribution License (http://creativecommons.org/licenses/by/4.0/), which permits unrestricted use, distribution, and reproduction in any medium, provided the original work is properly cited.

### Presurgical Somatization Predicts Chronic Postsurgical Pain Intensity and Disability six Months after Cardiothoracic Surgery

Andrea Aternali[a], Maxwell Slepian[b], Hance Clarke[b], Karim Ladha[c], Rita Katznelson[b], Karen McRae[b], Ze'ev Seltzer[d], and Joel Katz[e]

[a]Department of Psychology, York University, Toronto, ON, Canada; [b]Department of Anesthesia and Pain Management, Toronto General Hospital, Toronto, ON, Canada; [c]Department of Anesthesia and Pain Management, St. Michael's Hospital, Toronto, ON, Canada; [d]Faculties of Medicine and Dentistry, University of Toronto Centre for the Study of Pain, Toronto, ON, Canada, and Central Institute of Mental Health, University of Heidelberg, Mannheim, Germany; [e]Department of Anesthesia and Pain Management, Toronto General Hospital, and Department of Psychology, York University, Toronto, ON, Canada

**Introduction/Aim**: Chronic postsurgical pain (CPSP) arising from cardiothoracic surgery affects millions of adults worldwide and is extremely difficult to treat. The current prospective, longitudinal study investigated presurgical somatization as a risk factor for CPSP after cardiothoracic surgery while controlling for several other potential psychosocial predictors of CPSP.

**Methods**: Participants included adults undergoing non-emergency cardiac or thoracic surgery (n = 546) who were followed over six months. Prior to surgery, participants responded to demographic, clinical, and psychological questionnaires. Six months after surgery, participants reported the intensity of their CPSP on a 0-10 numeric rating scale (NRS) and pain disability, measured by the Pain Disability Index (PDI). Multinomial logistic regression analyses were conducted to evaluate the degree to which presurgical factors, including somatization, measured by the Somatization subscale of the Symptoms Checklist-90-Revised (SCL-90-R), predicted pain outcomes (zero, mild, moderate, severe) six months after cardiothoracic surgery.

**Results**: Age, $\chi^2$ (3) = 7.90, $p < .05$, and the Somatization score $\chi^2$ (3) = 26.51, $p < .001$ predicted pain intensity (Nagelkerke $R^2$ = .26), whereas only the presurgical Somatization score, $\chi^2$ (3) = 19.43, $p < .001$, predicted pain disability (Nagelkerke $R^2$ = .28). Younger age was associated with greater odds of having mild pain versus zero pain. Likewise, the odds of having mild, moderate, and severe pain intensity or disability versus zero pain intensity or disability increased with the Somatization score.

**Discussion/Conclusions**: The present findings demonstrate that presurgical somatization leads to greater chronic pain and disability following cardiothoracic surgery.

**CONTACT** Andrea Aternali aternali@yorku.ca

© 2021 The Author(s). Published with license by Taylor & Francis Group, LLC.
This is an Open Access article distributed under the terms of the Creative Commons Attribution License (http://creativecommons.org/licenses/by/4.0/), which permits unrestricted use, distribution, and reproduction in any medium, provided the original work is properly cited.

### Age differences in the use of coping strategies in response to pain

Louise I. R. Castillo, Delaine A. Shackleton, and Thomas Hadjistavropoulos

Psychology, University of Regina, Regina, Saskatchewan, Canada

**Introduction/Aim**: The magnitude and duration of conditioned pain modulation (CPM) likely depends on the nature and intensity of the conditioning stimulus (CS). The aim of this study was to measure the effect of CS intensity on the duration of CPM, and to determine whether initial CPM magnitude correlates with CPM duration.

**Methods**: In this single-blind, non-randomized, repeated measures study, we assessed CPM hypoalgesia in 20 healthy participants following cold pressor tests (CPT) at 7°C and 12°C. The test stimulus, a 60 second heat stimulation, was administered before the CPT and immediately after, and again at 5-minute intervals until participants' pain scores returned to pre-CS levels.

**Results**: CPM lasted on average 32 minutes following the 7°C CPT and 20 minutes following the 12°C CPT ($p=.06$). For both conditions (7°C and 12°C CPT), there was a significant correlation between CPM duration and CPM magnitude at t=5 min post-CS (.65 ≤ all Spearman's $\rho$ ≤ .75; all p's <.002), but not between CPM duration and CPM magnitude at t=1 min post-CS (all p's >.50; all Spearman's $\rho$ <.15).

**Discussion/Conclusions**: The more intense CS did not induce a CPM of longer duration compared to the less intense CS, although there was a trend in that direction. Clinicians and researchers may be able to infer valuable information regarding their patients' or participants' CPM duration simply by measuring its magnitude 5 minutes post-CS.

**CONTACT** Louise Castillo castilll@uregina.ca

© 2021 The Author(s). Published with license by Taylor & Francis Group, LLC.
This is an Open Access article distributed under the terms of the Creative Commons Attribution License (http://creativecommons.org/licenses/by/4.0/), which permits unrestricted use, distribution, and reproduction in any medium, provided the original work is properly cited.

### Effect of Conditioning Stimulus Intensity on the Duration of Conditioned Pain Modulation (CPM)

Alexia Coulombe-Leveque, Yannick Tousignant-Laflamme, Serge Marchand, and Guillaume Léonard

Faculty of Medicine and Health Sciences, Université de Sherbrooke, Sherbrooke, QC, Canada

**Introduction/Aim**: The magnitude and duration of conditioned pain modulation (CPM) likely depends on the nature and intensity of the conditioning stimulus (CS). The aim of this study was to measure the effect of CS intensity on the duration of CPM, and to determine whether initial CPM magnitude correlates with CPM duration.

**Methods**: In this single-blind, non-randomized, repeated measures study, we assessed CPM hypoalgesia in 20 healthy participants following cold pressor tests (CPT) at 7°C and 12°C. The test stimulus, a 60 second heat stimulation, was administered before the CPT and immediately after, and again at 5-minute intervals until participants' pain scores returned to pre-CS levels.

**Results**: CPM lasted on average 32 minutes following the 7°C CPT and 20 minutes following the 12°C CPT ($p=.06$). For both conditions (7°C and 12°C CPT), there was a significant correlation between CPM duration and CPM magnitude at t=5 min post-CS (.65 ≤ all Spearman's $\rho$ ≤ .75; all p's <.002), but not between CPM duration and CPM magnitude at t=1 min post-CS (all p's >.50; all Spearman's $\rho$ <.15).

**Discussion/Conclusions**: The more intense CS did not induce a CPM of longer duration compared to the less intense CS, although there was a trend in that direction. Clinicians and researchers may be able to infer valuable information regarding their patients' or participants' CPM duration simply by measuring its magnitude 5 minutes post-CS.

### ORCID

Alexia Coulombe-Leveque http://orcid.org/0000-0001-6835-0646
Yannick Tousignant-Laflamme http://orcid.org/0000-0002-1133-8707
Serge Marchand http://orcid.org/0000-0002-4234-5977
Guillaume Léonard http://orcid.org/0000-0002-3625-3072

**CONTACT** Alexia Coulombe-Leveque Alexia.Coulombe-Leveque@USherbrooke.ca

© 2021 The Author(s). Published with license by Taylor & Francis Group, LLC.
This is an Open Access article distributed under the terms of the Creative Commons Attribution License (http://creativecommons.org/licenses/by/4.0/), which permits unrestricted use, distribution, and reproduction in any medium, provided the original work is properly cited.

### A neuronal mechanism of spinal hyperexcitability is sexually dimorphic in rodent and human models of pathological pain

Annemarie Dedek[a,b], Jian Xu[c], Louis-Étienne Lorenzo[d], Antoine G. Godin[d,e], Chaya M. Kandegedara[a,b], Geneviève Glavina[d], Jeffrey A. Landrigan[a], Chris Dedek[a], Paul J. Lombroso[c], Yves De Koninck[d,e,f], Eve C. Tsai[g,h,i], and Michael E. Hildebrand[a,b]

[a]Neuroscience, Carleton University, Ottawa, Ontario, Canada; [b]Ottawa Hospital Research Institute, Ottawa, Ontario, Canada; [c]Child Study Center, Yale University School of Medicine, New Haven, Connecticut, United States; [d]CERVO Brain Research Centre, Quebec Mental Health Institute, Quebec, QC, Canada; [e]Department of Psychiatry and Neuroscience, Université Laval, Quebec, QC, Canada; [f]Graduate Program in Neurobiology, Université Laval, Quebec, QC, Canada; [g]Ottawa Hospital Research Institute, Neuroscience Program, Ottawa, Ontario, Canada; [h]Brain and Mind Research Institute, University of Ottawa, Ottawa, Ontario, Canada; [i]Department of Surgery, Division of Neurosurgery, The Ottawa Hospital, Ottawa, Ontario, Canada

**Introduction/Aim**: The neurobiological underpinnings of sexually dimorphic pain signalling remain unclear in rodents and are virtually unexplored in human preclinical models. Within the nociceptive system, the superficial dorsal horn (SDH) is a critical site of pain modulation. We have recently characterized a pathological pain pathway in male rat and human SDH neurons where brain-derived neurotrophic factor (BDNF) mediates a loss of inhibition and a subsequent potentiation of GluN2B-containing N-methyl D-aspartate receptors (NMDARs) (Dedek, Xu *et al.*, Brain, 2019). Here, we investigate whether molecular mechanisms of spinal hyperexcitability are conserved between sexes in both rodents and humans.

**Methods**: We studied spinal mechanisms of pain processing using the *ex vivo* BDNF pain pathology model in rodent and human spinal cord and the *in vivo* Freund's adjuvant (CFA) rodent inflammatory pain model. We paired patch-clamp electrophysiological recordings of synaptic NMDAR responses with *ex vivo* pharmacology and biochemical approaches as well as behavioural testing.

**Results**: In female rats, NMDAR responses at lamina I SDH synapses are not potentiated by spinal cord *ex vivo* treatment with BDNF nor in the *in vivo* CFA inflammatory pain model. Parallel biochemical evidence shows no differences in female rodent (CFA and *ex vivo* BDNF) as well as human (*ex vivo* BDNF) models of pathological pain when compared to non-pain tissue. Electrophysiology and biochemical investigations in ovariectomized rats suggest hormonal mediation of this sex-difference.

**Discussion/Conclusions**: We conclude that neuronal mechanisms of SDH hyperexcitability are sexually dimorphic in rats and humans. This sex-difference in underpinning neurobiological mechanisms of chronic pain has profound implications for the development of novel pain therapeutics.

**CONTACT** Annemarie Dedek annemariededek@cmail.carleton.ca

© 2021 The Author(s). Published with license by Taylor & Francis Group, LLC.
This is an Open Access article distributed under the terms of the Creative Commons Attribution License (http://creativecommons.org/licenses/by/4.0/), which permits unrestricted use, distribution, and reproduction in any medium, provided the original work is properly cited.

### Peak alpha frequency and power within the dynamic pain connectome show sex differences and abnormalities in neuropathic pain.

Camille Fauchon[a], Junseok A. Kim[a], Lee B. Kisler[a], Rima El-Sayed[b], Natalie Osborne[b], Anton Rogachov[b], Joshua C. Cheng[b], Kasey S. Hemington[b], Rachael L. Bosma[b], Benjamin T. Dunkley[c], Jiwon Oh[d], Anuj Bhatia[e], Robert D. Inman[f], and Karen Deborah Davis[g]

[a]Krembil Brain Institute, University Health Network, Toronto, Canada; [b]Krembil Brain Institute, University Health Network, Institute of Medical Science, University of Toronto, Toronto, Canada; [c]Neurosciences & Mental Health Program, Diagnostic Imaging, The Hospital for Sick Children Research Institute, Department of Medical Imaging, University of Toronto, Toronto, ON, Canada; [d]Division of Neurology, Department of Medicine, St. Michael's Hospital, Toronto, ON, Canada; [e]Department of Anesthesia and Faculty of Medicine, University of Toronto, Department of Anesthesia and Pain Medicine, Toronto Western Hospital, Toronto, ON, Canada; [f]Institute of Medical Science and Department of Medicine, University of Toronto; Arthritis Institute, Krembil Research Institute, University Health Network, Toronto, ON, Canada; [g]Krembil Brain Institute, University Health Network, Institute of Medical Science, and Dept. Surgery, University of Toronto, Toronto, Canada

**Introduction/Aim**: We previously identified sex differences in brain network organization in healthy individuals and those with chronic pain. We also identified alpha band slowing and power changes in the dynamic pain connectome (DPC) in chronic pain. Here we tested the hypothesis that slower and higher power peak alpha frequency (PAF) is a general feature across neuropathic pain (NP) conditions but show sex differences.

**Methods**: Fifty participants (25 males, 25 females) with NP (multiple sclerosis, ankylosing spondylitis, carpel tunnel syndrome, other neuropathic etiologies) and 50 sex-matched healthy controls (HC) underwent resting state magnetoencephalography. Regional spectral power analyses (4-60Hz) determined the peak alpha (8-13Hz) frequency and power in the ascending nociceptive pathway (ANP: S1, S2, posterior insula), default mode (DMN: PCC, precuneus, mPFC) and salience networks (SN: anterior insula, TPJ, MCC), and the subgenual anterior cingulate cortex (sgACC).

**Results**: Across pain conditions, NP was associated with increased PAF power in the ANP, SN and DMN as compared to HCs, but specific abnormalities depended on sex. Sex differences in the NP group included females exhibiting lower PAF power in the sgACC, and faster PAF in the ANP and SN. Within-sex analyses found that females had NP-related increased PAF power in the ANP, SN, and DMN, whereas males had NP-related increased PAF power in the ANP and slower PAF in the DMN and ANP.

**Discussion/Conclusions**: These data highlight NP and sex-specific abnormalities in the power and speed of alpha oscillations across the DPC that could underlie aberrant neuronal communication in nociceptive sensing and modulation.

### ORCID

Karen Deborah Davis http://orcid.org/0000-0003-1879-0090

**CONTACT** Annemarie Dedek annemariededek@cmail.carleton.ca

© 2021 The Author(s). Published with license by Taylor & Francis Group, LLC.
This is an Open Access article distributed under the terms of the Creative Commons Attribution License (http://creativecommons.org/licenses/by/4.0/), which permits unrestricted use, distribution, and reproduction in any medium, provided the original work is properly cited.

### Multidimensional domains of pain in advanced liver disease: A scoping review

Franklin F. F. Gorospe IV[a], Laura Istanboulian[b], Martine Puts[c], David K. H. Wong[d], Elizabeth Lee[d], and Craig Dale[e]

[a]University of Toronto, Lawrence S. Bloomberg Faculty of Nursing | Perioperative Services, Toronto General Hospital – University Health Network, Toronto, Ontario, Canada; [b]University of Toronto, Lawrence S. Bloomberg Faculty of Nursing | Acute Inpatient Respiratory Unit, Michael Garron Hospital, Toronto, Ontario, Canada; [c]University of Toronto, Lawrence S. Bloomberg Faculty of Nursing, Toronto, Ontario, Canada; [d]Hepatology Division, Toronto General Hospital – University Health Network, Toronto, Ontario, Canada; [e]University of Toronto, Lawrence S. Bloomberg Faculty of Nursing | Department of Critical Care, Sunnybrook Health Sciences Centre, Toronto, Ontario, Canada

**Introduction/Aim**: Pain is a significant problem for adults with advanced liver disease. Unrelieved pain can reduce the quality of living and dying among this growing population. There is a lack of research exploring the multidimensional domains of pain. The aim of this scoping review is to map the domains of pain using a multidimensional model.

**Methods**: Using Arksey and O'Malley's scoping review framework, a rigorous and systematic search of the literature between 1990-2019 in AMED, CINAHL, Embase, Medline, and gray sources were conducted. Selection and extraction of data were performed by two independent reviewers.

**Results**: Of 10,037 studies, 43 met inclusion criteria. Mapping results indicate physical domain was identified in 81%, psychological domain in 65%, and sociocultural in 5% of the studies. There was scant (9%) use of multi-dimensional pain appraisal tools. Minimal pain characteristics were reported; pain classification (9%), location (9%), and intensity (16%). Few (9%) studies focused on pain as a primary outcome, instead, pain was reported as a secondary outcome within disease burden and part of another health-related quality of life measure. Most commonly reported recommendations are frequent and detailed assessments (14%), multimodal pain management strategies (14%), and use of pharmacological agents to treat physical pain (14%).

**Discussion/Conclusions**: There is a lack of research focused on pain as a multidimensional construct. Little research focused on pain as a primary outcome for patients with liver disease. Furthermore, there is scant qualitative and mixed methods research investigating the patient's experience of pain. Future research should explore multi-dimensional approaches to pain appraisal and management.

### ORCID

Franklin F. F. Gorospe IV http://orcid.org/0000-0002-3520-3196
Craig Dale http://orcid.org/0000-0002-5042-0332

**CONTACT** Franklin Gorospe franklin.gorospe@mail.utoronto.ca

© 2021 The Author(s). Published with license by Taylor & Francis Group, LLC.
This is an Open Access article distributed under the terms of the Creative Commons Attribution License (http://creativecommons.org/licenses/by/4.0/), which permits unrestricted use, distribution, and reproduction in any medium, provided the original work is properly cited.

### Temporal summation and conditioned pain modulation are affected by sex and stimulation type

Monica Sean[a], Matthieu Vincenot[b], Alexia Coulombe-Lévêque[a], Marylie Martel[a], Louis Gendron[b], Serge Marchand[c], and Guillaume Léonard[d]

[a]Université de Sherbrooke, Faculty of Medicine and Health Sciences, Sherbrooke, Québec, Canada; [b]Université de Sherbrooke, Pharmacology-Physiology, Sherbrooke, Québec, Canada; [c]Université de Sherbrooke, Surgery, Sherbrooke, Québec, Canada; [d]Université de Sherbrooke, Rehabilitation, Sherbrooke, Québec, Canada

**Introduction/Aim**: The influence of biological sex on excitatory and inhibitory mechanisms of pain modulation remains debated. These conflicting results could be explained by the type of paradigm used to test these excitatory and inhibitory pain responses. The aim of this study was to shed light on these inconsistencies by comparing the temporal summation of pain (TSP; excitatory mechanism) and conditioned pain modulation (CPM; inhibitory mechanism) between men and women using both thermal and electrical paradigms.

**Methods**: 50 healthy participants (25 men) attended 2 experimental sessions. TSP and CPM were measured using two different paradigms, in which a test stimulus (TS) was applied before and after a conditioning stimulus (CS). In one paradigm (thermal stimulations), the TS consisted of a constant noxious heat stimulus generated by the thermode, and the CS consisted of a cold pressor test (CPT). In the other paradigm (electrical stimulations), the TS and CS consisted of electrical impulses applied transcutaneously.

**Results**: Independent t-tests comparing women and men revealed group differences for the electrical stimulation paradigm ($p=0.04$); a sex effect was also observed for the thermal stimulation paradigm, although the difference failed to reach statistical significance ($p=0.12$). CPM responses were similar across men and women both for the thermal ($p=0.98$) and electrical ($p=0.65$) stimulations.

**Discussion/Conclusions**: Similar conclusions are obtained regarding sex differences for TSP and CPM responses, independently of the stimulation paradigm used. Electrical stimulations can potentially highlight more easily sex differences in TSP responses.

**CONTACT** Monica Sean monica.sean@usherbrooke.ca

© 2021 The Author(s). Published with license by Taylor & Francis Group, LLC.
This is an Open Access article distributed under the terms of the Creative Commons Attribution License (http://creativecommons.org/licenses/by/4.0/), which permits unrestricted use, distribution, and reproduction in any medium, provided the original work is properly cited.

### Reductions in negative affect and sleep problems as determinants of clinical responses to medical cannabis among patients with chronic pain

Romina Sotoodeh[a], Antonio Vigano[b], Michelle Canac-Marquis[c], Rihab Gamaoun[d], Maja Kalaba[e], Lilach Eyal Waldman[f], Jordi Perez[g], Yoram Shir[h], Andree Neron[i], Pierre Beaulieu[j], Mark A. Ware[k], and Marc O Martel[l]

[a]McGill University, Faculty of Dentistry, Montreal, Quebec, Canada; [b]McGill University, Departments of Medicine and Oncology, Montreal, Quebec, Canada; [c]Research Institute of the McGill University Health Centre, Montreal, Quebec, Canada; [d]Canopy Growth Corporation, Real World Evidence, Montreal, Quebec, Canada; [e]Montreal General Hospital, Alan Edwards Pain Management Unit, Montreal, Quebec, Canada; [f]Montreal General Hospital, Alan Edwards Pain Management Unit, McGill University, Department of Anesthesia, Montreal, Quebec, Canada; [g]Centre Universitaire de L'Université de Montréal, Pain Center, Montreal, Quebec, Canada; [h]Université de Montréal, Departments of Pharmacology, and Physiology, and Anesthesiology and Pain Medicine, Montreal, Quebec, Canada; [i]Canopy Growth Corporation, Global Medical Divison, Montreal, Quebec, Canada; [j]McGill University, Faculty of Dentistry and Department of Anesthesia, Montreal, Quebec, Canada; [k]xxx; [l]xxx

**Introduction/Aim**: Evidence is accumulating supporting the analgesic benefits of medical cannabis for patients with chronic pain, but little is known on the factors that contribute to these analgesic benefits. Given that symptoms of depression and anxiety (i.e., negative affect) and sleep problems are linked to heightened clinical pain intensity, the main objective of this study was to examine whether reductions in negative affect (NA) and sleep problems contributed to reductions in pain intensity following medical cannabis initiation. We first examined whether reductions in NA and sleep problems contributed to reductions in "average" pain intensity. The contribution of these variables to clinically important reductions (i.e. 20-30%) in pain intensity was then examined.

**Methods**: In this longitudinal study, chronic non-cancer pain patients (n = 2037) using medical cannabis completed measures assessing sociodemographic, lifestyle, medical, and psychological variables. Measures assessing clinical pain intensity, NA, and sleep interference due to pain were completed at baseline and every 3 months following medical cannabis initiation.

**Results**: Multiple regression analyses indicated that reductions in NA and sleep interference were associated with reductions in "average" pain intensity across all follow-up time points (all ps $< 0.05$). Reductions in NA and sleep interference were also associated with clinically important reductions in pain intensity (all ps $< 0.05$). These findings remained significant after controlling for sociodemographic, lifestyle, and medical variables (all ps $< 0.05$).

**Discussion/Conclusions**: Our preliminary findings suggest that reductions in NA and sleep problems contribute to the analgesic benefits of medical cannabis in chronic pain patients.

**CONTACT** Romina Sotoodeh Romina.Sotoodeh@mail.mcgill.ca

© 2021 The Author(s). Published with license by Taylor & Francis Group, LLC.
This is an Open Access article distributed under the terms of the Creative Commons Attribution License (http://creativecommons.org/licenses/by/4.0/), which permits unrestricted use, distribution, and reproduction in any medium, provided the original work is properly cited.

### The Development of Image-Based Biomarkers using 4DCT scans to Differentiate between Healthy and Previously Fractured Wrists.

Lauren Straatman[a], Nik Knowles[b], David Walton[c], and Emily Lalone[d]

[a]Western University, Health and Rehabilitation Science, London, ON, Canada; [b]University of Calgary, Cumming School of Medicine, Calgary, AB, Canada; [c]Western University, Health and Rehabilitation Science & School of Physical Therapy, London, ON, Canada; [d]Western University, Mechanical and Materials Engineering, London, ON, Canada

**Introduction/Aim**: Four-dimensional computed tomography (4DCT) is used to acquire kinematic joint contact and depth-specific bone mineral density (BMD) metrics. The goal of this study is to highlight the value of 4DCT in differentiating between healthy people and people experiencing pain post-fracture through the demonstration of key differences that can be observed through BMD and joint contact area (JCa).

**Methods**: To demonstrate the utility of this new imaging technique, 4 participants (2 without wrist pain and 2 with persistent post-trauma wrist pain) underwent 4DCT scans of their dominant wrist. The images obtained were used to create 3D bone models and joint contact maps wherein average BMD and JCa data was collected from the radiolunate (RL) and radioscaphoid (RS) fossae. Regional analyses were performed across 3 normalized layers in relation to depth from the subchondral surface.

**Results**: Overall, our healthy participants demonstrated higher BMD and a larger JCa in the RL and RS for all regional analyses and all ranges of motion. BMD in the healthy group was 88.36, 208.16 and 319.63 mg/cm$^3$ $K_2HPO_4$ and 115.92, 210.86 and 260.17 mg/cm$^3$ $K_2HPO_4$ higher in the RL and RS fossae, respectively. JCa was 36.72 and 51.30 mm$^2$ larger in the healthy population on the RL and RS fossae, respectively.

**Discussion/Conclusions**: Preliminary results demonstrate the value of 4DCT in characterizing large quantitative differences in BMD and JCa in our study population. Additional participants will enable us to make more definitive conclusions regarding the potential of 4DCT in differentiating between healthy wrists and those experiencing pain following wrist trauma.

© 2021 The Author(s). Published with license by Taylor & Francis Group, LLC.
This is an Open Access article distributed under the terms of the Creative Commons Attribution License (http://creativecommons.org/licenses/by/4.0/), which permits unrestricted use, distribution, and reproduction in any medium, provided the original work is properly cited.

### Social support: A potential protective factor for opioid use problems in patients with chronic pain

Maria Verner[a], Amanda Sirois[b], Katherine Scott[c], Alice Bruneau[d], Leah Frimerman[d], Catherine Fournier[a], Jordi Perez[e], Yoram Shir[e], and Marc O Martel[a]

[a]McGill University, Faculty of Dentistry, Montreal, Quebec, Canada; [b]McGill University, School of Human Nutrition, Montreal, Quebec, Canada; [c]McGill University, Department of Physiology, Montreal, Quebec, Canada; [d]McGill University, Division of Experimental Medicine, Montreal, Quebec, Canada; [e]McGill University, Department of Anesthesia, Montreal, Quebec, Canada

**Introduction/Aim**: Despite the potential benefits of opioids, the use of opioids may be accompanied by opioid use problems such as prescription opioid misuse (POM) and addiction. Some protective factors may prevent opioid use problems, but these factors have yet to be fully elucidated. One such potential protective factor is social support. The first objective of this study was to examine whether living status (i.e., living with a partner versus alone) is associated with POM. The second objective was to examine the association between perceived social support and POM.

**Methods**: In this longitudinal study, patients (n = 103) prescribed opioids had their level of social support and living status assessed at baseline. Patients then completed daily diary measures of opioid craving for 14 consecutive days and a questionnaire assessing opioid misuse behaviours.

**Results**: Analyses showed that POM behaviours did not differ significantly as a function of patients' living status ($t$ = 1.87, p = .06). However, analyses indicated that perceived support among those living with a partner was associated with decreased opioid misuse (r = -.315, p = .033). Furthermore, patients' perceptions of punishment responses from significant others were associated with heightened reports of opioid craving (r = .339, p = .021).

**Discussion/Conclusions**: Our findings suggest that living with a partner could potentially be a protective factor against opioid misuse. Furthermore, our findings suggest that partners' solicitousness might contribute to reducing patients' opioid misuse behaviours. Taken together, this set of findings provide new insights into the social determinants of opioid misuse.

### ORCID

Maria Verner http://orcid.org/0000-0002-3498-0751

CONTACT Maria Verner maria.verner@mail.mcgill.ca

© 2021 The Author(s). Published with license by Taylor & Francis Group, LLC.
This is an Open Access article distributed under the terms of the Creative Commons Attribution License (http://creativecommons.org/licenses/by/4.0/), which permits unrestricted use, distribution, and reproduction in any medium, provided the original work is properly cited.

### Parent Perceived Benefits of a Cognitive-Behavioural Group Intervention for Parents of Youth with Chronic Pain: A Thematic Analysis

Nicole Walker, Soeun Lee, and C. Meghan McMurtry

University of Guelph, Department of Psychology, Guelph, Ontario, Canada

**Introduction/Aim**: Parents of youth with chronic pain experience emotional distress, increased financial hardship, and disruptions to social and family functioning. Moreover, parental suffering adversely impacts a youth's ability to cope with chronic pain. Parent-targeted interventions are less studied in the literature, though preliminary evidence suggests that group interventions can have positive impacts on parents' self-reported responses to pain and psychological flexibility. However, what is less clear are parents' perceptions of the benefits of a group intervention. This study aims to describe qualitative reflections from parents regarding their perceived benefits of a cognitive-behavioural group intervention.

**Methods**: Parents of youth aged 8-17 with chronic pain were recruited from an outpatient pediatric pain clinic and participated in a five-week cognitive behavioural-based group intervention. Parents completed a 45-60 min semi-structured focus group or individual interview to gather feedback regarding the benefits of the parent group. Data analysis will follow Braun and Clarke's six-step reflexive thematic analysis procedure.

**Results**: Data collection is ongoing; ten focus groups/ interviews including 17 parents have been completed to date. All audio data will be transcribed by December 2020, with coding and data analysis complete by February 2021. It is hypothesized that parents will report perceived benefits including: feeling emotionally supported, indirect benefits for their youth, and coherence among parents.

**Discussion/Conclusions**: The results of this study will inform parent interventions within pediatric chronic pain health care. Understanding the perceived benefits of a parent intervention may help increase the level of parental involvement and satisfaction with existing treatment approaches.

### ORCID

C. Meghan McMurtry http://orcid.org/0000-0002-3278-1169

© 2021 The Author(s). Published with license by Taylor & Francis Group, LLC.
This is an Open Access article distributed under the terms of the Creative Commons Attribution License (http://creativecommons.org/licenses/by/4.0/), which permits unrestricted use, distribution, and reproduction in any medium, provided the original work is properly cited.

### The relationship between pain-related threat and motor behavior in non-specific low back pain. A systematic review and meta-analysis

Patrick Ippersiel, Anthony Teoli, Timothy Wideman, Richard Preuss, and Shawn Robbins

McGill University, School of Physical and Occupational Therapy, Montreal, Quebec, Canada

**Introduction/Aim**: While pain-related fear and catastrophizing are predictors of disability in low back pain (LBP), their relationship with guarded motor behavior is unclear. The aim of this meta-analysis was to determine the relationship between pain-related threat (via pain-related fear and catastrophizing) and motor behavior in adults with LBP.

**Methods**: MEDLINE, Embase, PsychINFO and CINAHL databases were searched to October 2019. Included studies measured the association between pain-related fear or pain catastrophizing and motor behavior (spinal range of motion, trunk coordination and variability, muscle activity) during movement, in adults with LBP. The Newcastle-Ottawa Scale assessed for risk of bias. Correlation coefficients were pooled using the random-effects model.

**Results**: Reduced spinal range of motion during flexion-tasks was weakly related to pain-related fear (14 studies, r=-0.20, 95% confidence interval=-0.30 to -0.098, P<0.001) and pain catastrophizing (6 studies, r=-0.25, 95% confidence interval=-0.40 to -0.085, P<0.001). Greater trunk extensor muscle activity during bending was moderately related to pain-related fear (2 studies, r=-0.40, 95% confidence interval=-0.55 to -0.23, P<0.001). Pain catastrophizing, but not fear, was related to higher trunk activity during gait (2 studies, r=0.25, 95% confidence interval=0.063 to 0.42, P<0.013). Methodological differences and missing data limited robust syntheses of studies examining muscle activity.

**Discussion/Conclusions**: We found a weak to moderate relationship between pain-related threat and guarded motor behavior during flexion-based tasks, but not consistently during other movements. Future clinical research should build on these findings by exploring the advantages of integrated treatment strategies that target both psychological and motor behavior processes, compared to traditional approaches.

### ORCID

Shawn Robbins http://orcid.org/0000-0002-8108-0561

**CONTACT** Patrick Ippersiel Patrick.ippersiel@mail.mcgill.ca

© 2021 The Author(s). Published with license by Taylor & Francis Group, LLC.
This is an Open Access article distributed under the terms of the Creative Commons Attribution License (http://creativecommons.org/licenses/by/4.0/), which permits unrestricted use, distribution, and reproduction in any medium, provided the original work is properly cited.

### Chronic pain needs assessment in Saskatchewan: hearing experiences of people with lived experience, healthcare providers, and decision-makers in three communities

Jessica Jack[a], Cassie Jones[b], Megan Hewson[c], Sharon Okeeweehow[d], Erin Beckwell[d], Ross McCreery[e], Krista Baerg[f], Karen Juckes[g], Jeannie Coe[h], Colleen Dell[i], Pam Downe[a], Karen Lawson[j], Cristina Ugolini[h], and Susan M Tupper[h]

[a]University of Saskatchewan, Archaeology and Anthropology, Saskatoon, Saskatchewan, Canada; [b]University of Saskatchewan, College of Nursing, Yorkton, Saskatchewan, Canada; [c]Saskatchewan Health Authority, Regina, Saskatchewan, Canada; [d]Patient Partner, Saskatoon, Saskatchewan, Canada; [e]Patient Partner, Regina, Saskatchewan, Canada; [f]University of Saskatchewan, Pediatrics, College of Medicine, Saskatoon, Saskatchewan, Canada; [g]University of Saskatchewan, College of Nursing, Regina, Saskatchewan, Canada; [h]Saskatchewan Health Authority, Saskatoon, Saskatchewan, Canada; [i]University of Saskatchewan, Sociology, Saskatoon, Saskatchewan, Canada; [j]University of Saskatchewan, Psychology, Saskatoon, Saskatchewan, Canada

**Introduction/Aim**: Design and implementation of new health services must consider community-level context. To date, little work has been done to identify contextual factors influencing pain service design and create a scalable process for ongoing development and quality improvement. By bringing together people with lived experience (PwLE), healthcare providers (HCPs), community- based organizations (CBOs), and Saskatchewan Health Authority (SHA) Health Network decision-makers (HSDMs), this research program will support the co-design of sustainable services for PwLE in Saskatchewan.

**Methods**: Findings from phase 1 of a 4-phase project are reported. Interviews, following a semi-structured guide developed in collaboration with patient partners, were conducted with participants in Saskatoon's downtown core neighborhoods, Regina's pediatric services, and the Yorkton community. Qualitative analysis was used to identify context considerations, gaps, and service development priorities.

**Results**: Interview findings with 23 PwLE, 20 HCPs, 3 CBO representatives, and 3 HSDMs revealed struggles to navigate the health system, access and afford non-drug care, and overcome discrimination due to misconceptions about pain. Participants identified needs for population specific education for HCPs, increased awareness of available resources for pain care, support for service navigation, social supports, and reduction of healthcare siloes through improved communication and collaboration.

**Discussion/Conclusions**: Although the 3 communities identified similar improvement needs, development of services must address contexts of low-resource living conditions (e.g. safety, racial-discrimination), younger age (e.g. dismissal of pain concerns), and rural settings (e.g. lack of HCPs with pain expertise). In subsequent project phases, mixed working groups will design, implement, and evaluate a priority strategy to improve pain care in each community.

### ORCID

Susan M Tupper http://orcid.org/0000-0003-3736-357X

**CONTACT** Jessica Jack jessica.jack@usask.ca

© 2021 The Author(s). Published with license by Taylor & Francis Group, LLC.
This is an Open Access article distributed under the terms of the Creative Commons Attribution License (http://creativecommons.org/licenses/by/4.0/), which permits unrestricted use, distribution, and reproduction in any medium, provided the original work is properly cited.

### The Effects of Anxiety Sensitivity and Pain Anxiety on Sexual Functioning and Sexual Pain

Julia Keilty, Janine Olthuis, Lucia O'Sullivan, and Sandra Byers

University of New Brunswick, Psychology, Fredericton, New Brunswick, Canada

**Introduction/Aim**: Anxiety sensitivity (AS) refers to the fear of experiencing normal physiological arousal sensations (e.g., racing heart, rapid breathing) that evolves from the belief that these symptoms may have a catastrophic consequence. The limited research available suggests that high AS tends to be associated with greater sexual distress, lower sexual quality of life, and greater sexual pain. The current study aimed to evaluate further the association between AS and sexual functioning/pain in adult men and women by considering the role of pain anxiety in this association.

**Methods**: Participants were 357 adults aged 19-60 (*M* age = 29 years, 67% female) who completed an online self-report survey assessing psychological distress (e.g., pain anxiety and AS) and sexual functioning.

**Results**: Two mediation models guided data analysis: the first considered whether the relationship between AS and sexual functioning operates via pain anxiety, whereas the second mediation model considered whether the relationship between AS and sexual pain operates via pain anxiety. The results indicated that elevated levels of AS were associated with greater sexual dysfunction and greater sexual pain, which is congruent with other research in this area. However, pain anxiety did not appear to mediate these relationships, suggesting that the effect of AS on sexual pain and sexual functioning is not a result of its impact on pain anxiety.

**Discussion/Conclusions**: Overall, this study provides a better understanding of the mechanisms that may influence the associations between AS and sexual functioning/pain. Findings are discussed in terms of implications for counseling, education, and research.

### ORCID

Lucia O'Sullivan http://orcid.org/0000-0003-0124-6267

**CONTACT** Julia Keilty jkeilty@unb.ca

© 2021 The Author(s). Published with license by Taylor & Francis Group, LLC.
This is an Open Access article distributed under the terms of the Creative Commons Attribution License (http://creativecommons.org/licenses/by/4.0/), which permits unrestricted use, distribution, and reproduction in any medium, provided the original work is properly cited.

### A Cross-Sectional Survey and Quality Assessment of Cannabis Consumer Health Information for Pain Online

Jeremy Y Ng, Darragh A Dzisiak, and Jessica B Saini

Department of Health Research Methods, Evidence, and Impact, Faculty of Health Sciences, McMaster University, Hamilton, Ontario, Canada

**Introduction/Aim**: Cannabis has been proposed as a potential therapy for patients experiencing pain, despite evidence of concomitant negative health effects associated with long-term use. Those considering cannabis for pain relief often seek information online, but may lack the knowledge or ability to identify credible and accurate resources. The present study assesses the quality of web-based consumer health information (CHI) available at the intersection of cannabis and pain.

**Methods**: We conducted a cross-sectional quality assessment of website CHI. Three countries that have either partially legalized or decriminalized cannabis were searched using Google: Canada, the Netherlands and the United States. For each of the 9 searches, the first 3 pages of generated websites were screened for eligibility. Included websites contained CHI on cannabis for pain and were quality assessed in duplicate using the DISCERN instrument.

**Results**: Of the 270 webpages identified, 64 were unique, and 36 websites were deemed eligible for inclusion. The average summed DISCERN score was 48.85 out of 75 (SD=8.13), and the average overall score was 3.10 out of 5 (SD=0.62). Commercial websites (selling cannabis products/services) scored most poorly, while health portals scored most highly.

**Discussion/Conclusions**: The present study's findings suggest that the CHI present online at the intersection of cannabis and pain is of varying quality, which may pose a risk of potential harm to patient readers. For example, commercial websites typically only highlighted the advantages of cannabis products without discussing the risks. Healthcare providers should take an active role in guiding patients in identifying high-quality CHI resources.

### ORCID

Jeremy Y Ng http://orcid.org/0000-0003-0031-5873
**CONTACT** Jeremy Y. Ng ngjy2@mcmaster.ca

© 2021 The Author(s). Published with license by Taylor & Francis Group, LLC.
This is an Open Access article distributed under the terms of the Creative Commons Attribution License (http://creativecommons.org/licenses/by/4.0/), which permits unrestricted use, distribution, and reproduction in any medium, provided the original work is properly cited.

### Do adverse effects experienced by people living with chronic pain have a sex and gender?

Hermine Lore Nguena Nguefack[a], M. Gabrielle Pagé[b], Line Guénette[c], Lucie Blais[d], and Anaïs Lacasse[a]

[a]Département des sciences de la santé, Université du Québec en Abitibi-Témiscamingue (UQAT), Rouyn-Noranda, Québec, Canada; [b]Centre de recherche du Centre hospitalier de l'Université de Montréal (CRCHUM); Département d'anesthésiologie et de médecine de la douleur, Faculté de médecine, Université de Montréal, Montréal, Québec, Canada; [c]Faculté de pharmacie, Université Laval, Québec, Québec, Canada; [d]Faculté de pharmacie, Université de Montréal, Montréal, Québec, Canada

**Introduction/Aim**: The understanding of sex and gender differences in chronic pain (CP) treatment outcomes is most useful but understudied. This study aimed to explore the associations between sex, gender roles, and the number of severe adverse effects (SAE) reported by people living with CP.

**Methods**: The analysis was conducted using the COPE Cohort, a dataset arising from a province-wide recruitment of 1935 adults with CP who completed a web-based questionnaire between June and October 2019 (Quebec, Canada). Gender roles were measured using the Bem Sex-Role Inventory and four subgroups were formed applying the median split method. Nineteen potential pain medications adverse effects were assessed using a standardized checklist (none/mild/moderate/severe). A zero-inflated Poisson multivariable model allowed the assessment of sex and gender roles as potential predictors of the number of SAE.

**Results**: Among the 1343 participants reporting using pain medications, the number of SAE varied across sex (females: 1.57±2.12, males: 1.42±1.96, p=0.5849) and gender roles subgroups (feminine: 2.07±2.42, masculine: 1.09±1.73, undifferentiated: 1.45±2.05, androgynous: 1.46±2.04; p<.0001). Adjusting for clinical/treatment/sociodemographic factors, sex (females vs. males: ß=0.37, p=0.0005) and gender roles (feminine vs. androgynous: ß=-0.23, p=0.0059; masculine vs. androgynous: ß=-0.22, p=0.0351; undifferentiated vs. androgynous: ß=-0.26, p=0.0033) were associated with the number of SAE reported.

**Discussion/Conclusions**: Even if we cannot confirm if the associations found in this study are explained by differences in the experience or in the reporting of SAE, sex and gender roles appear to be independent social determinants that are relevant to explore when studying or trying to prevent pain medication SAE.

### ORCID

Line Guénette http://orcid.org/0000-0001-9769-7550
Anaïs Lacasse http://orcid.org/0000-0002-3992-5145

**CONTACT** Hermine Lore Nguena Nguefack HermineLore.NguenaNguefack@uqat.ca Hermine Lore Nguena Nguefack, MSc, Département des sciences de la santé, Université du Québec en Abitibi-Témiscamingue (UQAT), Rouyn-Noranda, Québec, Canada

© 2021 The Author(s). Published with license by Taylor & Francis Group, LLC.
This is an Open Access article distributed under the terms of the Creative Commons Attribution License (http://creativecommons.org/licenses/by/4.0/), which permits unrestricted use, distribution, and reproduction in any medium, provided the original work is properly cited.

### Investigation of cannabinoid CB1 receptor expression in spinal pain processing networks across sex and species

Jessica Parnell[a], Christopher Rudyk[a], Annemarie Dedek[a], Jeffrey Landrigan[a], Eve Tsai[b], and Michael Hildebrand[a]

[a]Carleton University, Neuroscience, Ottawa, Ontario, Canada; [b]Ottawa Hospital Research Institute, Neuroscience Program, Ottawa, Ontario, Canada

**Introduction/Aim**: There is preclinical and clinical evidence to suggest that cannabis, a potent cannabinoid, has potential analgesic properties. However, there is a gap in the literature with regards to cannabinoid receptor expression in the spinal cord across both sex and species, with almost nothing known in humans. Investigating mechanisms of cannabinoid signalling in the spinal cord will help determine whether this locus is involved in the analgesic properties of cannabis. We are therefore investigating the differential expression of the CB1 cannabinoid receptor across dorsal horn laminae and cell populations.

**Methods**: We use immunohistochemistry to examine the expression of CB1 receptors in the dorsal horn of spinal cord sections from male and female adult rats and humans. We collected human spinal cord samples from organ donors 1 to 3 hours post-aortic cross-clamping.

**Results**: We have successfully applied staining procedures from rats to fixed human tissue and find robust expression of CB1 in the human superficial dorsal horn. We are now quantifying the relative distribution of CB1 in the superficial dorsal horn (labelled with CGRP) compared to the deep dorsal horn. We are also quantifying the relative localization of CB1 to primary pain afferents (CGRP) versus spinal neurons (NeuN) versus astrocytes (GFAP). Active comparisons of CB1 expression are being made across both sex and species to identify where CB1 signalling may converge or diverge across these variables.

**Discussion/Conclusions**: The preferential expression of CB1 receptors in the superficial dorsal horn across both sex and species has significant implications for both the understanding and treatment of pain.

**CONTACT** Jessica Parnell jessicaparnell@cmail.carleton.ca

© 2021 The Author(s). Published with license by Taylor & Francis Group, LLC.
This is an Open Access article distributed under the terms of the Creative Commons Attribution License (http://creativecommons.org/licenses/by/4.0/), which permits unrestricted use, distribution, and reproduction in any medium, provided the original work is properly cited.

### Narrative Review of Nonpharmacological Strategies for Acute Procedural Pain in Hospitalized Preterm Infants

Ilana Shiff, Oana Bucsea, and Rebecca Pillai Riddell

York University, Clinical-Developmental Psychology, Toronto, Ontario, Canada

**Introduction/Aim**: Preterm pain is common in the Neonatal Intensive Care Unit (NICU), with multiple skin-breaking procedures occurring daily (Cruz et al., 2016). A large body of research has illustrated that repeated, unmanaged pain during the neonatal period can have significant detrimental effects on preterm infants' development (Valeri et al., 2015). Non-pharmacological strategies continue to be developed and evaluated in an effort to mitigate these effects. We present a narrative review of the updated literature, including a synthesis of key pieces of relevant published systematic reviews.

**Methods**: We searched multiple databases for relevant articles published before July 2020. Reference is also made to the published results from a 2015 Cochrane Review entitled "Non-pharmacological management of infant and young child procedural pain" and other relevant reviews. We present various strategies that require differing levels of caregiver (i.e., parents and health professionals) involvement during the enactment of the pain management strategy (minimal, moderate, extensive).

**Results**: The available evidence suggests that for preterm infants, non-nutritive sucking, facilitated tucking, swaddling, light reduction, multisensory bundle strategies and kangaroo care appear to be efficacious at reducing pain. Additionally, combining two non-pharmacological interventions as well as a non-pharmacological intervention with sucrose appear to be promising ways to manage preterm infant pain in the NICU.

**Discussion/Conclusions**: Research into non-pharmacological management of preterm pain is burgeoning, and should continue to be reviewed. Proximity to a caregiver before, during, and after a procedure is ideal practice, and caregivers should be included in pain management implementation when possible.

**CONTACT** Ilana Shiff ishiff@yorku.ca

© 2021 The Author(s). Published with license by Taylor & Francis Group, LLC.
This is an Open Access article distributed under the terms of the Creative Commons Attribution License (http://creativecommons.org/licenses/by/4.0/), which permits unrestricted use, distribution, and reproduction in any medium, provided the original work is properly cited.

### Gender Differences in Chronic Non-Cancer Pain Patients: findings from patients referred to Chronic Pain Management Program in NorthWestern Ontario

Hadi Shojaei[a], Arman Shojaei[b], and Delaram Shojaei[c]

[a]Northern Ontario School of Medicine, Physiatrist, Assistant professor, Thunder Bay; [b]York University, Ontario; [c]University of British Colombia

**Aim**: Do women and men perceive chronic pain differently? Physicians need this answer when they try to treat patients. Sex differences have been reported in the experience of pain and the prevalence of chronic pain conditions. This study aimed to identify whether sex differences in chronic pain management care exist for patients referred to the chronic pain clinic.

**Methods**: This retrospective study was conducted on 101 consecutive CNCP patients referred to CPMP (July to December 2018). Data collected included demographics, inciting event, pain characteristics; and baseline emotional and functional status obtained by validated instruments. Statistical analyses were conducted to assess whether relationships were statistically significant using the appropriate statistics (eg, *t*, F, $\chi^2$).

**Result**: Male/female ratio was 1:2.1 (p<0.005). Mean age for males was 57±16yrs. compared to females 52 ±15yrs. In regards to marital status, males were 31% vs 22% females were single and 53% vs 26 were married, respectively (p<0.05). Average pain duration in months was 23±14 (male) vs 17±14 (female) (p<0.05). Women reported a greater number of pain locations than men. 16% of males and 26% of females suffered from Non-dermatomal sensory deficits. No clinically meaningful differences were detected in BPI, emotional or functional status instruments between males and females.

**Discussion**: Females attended CPMP twice more than males and at younger age. While male patients had marital status twice as female patients (53% vs 26%), less females were single (22%), showing males have stronger family support than females. While CPMP had no gender priority policy for making appointment, this data showed that females tend to attend the program earlier than males, suggesting low pain threshold and tolerance among females. Likewise, female reported higher number of pain areas, showing that they are vulnerable to develop widespread or multi-site body pain. On the other hand, higher rate of NDSD showed that females' pain tend to involve the central nervous system more than the male's.

© 2021 The Author(s). Published with license by Taylor & Francis Group, LLC.
This is an Open Access article distributed under the terms of the Creative Commons Attribution License (http://creativecommons.org/licenses/by/4.0/), which permits unrestricted use, distribution, and reproduction in any medium, provided the original work is properly cited.

### Comparing task-specific sensitivity to physical activity measures among patients with knee osteoarthritis: preliminary analyses from a longitudinal cohort study

Anthony Teoli, Shawn Robbins, and Timothy Wideman

McGill University, School of Physical & Occupational Therapy, Montreal, Quebec, Canada

**Introduction/Aim**: Sensitivity to physical activity (SPA) is a novel measure quantifying the pain response to standardized physical activities or tasks. However, it is unclear which tasks are most appropriate to assess SPA in patients with knee osteoarthritis (KOA). This study aimed to compare baseline evoked-pain responses during five tasks and estimate responsiveness of task-specific SPA measures following an 8-week rehabilitation program, in patients with KOA.

**Methods**: Fifty-five participants (mean age=61 years, 39 females) with KOA underwent an 8-week, activity-based rehabilitation program. To determine SPA, participants rated their pain severity using a numeric rating scale (0-100) before and after five tasks: 30-Second Chair Stand (30CST), 40-Meter Fast-Paced Walk (40FPW), Timed-Up-and-Go (TUG), 6-Minute Walk Test (6MWT) and Step Test (ST). Task-specific SPA indices were generated by subtracting the pre-task pain from the post-task pain. Paired-samples t-tests evaluated whether the post-task pain was significantly greater than the pre-task pain for all tasks at baseline. Responsiveness of task-specific SPA measures following the 8-week rehabilitation program was assessed using effect sizes.

**Results**: The 30CST (5-point increase, p=0.007), 40FPW (9.5-point increase, p<0.001), 6MWT (14-point increase, p<0.001) and ST (19-point increase, p<0.001) elicited a significant increase in pain at baseline. Responsiveness following the rehabilitation program for the 40FPW-SPA measure was 0.29 (small). Responsiveness for remaining task-specific SPA measures was less than 0.2.

**Discussion/Conclusions**: The 40FPW may be most appropriate for assessing SPA in patients with KOA, as it elicited a significant increase in pain and the 40FPW-SPA measure was most responsive to change following the rehabilitation program.

### ORCID

Anthony Teoli http://orcid.org/0000-0003-0853-8406
Shawn Robbins http://orcid.org/0000-0002-8108-0561
Timothy Wideman http://orcid.org/0000-0003-1574-6967

© 2021 The Author(s). Published with license by Taylor & Francis Group, LLC.
This is an Open Access article distributed under the terms of the Creative Commons Attribution License (http://creativecommons.org/licenses/by/4.0/), which permits unrestricted use, distribution, and reproduction in any medium, provided the original work is properly cited.

### Investigation of spontaneous NMDA receptor-mediated synaptic responses in lamina I spinal neurons across sex and species.

Emine Topcu[a], Annemarie Dedek[b], Christopher Dedek[c], Eve Tsai[d], and Mike Hildebrand[e]

[a]Carleton University, Neuroscience, Ottawa, Ontario, Canada; [b]Ottawa Hospital Research Institute, Ottawa, Ontario, Canada; [c]Ottawa Hospital Research Institute, Neuroscience Program, Ottawa, Ontario, Canada; [d]University of Ottawa, Brain and Mind Research Institute, Ottawa, Ontario, Canada; [e]The Ottawa Hospital, Department of Surgery, Division of Neurosurgery, Ottawa, Ontario

**Introduction/Aim**: Lamina I projection neurons are involved in projection of nociception signals to supraspinal regions, leading to perception of pain. Ionotropic glutamate NMDA receptors (NMDARs) regulate synaptic plasticity and excitability in lamina I neurons. The NMDAR is a heterotetramer protein and its subunit composition impacts the kinetics of the receptor and associated physiological and pathological signaling. In this study, we analyzed spontaneously occurring miniature excitatory post synaptic currents (mEPSC) to assess the differential contribution of NMDAR subunits to lamina I synaptic responses across sex and species.

**Methods**: Voltage clamp electrophysiology was performed on lamina I neurons of female and male adult rats, as well as female and male human spinal cords. We collected human spinal cord samples from male and female organ donors 1 to 3 hours post-aortic cross-clamping. Using a non-biased selection criterion, we detected and aligned all mEPSC traces recorded at +60 mV. Individual synaptic responses were placed in different bins, based on their differential decay rates. Both the peak amplitude and decay constants (through single exponential fitting) were calculated for individual synaptic events.

**Results**: The mEPSCs observed in both rats and humans shows a wide range of heterogeneity in kinetics and amplitude, with larger amplitude NMDAR responses in humans versus rats. This heterogeneity in synaptic NMDAR properties is also observed between individual synaptic responses for a given lamina I neuron.

**Discussion/Conclusions**: The heterogeneity in synaptic NMDAR kinetics and amplitude across species and sex has important implications for understanding molecular mechanisms of pain processing in the spinal cord.

© 2021 The Author(s). Published with license by Taylor & Francis Group, LLC.
This is an Open Access article distributed under the terms of the Creative Commons Attribution License (http://creativecommons.org/licenses/by/4.0/), which permits unrestricted use, distribution, and reproduction in any medium, provided the original work is properly cited.

### The Epidemiology of Chronic Pain in Children and Adolescents: A Systematic Review Update

Perri R. Tutelman[a], Christine T. Chambers[b], Charlotte L. Langley[c], Jennifer A. Parker[c], Brittany Cormier[c], Darlene Chapman[d], G. Allen Finley[e], Gareth T. Jones[f], Gary J. Macfarlane[g], and Justina Marianayagam[h]

[a]Dalhousie University, Department of Psychology and Neuroscience, Halifax, NS, Canada; [b]Dalhousie University, Departments of Psychology and Neuroscience and Pediatrics, Halifax, NS, Canada; [c]IWK Health Centre, Centre for Pediatric Pain Research, Halifax, NS, Canada; [d]IWK Health Centre, Library Services, Halifax, NS, Canada; [e]Dalhousie University, Departments of Anesthesia and Psychology, Halifax, NS, Canada; [f]University of Aberdeen, Epidemiology Group and Aberdeen Centre for Arthritis and Musculoskeletal Health, Aberdeen, Aberdeen, UK; [g]University of Aberdeen, Epidemiology Group and Aberdeen Centre for Arthritis and Musculoskeletal Health, Aberdeen, Aberdeen, UK; [h]Northern Ontario School of Medicine, Thunder Bay, ON, Canada

**Introduction/Aim**: Chronic pain is a common childhood problem. The last comprehensive systematic review estimating the prevalence of chronic pain in children and adolescents was published in 2011. Since then, the literature has grown substantially. Thus, the objective of this review is to provide updated prevalence estimates of chronic pain, and to examine sociodemographic and psychosocial factors related to the prevalence of chronic pain in children and adolescents.

**Methods**: Comprehensive searches were implemented in four scientific databases. Eligible articles were observational studies published in English between 2009 and 2020 reporting population-based estimates of chronic, non-disease-related pain prevalence in children or adolescents (≤ 19 years).

**Results**: The search yielded 12,426 citations. After deletion of duplicates, 10,902 titles and abstracts were screened for eligibility, and 236 selected for further review. The prior review estimated the median prevalence proportion of headache at 23%. The median prevalence proportions of other types of chronic pain (e.g., abdominal pain, back pain, musculoskeletal pain, and pain combinations) ranged from 11-38%. Chronic pain was more prevalent in girls and lower socioeconomic status was associated with higher pain prevalence. This review uses median prevalence proportions and/or meta-analysis (based on study heterogeneity) to pool the prevalence of various chronic pain types. Meta-regression is used to examine the relationship between socio-demographic and psychosocial factors and chronic pain across studies.

**Discussion/Conclusions**: Chronic pain is prevalent in children and adolescents. This work provides an updated understanding of the current burden of pediatric chronic pain, which will inform allocation of clinical resources for this population.

**ORCID**

Perri R. Tutelman http://orcid.org/0000-0001-6013-7402
Christine T. Chambers http://orcid.org/0000-0002-7138-916X
Jennifer A. Parker http://orcid.org/0000-0001-9900-4703
G. Allen Finley http://orcid.org/000-0003-4579-7749
Gareth T. Jones http://orcid.org/0000-0003-0016-7591
Gary J. Macfarlane http://orcid.org/0000-0003-2322-3314

**CONTACT** Perri Tutelman PTutelman@dal.ca

© 2021 The Author(s). Published with license by Taylor & Francis Group, LLC.
This is an Open Access article distributed under the terms of the Creative Commons Attribution License (http://creativecommons.org/licenses/by/4.0/), which permits unrestricted use, distribution, and reproduction in any medium, provided the original work is properly cited.

### Age dependent modification of intensity and time course of the pain peak sensation

Matthieu Vincenot[a], Monica Sean[b], Louis Gendron[b], Serge Marchand[a], and Guillaume Léonard[c]

[a]Université de Sherbrooke, Faculté de médecine et des sciences de la santé, Sherbrooke, Québec, Canada; [b]Université de Sherbrooke, Institut de pharmacologie et physiologie, Sherbrooke, Québec, Canada; [c]Université de Sherbrooke, École de réadaptation, Québec, Canada

**Introduction/Aim**: Recent evidence suggests that the initial peak pain phase observed during a continuous tonic heat pain test is significantly reduced in elderly individuals compared to young adults, presumably reflecting age-related alterations in the nociceptive circuitry. The aim of the present study was to expand the scope of research on these age-related changes by adding a middle-aged group to the habitual intergroup contrast of young and elderly adults.

**Methods**: 346 healthy participants (young (18-40); n=128, middle-aged (41-60); n =114 and elderly (61-76) n=104) participated in the study. Experimental heat pain was evoked on the forearm using a thermode test (2-minute stimulation at a constant, individually-adjusted temperature). The amplitude of the peak pain response (highest pain score observed during the first 30 seconds) and its time course (time needed to reach peak pain) was calculated for each participant and compared between the three age-groups using a one-way ANOVA.

**Results**: The analyses showed that 1) the amplitude of the peak pain was significantly lower for elderly individuals compared to young ($p$ <.01) but not to middle aged individuals ($p$ =.09), and 2) that the time needed to reach peak pain was shorter for young individuals compared to both middle-aged ($p$ <.01) and elderly individuals ($p$ <.01). No significant difference was observed between young and middle-aged individuals for the peak pain amplitude ($p$ =.09), and between elderly and middle-aged individuals for the time course ($p$= .34).

**Discussion/Conclusions**: These results suggest that some changes in nociceptive systems seems to occur earlier than previously reported.

**ORCID**

Guillaume Léonard http://orcid.org/0000-0002-3625-3072

© 2021 The Author(s). Published with license by Taylor & Francis Group, LLC.
This is an Open Access article distributed under the terms of the Creative Commons Attribution License (http://creativecommons.org/licenses/by/4.0/), which permits unrestricted use, distribution, and reproduction in any medium, provided the original work is properly cited.

### Identification of the neural network for conditioned analgesia

Chulmin Cho[a], Vassilia Michailidis[b], Simran Rehal[a], Ahmed Aldarraji[b], Fiona Ramnaraign[b], Areej Fatima[c], Natalia Dziekonski[c], Hyun Been Park[d], Batul Presswala[c], and Loren J. Martin[c]

[a]University of Toronto, Psychology, Toronto, ON, Canada; [b]University of Toronto, Cell Systems Biology, Toronto, ON, Canada; [c]University of Toronto, Psychology, Toronto, ON; [d]University of Toronto, Molecular Genetics, Toronto, ON, Canada

**Introduction/Aim:** Chronic pain can be interpreted as a maladaptive pain memory and is susceptible to alterations through learning that includes conditioning. In support, conditioned analgesia describes a phenomenon where an inert treatment triggers pain relief. The objective of the study was to identify the neural network(s) in the brain responsible for conditioned analgesia using a mouse model of chronic neuropathic pain.

**Methods:** Mechanical pain thresholds were measured in 6-8wk old male CD-1 mice, before and following spared nerve injury (SNI). The SNI mice then underwent a four-day conditioning phase where contextual and tactile stimuli were coupled with an unconditioned drug stimulus (morphine, 10mg/kg). Following the conditioning period, the SNI mice were administered either saline or an opioid receptor antagonist. The neural activity was mapped in the brain by probing for c-fos expression using immunohistochemistry.

**Results:** Saline administration following pharmacological conditioning induced analgesia comparable to that of morphine, which was reversed by naloxone. Systemic administration of antagonists for opioid receptor subtypes mu, delta and kappa demonstrated that inhibition of mu-opioid receptor (MOR) blocked saline-induced analgesia, suggesting that opioid-mediated placebo analgesia occurs via MOR. Immunohistochemical analyses revealed significant changes of c- fos expression in the prefrontal cortex and brain regions salient to sensory-discriminative and affective aspects of pain processing.

**Discussion/Conclusions:** We demonstrate a novel animal model of conditioned analgesia within the context of chronic neuropathic pain. This is accompanied by corresponding changes of neuronal activities in the brain, warranting further investigations in order to elucidate the functional neural network(s) that drives conditioned analgesia.

© 2021 The Author(s). Published with license by Taylor & Francis Group, LLC.
This is an Open Access article distributed under the terms of the Creative Commons Attribution License (http://creativecommons.org/licenses/by/4.0/), which permits unrestricted use, distribution, and reproduction in any medium, provided the original work is properly cited.

### Prolonged hyperalgesia induced by optogenetic activation of TrpV1+ nociceptors using direct spinal light delivery

Maham Zain, Laura A. Bennett, Jerry Li, and Robert P. Bonin

University of Toronto, Leslie Dan Faculty of Pharmacy, Toronto, Ontario, Canada

**Introduction/Aim**: The recent strides in optogenetics has spurred research into the neural basis of pain *in vivo* and in freely moving animals. We have developed and characterized a method for the delivery of light to the spinal dorsal horn that is suitable for optogenetic studies in freely behaving mice. Using this approach, we activated TrpV1+ nociceptors expressing channelrhodopsin (ChR2) to investigate how the activation of these sensory fibers can both acutely modulate behaviour and induce hyperalgesia.

**Methods**: A ceramic ferrule was surgically implanted in the lumbar vertebrae of wildtype (WT) mice and mice expressing ChR2 in TrpV1+ afferents (TrpV1-ChR2). A fiber optic patch cable connected the implanted lens to a blue LED light source for stimulation of the central terminals of the TrpV1+ primary afferents. Possible adverse effects of this surgery were studied using immunohistochemical assessment of spinal cord glial activation, open field and rotarod behavioral assays. Mechanical sensitivity was assessed using von Frey filaments and thermal sensitivity using a thermal gradient assay to measure thermal preference.

**Results**: We observed that the surgical technique did not result in any changes in evoked motor function, spontaneous locomotion or mechanosensitivity. In addition, there was no evidence of astrocyte or microglia activation in the spinal cord after surgery. Delivery of blue light through the implant was found to result in robust nocifensive behaviors in the TrpV1-ChR2 mouse line. After suprathreshold stimulation of TrpV1-ChR2 at 2-Hz for 10 minutes, mice exhibited robust mechanical hypersensitivity which lasted approximately 2 weeks. Similar stimulation in implanted WT mice did not result in mechanical hypersensitivity. Notably, thermal preferences of the mice were not affected by either suprathreshold 2-Hz stimulation or acutely delivered subthreshold stimulation. Finally, delivery of light at a subthreshold intensity that did not acutely produce nocifensive behaviors produced progressively more pronounced nocifensive behaviors after prolonged stimulation.

**Discussion/Conclusions**: Optogenetic activation of TrpV1+ afferents produced a surprisingly long-lasting mechanical hypersensitivity. This suggests that optogenetically-induced hyperalgesia may mechanistically differ from the shorter duration hypersensitivity induced by capsaicin activation of peripheral TrpV1+ afferents. In addition, persistent subthreshold stimulation eventually resulted in an emergence of nocifensive behaviors over the time course of the stimulation, alluding to potential wind-up. Further work will investigate the mechanisms of these behavioral changes.

© 2021 The Author(s). Published with license by Taylor & Francis Group, LLC.
This is an Open Access article distributed under the terms of the Creative Commons Attribution License (http://creativecommons.org/licenses/by/4.0/), which permits unrestricted use, distribution, and reproduction in any medium, provided the original work is properly cited.

### Exploring Circumstances and Consequences of Falls in Adults Living with Chronic Pain

Corinna Easton[a], Meg Henderson[a], Marcia Finlayson[a], Mary Anne Good[b], Rosemary Wilson[c], Scott Duggan[b], and Etienne J. Bisson[b]

[a]Queen's University, School of Rehabilitation Therapy, Kingston, Ontario, Canada; [b]Kingston Health Sciences Centre-Hotel Dieu Hospital site, Chronic Pain Clinic, Kingston, Ontario, Canada; [c]Queen's University, School of Nursing, Kingston, Ontario, Canada

**Introduction/Aim**: Adults with chronic pain have a high prevalence of falls. We aimed to determine the circumstances and consequences of falls in this population.

**Methods**: Adults with chronic pain who reported a fall in the previous year were invited to complete an online survey. Information collected included fall history, circumstances and consequences of the most recent fall, medical history, and pain condition.

**Results**: Participants (N=123; 93% woman; mean age=52.7(11.8) with diverse pain conditions reported a total of 618 falls in the previous year (median=3 falls; IQR=2-4; 75% with multiple falls). 73% had chronic pain before experiencing falls whereas 3% identified falling as related to chronic pain onset. 81% reported falls increase pain most of the time and 86% identified that falls impact their pain management. More than half of participants' most recent falls happened indoors while walking (38%) or negotiating stairs (15%). Mobility issues (e.g. loss of balance, tripping) were the most reported causes (81%), but symptoms such as fatigue and environmental causes were also commonly reported. Three quarters of participants acquired an injury from their most recent fall with 15% reporting a fracture. A third reported decrease confidence and independence to perform most ADLs following their most recent fall.

**Discussion**: Since falls are common and the risks of injurious falls are high, falls prevention in this population is critical to optimize pain management and ensure patient safety. Our findings suggest that preventive measures should mainly focus on physical functioning, but also management of related symptom such as fatigue.

### ORCID

Corinna Easton http://orcid.org/0000-0002-8795-6341
Marcia Finlayson http://orcid.org/0000-0002-1774-4810
Rosemary Wilson http://orcid.org/0000-0003-3262-243X

© 2021 The Author(s). Published with license by Taylor & Francis Group, LLC.
This is an Open Access article distributed under the terms of the Creative Commons Attribution License (http://creativecommons.org/licenses/by/4.0/), which permits unrestricted use, distribution, and reproduction in any medium, provided the original work is properly cited.

### Analysing Medical Clerk Perceptions of Chronic Pain-Centered Curricula in Undergraduate Medical Education

Myles Benayon and Lekhini Latchupatula

McMaster University, Medicine, Hamilton, Ontario, Canada

**Introduction/Aim**: The Canadian Pain Task Force has highlighted inadequacies in pain curricula in undergraduate medical education (UGME). This is disastrous considering that of the 1 in 5 Canadians who suffer from chronic pain (CP), 40-80% are misdiagnosed. This study's objective is to quantify McMaster medical clerks' perception of CP learning opportunities and confidence in CP-related pathologies, to promote changes to the existing UGME program.

**Methods**: A Google Forms survey was distributed through email and social media to 618 McMaster undergraduate pre-clerks and clerks in all three years of training. Data were assessed with descriptive statistics.

**Results**: 168 (27%) responses were collected from pre-clerks (N=53), junior clerks (N=62), and senior clerks (N=53). 140 (83%) students rated their satisfaction with the pre-clerkship CP curriculum as 3 or below on a scale ranging from 1 (None at All) to 5 (Very Satisfied). Additionally, senior clerks rated their knowledge of CP and pain management as 1.76 before medical school and currently only rate it as 3.13 on a scale of 1 (Very Little) to 5 (Extremely High). Almost all (98%) senior clerks foresee their medical practice involving CP patients.

**Discussion/Conclusions**: These results revealed the existing deficiencies in the current pain-specific McMaster UGME. Changes to curricula can be made by improving online CP teaching modules and virtual multi-disciplinary team workshops. We hope to administer this survey to all medical schools in Canada and improve UGME nationwide, so future physicians can be better equipped to address the prevalence of misdiagnosis among CP patients.

© 2021 The Author(s). Published with license by Taylor & Francis Group, LLC.
This is an Open Access article distributed under the terms of the Creative Commons Attribution License (http://creativecommons.org/licenses/by/4.0/), which permits unrestricted use, distribution, and reproduction in any medium, provided the original work is properly cited.

### Association between pain acceptance and opioid misuse in patient with chronic pain

Alice Bruneau[a], Maria Verner[b], Amanda Sirois[b], Kathryn Scott[c], Leah Frimerman[d], Catherine Fournier[b], Jordi Perez[e], Yoram Shir[e], and Marc O. Martel[b]

[a]McGill University, Experimental Medicine, Montreal, Quebec, Canada; [b]McGill University, Dentistry, Montreal, Quebec, Canada; [c]McGill University, Physiology, Montreal, Quebec, Canada; [d]SUNY Downstate Health Sciences University, Medicine, New York, New York, United States; [e]McGill University Health Centre, Alan Edwards Pain Management Unit, Montreal, Canada

**Introduction/Aim**: Pain acceptance is known to be an important determinant of pain outcomes in patients with chronic pain. Pain acceptance could also influence opioid-related outcomes, but little work has been done in this area. The first objective of this study was to examine the association between pain acceptance and prescription opioid misuse among patient with chronic pain. We then examined if patients' daily levels of pain intensity, negative affect, or opioid craving mediated this association.

**Methods**: Patients living with chronic pain and using prescription opioids ($n = 103$) were included in this observational longitudinal study. Patients first completed questionnaires assessing a host of demographic, opioid, and psychological variables, including pain acceptance. Patients then completed daily diaries over 14 consecutive days about pain, negative affect, and opioid craving. Patients also reported if they engaged in certain types of opioid misuse behaviours.

**Results**: Results indicated that greater pain acceptance was associated with less frequent opioid misuse, $r_s = -.24$, $p < .05$. Greater pain acceptance was also associated with lower daily levels of pain intensity, negative affect, and opioid craving (all p's $< 0.05$). Results of a mediation analysis subsequently revealed that negative affect and opioid craving mediated the relationship between pain acceptance and opioid misuse ($p < 0.05$).

**Discussion/Conclusions**: Our findings suggest that greater pain acceptance is associated with decreased negative affect and opioid craving. In turn, this could lead to less frequent opioid misuse behaviours. Psychological interventions based on pain acceptance might be beneficial for patients with pain using opioids.

### ORCID

Alice Bruneau http://orcid.org/0000-0002-1972-4864
Maria Verner http://orcid.org/0000-0002-3498-0751

© 2021 The Author(s). Published with license by Taylor & Francis Group, LLC.
This is an Open Access article distributed under the terms of the Creative Commons Attribution License (http://creativecommons.org/licenses/by/4.0/), which permits unrestricted use, distribution, and reproduction in any medium, provided the original work is properly cited.

### Examining the role of outcome expectations and working alliance in predicting pain outcomes following group-based psychotherapy for chronic pain management

Katherine M. Fretz[a], Alison Crawford[a], Etienne J. Bisson[b], Rebecca McDermott[b], Katie Root-Clarke[b], Jennifer Bossio[b], Hannah Marchand[b], and Dean A. Tripp[c]

[a]Queen's University, Department of Psychology, Kingston, ON, Canada; [b]Kingston Health Sciences Centre-Hotel Dieu Hospital site, Chronic Pain Clinic, Kingston, Ontario, Canada; [c]Queen's University, Departments of Psychology, Anesthesiology, & Urology, Kingston, Ontario, Canada

**Introduction/Aim**: Outcome expectations (i.e., expectations about treatment benefits) and working alliance (i.e., client-therapist bond, agreement on goals/tasks) predict favorable treatment results. Since the literature has focused on general psychotherapy populations, this study examined whether such variables interact to predict outcomes in an adult chronic pain sample.

**Methods**: Sixty-six participants were included in the analyses. Treatment involved eight, manualized sessions of group psychotherapy at an interdisciplinary pain clinic. Participants rated their outcome expectations pre-treatment, working alliance at each session (averaged across sessions for analyses), and pain severity and pain interference post-treatment. Participants' last observed scores were carried forward if they discontinued treatment. Two sets of moderation models were performed, with working alliance and outcome expectations as the moderator, respectively. Each outcome's baseline scores were controlled for.

**Results**: Working alliance moderated the relationship between outcome expectations and pain severity, but not pain interference. Higher expectations were related to greater pain severity post-treatment only among those rating a relatively lower working alliance (*b*s (regression weights) > 0.19, *p*-values < .05). Outcome expectations moderated the relationship between working alliance and pain severity, but not pain interference. Higher alliance was related to lower pain severity post-treatment only among those with relatively high expectations (*b*s < -0.05, *p*-values < .05).

**Discussion/Conclusions**: Working alliance during treatment related more to positive outcomes when participants had higher initial expectations, but higher expectations related more to worse outcomes when alliance suffered. Clinicians should help chronic pain patients set positive yet reasonable outcome expectations for treatment and foster strong working alliances.

© 2021 The Author(s). Published with license by Taylor & Francis Group, LLC.
This is an Open Access article distributed under the terms of the Creative Commons Attribution License (http://creativecommons.org/licenses/by/4.0/), which permits unrestricted use, distribution, and reproduction in any medium, provided the original work is properly cited.

### Shared genetic risk factors in pain conditions and psychopathologies

Katerina Zorina-Lichtenwalter, Carmen Bango, Marta Čeko, Naomi P. Friedman, Matthew C. Keller, Lukas Van Oudenhove, Subrata Paul, and Tor D. Wager

[a]University of Colorado Boulder, Institute of Cognitive Science and Institute for Behavioral Genetics, Boulder, CO, USA; [b]Dartmouth College, Department of Psychological and Brain Sciences, Hanover, NH, USA; [c]KU Leuven, Department of Chronic Diseases, Metabolism, and Ageing, Leuven, Belgium; [d]University of Colorado Boulder, Institute for Behavioral Genetics, Boulder, CO, USA

**Introduction/Aim**: Chronic pain and psychological conditions show substantial genetic and phenotypic correlations. Correspondingly, they share symptoms, behavioural patterns, and brain mechanisms. Our goal for this study is to find and characterise the shared genetic risk components underlying these conditions.

**Methods**: Using the UK Biobank dataset, we selected chronic conditions marked by persistent pain and spanning numerous body sites and suspected etiologies. We ran a genome-wide association study (GWAS) on each condition and combined association statistics with publicly available GWAS summary statistics for psychological conditions to estimate genetic correlations among them. Next, we used the genomic structural equation modeling (gSEM) framework to perform factor analysis, to be followed by GWAS on extracted factors to identify single nucleotide polymorphisms (SNPs) associated with genetic predisposition to multiple forms of pathology. Additionally, we performed a factor analysis on the phenotypes of these conditions.

**Results**: Preliminary gSEM results show evidence of a bifactor structure, with a prominent general factor explaining most of the shared genetic variance and three specific factors with significant loadings from gastrointestinal, craniofacial, and musculoskeletal disorders. The bifactor model outperforms the model with three specific correlated factors.

**Discussion/Conclusions**: The correlation structure of the examined conditions suggests common pathophysiological mechanisms for gastrointestinal, craniofacial, and musculoskeletal conditions. Additionally, these etiologically distinct conditions manifesting in different body sites appear to share a genetic risk factor, which explains most of their common genetic variance. These findings contibute evidence for chronic pain as a *sui generis* condition, whose recognition as such may better inform care for chronic pain patients.

© 2021 The Author(s). Published with license by Taylor & Francis Group, LLC.
This is an Open Access article distributed under the terms of the Creative Commons Attribution License (http://creativecommons.org/licenses/by/4.0/), which permits unrestricted use, distribution, and reproduction in any medium, provided the original work is properly cited.

### What did three Statistics Canada Surveys tell us about the relationship between physical activity and chronic back pain in adults?

A Angarita Fonsseca[a], C Trask[b], P Pahwa[c], H Herman[d], D Fuller[e], and B Bath[f]

[a]University of Saskatchewan, Saskatoon, Canada; Département des sciences de la santé, Université du Québec en Abitibi-Témiscamingue (UQAT), Rouyn-Noranda, Québec, Canada; Faculté de médecine, Université de Montréal, Montréal, Québec, Canada; Universidad de Santander, Bucaramanga, Colombia; [b]Canadian Centre for Health and Safety in Agriculture, College of Medicine, University of Saskatchewan; Division of Ergonomics, School of Engineering Sciences in Chemistry, Biotechnology and Health, KTH Royal Institute of Technology, Stockholm, Sweden; [c]Department of Community Health and Epidemiology, College of Medicine, University of Saskatchewan, Saskatoon, Saskatchewan, Canada; [d]Faculty of Kinesiology & Health Studies, University of Regina, Regina, Saskatchewan, Canada; [e]School of Human Kinetics and Recreation, Canada Research Chair iFfn Population Physical Activity, Memorial University, St. John's, Newfoundland and Labrador, Canada; [f]School of Rehabilitation Science, College of Medicine, University of Saskatchewan, Saskatoon, Saskatchewan, Canada

**Introduction/Aim**: Although physical activity (PA) might be a factor in managing or preventing chronic back pain, the evidence supporting this hypothesis is inconclusive. This study aimed to 1) describe time trends in chronic back problems (hereafter CBP) prevalence by PA levels; 2) investigate the association between previous PA and subsequent CBP adjusted for previous CBP and other covariates and 3) explore the relationship between meeting the Canadian PA guidelines (MCPAG -Total PA) and CBP using either accelerometer-based or self-reported PA measures in Canadians adults.

**Methods**: 1) In a serial cross-sectional study using the 2007-to-2014 cycles of the Canadian Community Health Survey [CCHS], annual CBP prevalence by transportation and leisure-time PA [LTPA] levels were obtained. Joinpoint regressions were applied. 2) Using the longitudinal biannually National Population Health Survey [NPHS], LTPA levels, utilitarian walking or cycling were derived. Generalized estimating equations transition models [time-lagged] were applied. 3) Using the 2014-2017 Canadian Health Measures Surveys [CHMS], two binary logistic regression models were fitted: one containing self-reported MCPAG and another containing accelerometer-based MCPAG.

**Results**: CCHS showed a stable overall CBP prevalence (≈20%) and higher in people classified as inactive. NPHS showed that prior LTPA and CBP were associated with reporting CBP two years later [active versus inactive OR=0.89, 95%CI 0.8, 0.95]. CHMS showed that MCPAG was not associated with CBP, whether PA was measured by accelerometer or questionnaire.

**Conclusions**: CBP were related to specific domains of PA rather than total PA. There was an association between CBP and PA when temporality was considered.

© 2021 The Author(s). Published with license by Taylor & Francis Group, LLC.
This is an Open Access article distributed under the terms of the Creative Commons Attribution-NonCommercial License (http://creativecommons.org/licenses/by-nc/4.0/), which permits unrestricted non-commercial use, distribution, and reproduction in any medium, provided the original work is properly cited.

### Long-term Safety and Tolerability of Atogepant Following Once Daily Dosing Over 1 Year for the Preventive Treatment of Migraine

Messoud Ashina[a], Stewart Tepper[b], Uwe Reuter[c], Andrew Blumenfeld[d], Susan Hutchinson[e], Jing Xia[f], Rosa Miceli[g], Lawrence Severt[h], Michelle Finnegan[i], and Joel Trugman[j]

[a]Danish Headache Center, Neurology, Rigshospitalet Glostrup, University of Copenhagen, Denmark; [b]Dartmouth Geisel School of Medicine, Neurology, Hanover, NH, USA; [c]Charité University Hospital, Neurology, Berlin, Germany; [d]Headache Center of Southern California, Carlsbad, CA, USA; [e]Orange County Migraine and Headache Center, Irvine, CA, USA; [f]AbbVie, Biostatistics, Madison, NJ, USA; [g]AbbVie, Clinical Development, Madison, NJ, USA; [h]; [i]; [j]

**Introduction/Aim**: Atogepant is an oral, small molecule, CGRP receptor antagonist in development for the preventive treatment of migraine. We evaluated the safety and tolerability of atogepant over 52 weeks.

**Methods**: Multicenter, open-label trial (NCT03700320). Adults with migraine were randomized 5:2 to atogepant 60 mg once-daily or oral standard-of-care migraine prevention medicine. The primary objective was to assess the safety and tolerability of atogepant. Standard-of-care was included to primarily help contextualize hepatic safety data.

**Results**: The trial included 744 randomized participants (n=546 atogepant) with 739 in the safety population (n=543 atogepant). Adverse events (AEs) were reported by 67.0% of atogepant-treated participants; 18.0% reported AEs that were considered treatment-related. Most commonly reported AEs (≥5% of participants) were upper respiratory tract infection (10.3%), constipation (7.2%), nausea (6.3%), and urinary tract infection (5.2%) following atogepant treatment. Serious AEs were reported by 4.4% of atogepant-treated participants; no event was seen in >1 participant and no event was considered treatment-related. Two deaths were reported in atogepant-treated participants (homicide victim and a group A beta-hemolytic streptococcal sepsis [toxic shock syndrome]); both were considered not related to atogepant. Discontinuation due to AEs was 5.7% following atogepant treatment. Cases of alanine aminotransferase/aspartate aminotransferase levels ≥3 times the upper limit of normal were reported for 2.4% of atogepant-treated participants (n=13/531) and 3.2% for standard-of-care (n=6/190). No cases of potential Hy's Law were reported.

**Discussion/Conclusions**: Long-term, once daily use of atogepant 60 mg for the preventive treatment of migraine over 1 year was safe and well-tolerated with no safety concerns identified.

**CONTACT** Messoud Ashina ashina@dadlnet.dk

© 2021 The Author(s). Published with license by Taylor & Francis Group, LLC.
This is an Open Access article distributed under the terms of the Creative Commons Attribution License (http://creativecommons.org/licenses/by/4.0/), which permits unrestricted use, distribution, and reproduction in any medium, provided the original work is properly cited.

### A randomized clinical trial of perioperative clinical hypnosis' effects on heart-rate variability in surgical oncology patients

Muhammad Abid Azam[a,b], Aliza Weinrib[a,b], Brittany Rosenbloom[a,b], Max Slepian[a,b], Sonalben Thaker[a], Andrea Aternali[a,b], Hance Clarke[a], and Joel Katz[a,b]

[a]Toronto General Hospital, Department of Anesthesia and Pain Management, Toronto, ON, Canada; [b]York University, Department of Psychology, Toronto, ON, Canada

**Introduction/Aim**: Clinical hypnosis is a recommended adjunct for post-surgical pain management. Evidence is needed to understand its benefits for post-surgical recovery. It was hypothesized that surgical oncology patients randomized to receive perioperative clinical hypnosis (CH) would demonstrate greater heart-rate variability (HRV) during rest and relaxation at a 1-month post-surgery assessment compared to a treatment-as-usual group (TAU), reflecting the protective effects of clinical hypnosis on vagal activity.

**Methods**: After REB approval and informed consent were obtained, 92 participants were randomized to receive CH (n=45) or TAU (n=47). CH participants received a CH session before surgery and during the post-surgical in-hospital stay, with suggestions for pain relief. HRV was assessed during rest (5 min) and relaxation (10 min) before and 1-month after surgery. Pain intensity was obtained using a 0-10 numeric rating scale before, and 1-week and 1-month after surgery.

**Results**: One month after surgery, HRV was significantly higher in CH group during rest and relaxation (both p<0.05, d=0.73) than TAU group. By contrast, rest and relaxation HRV significantly decreased from pre- to 1-month post-surgery for the TAU (both p<0.001, d=0.48) but not the CH group. Pain intensity significantly increased from pre-surgery to 1-week post-surgery (p<0.001, d=0.50), and decreased from 1-week to 1-month post-surgery (p=0.005, d=0.21) for all participants.

**Discussion/Conclusions**: The results suggest that CH prevents the deleterious effects of surgery on HRV by preserving pre-operative vagal activity. The TAU group display an expected decrease in HRV in the absence of CH.

### ORCID

Muhammad Abid Azam http://orcid.org/0000-0002-6016-1274
Joel Katz http://orcid.org/0000-0002-8686-447X

© 2021 The Author(s). Published with license by Taylor & Francis Group, LLC.
This is an Open Access article distributed under the terms of the Creative Commons Attribution License (http://creativecommons.org/licenses/by/4.0/), which permits unrestricted use, distribution, and reproduction in any medium, provided the original work is properly cited.

### RCT Evaluating a Smartphone-Based Mindful Breathing Task's Effects on Heart-Rate Variability in Undergraduates with Symptoms of Chronic Pain, Depression, and Anxiety

Muhammad Abid Azam, Vered Latman, Helia Ghazinejad, Blake Boehme, Amir Zarie, Myra Massey, and Joel Katz

York University, Department of Psychology, Toronto, ON, Canada

**Introduction/Aim**: Mindfulness meditation (MM) is often used for individuals with pain, depression, and anxiety, but it can be challenging without proper training. The mindfulness meditation app (MMA) is a smartphone-based training app developed to aid mindful breathing. It was hypothesized that heart-rate variability (HRV) would be higher in individuals with chronic pain (CP) and those with symptoms of depression and anxiety (DA) assigned to practice MM with the app than those without the app.

**Methods**: 213 participants were classified into study groups of CP (n=58), DA (n=64) and sub-criteria (SC; n=91). Participants were randomly assigned to an MMA+ condition or to MM without the app (MMA–) after a brief stress-induction procedure. MMA+ participants practiced mindful breathing and pressed 'breath' or 'other' buttons at the sound of audio tones if their awareness was on breathing or another experience, respectively. HRV was obtained during rest (5 min), stress-induction (5 min), and meditation conditions (12 min).

**Results**: The effect of the app condition during the MM phase was significant for the CP group (p=0.01), but not the DA (p=0.10) or SC (p=0.63) groups. Further examination showed, contrary to the hypothesis, that HRV was significantly higher in the MMA- condition compared to the MMA+ for CP (p=0.001, d=0.67).

**Discussion/Conclusions**: MMA+ did not promote vagal activation in participants with chronic pain and participants scoring at or above clinical thresholds for anxiety and depression. Future research is needed to design effective training tools for MM, as the MMA's audio tones may have served to distract from or interfered with MM practice.

### ORCID

Muhammad Abid Azam http://orcid.org/0000-0002-6016-1274
Joel Katz http://orcid.org/0000-0002-8686-447X

© 2021 The Author(s). Published with license by Taylor & Francis Group, LLC.
This is an Open Access article distributed under the terms of the Creative Commons Attribution License (http://creativecommons.org/licenses/by/4.0/), which permits unrestricted use, distribution, and reproduction in any medium, provided the original work is properly cited.

### Chemotherapy-induced peripheral neuropathy patient reported outcomes: using a think-aloud protocol to evaluate content validity

Maud Bouffard[a], Sarah Béland[a], Camille Côté[b], Julie Lemieux[c], Robert H Dworkin[d], Lucia Gagliese[e], Cindy Shobbrook[f], Diane Tapp[g], Josée Savard[g], Michèle Aubin[g], Anne Dionne[g], Sophie Lauzier[g], Philip Jackson[h], Bruno Gagnon[i], Pierre Gagnon[j], Jennifer Gewandter*[k], and Lynn R Gauthier*[l]

[a]Research Centre of the CHU de Quebec, Oncology Division, Quebec City, Quebec, Canada; [b]Université Laval, Faculty of Medicine, Quebec City, Quebec, Canada; [c]CHU de Quebec, Centre des maladies du sein Deschênes-Fabia, Hôpital du Sainte Sacrement, Quebec City, Quebec, Canada;

[d]University of Rochester Medical Center, School of Medicine and Dentistry, Rochester, New York; [e]York University, School of Kinesiology and Health Science, Toronto, Ontario, Canada; [f]Waterloo-Wellington Hospice Palliative Care, Waterloo Ontario, Canada; [g]Université Laval, Faculty of Nursing, Quebec City, Quebec, Canada; [h]Université Laval, School of Psychology, Quebec City, Quebec, Canada; [i]Université Laval, Faculty of Medicine, Quebec City, Quebec, Canada; [j]Université Laval, Department of Psychiatry and Neurosciences, Quebec City, Quebec, Canada; [k]University of Rochester Medical Center, School of Medicine and Dentistry Rochester, New York; [l]Université Laval, Faculty of Medicine, Quebec City, Quebec, Canada

**Introduction/Aim**: Chemotherapy-induced peripheral neuropathy (CIPN) is a common, distressing, and disabling effect of taxane-based therapy. Few treatments have been identified, in part due to a lack of sufficiently validated patient-reported outcomes (PRO). We evaluated the content validity of 3 commonly recommended CIPN PROs.

**Methods**: Women completing taxane-based chemotherapy reporting ≥2 CIPN symptoms were asked to “think-aloud” while responding to 3 CIPN PROs: Functional Assessment of Cancer Therapy-Taxane subscale (FACT-Taxane); modified Treatment-Induced Neuropathy Assessment Scale (TNAS); Short-Form Chemotherapy-induced Peripheral Neuropathy Assessment Tool (CIPNAT-SF). Sociodemographic and clinical data were collected and qualitative inductive-deductive coding was performed to identify 3 item-related problem types: comprehensibility, comprehensiveness, and relevance.

**Results**: 51 interviews among 38 women were conducted (n=28, <10 days post final cycle [T1]; n=23, 3 months later [T2]). 13 women completed both interviews. Participants were 49±11 years old and had completed 17±3 years education. 68% received paclitaxel. Women described difficulty distinguishing tingling from numbness when presented as separate items (CIPNAT-SF), and responding to single items asking about multiple locations (e.g. hands/feet; TNAS). CIPNAT-SF was perceived as most comprehensive due to interference items; however, work and driving-related interference were often irrelevant. Among women who completed both interviews, general weakness (FACT-Taxane) was interpreted as fatigue at T1, but motor weakness at T2. Distress (CIPNAT-SF) was more relevant at T2 than T1.

**Discussion/Conclusions**: This study identifies comprehension, comprehensiveness, and relevance problems in commonly recommended CIPN PROs. Comprehension and relevance of certain items may differ for acute versus chronic CIPN. Refinement is necessary to improve content validity.

### ORCID

Sophie Lauzier http://orcid.org/0000-0002-7746-9208
Philip Jackson http://orcid.org/0000-0001-8991-3217
Lynn R Gauthier http://orcid.org/0000-0003-0138-0167

*Senior authors, equal contribution

© 2021 The Author(s). Published with license by Taylor & Francis Group, LLC.
This is an Open Access article distributed under the terms of the Creative Commons Attribution License (http://creativecommons.org/licenses/by/4.0/), which permits unrestricted use, distribution, and reproduction in any medium, provided the original work is properly cited.

### Proglumide reduces enhanced nociception in mice that interact with a social partner in pain

Sandra J. Poulson[a], Iqra Arain[a], Keza Motlana[a], and Loren J. Martin[b]

[a]University of Toronto Mississauga, Department of Psychology, Mississauga, Ontario, Canada; [b]University of Toronto Mississauga, Department of Psychology, Cell & Systems Biology, Mississauga, Ontario, Canada

**Introduction/Aim**: Social animals can sense environmental threats via watching group members. Research in mice has shown that when together, familiar mice express more pain than when alone, and observer mice develop sensitivity to painful stimuli after interacting with cagemates in pain. Yet the neural mechanisms that drive the enhancement of pain due to social stimuli are not known. Our aim was to measure whether social signals subsequently increase pain behavior in the observer.

**Methods**: We developed a paradigm consisting of three phases: habituation, social interaction, and post-test. In the 1-hour habituation phase, two familiar male C57BL/6 mice were placed in a cubicle with one deemed observer and the other deemed demonstrator. In the social interaction phase, the demonstrator was briefly removed, an intraplantar injection of formalin (2.5%, 20µl, n=8) or saline (20µl, n=8) was applied, and the demonstrator was replaced. Social interaction was recorded over 30 minutes. In the post-test phase, the observer was briefly removed, formalin (0.5%, 20μl) intraplantar injection applied, observer returned to cubicle, and hindpaw licking/chewing behavior recorded 1hr. In one group (n=8), observer mice were pretreated with proglumide (10mg/kg), an antagonist of cholecystokinin (CCK) receptors.

**Results**: We found increased licking/chewing in the posttest after observer mice were paired with a pain demonstrator compared to saline controls. This enhanced licking/chewing due to social signals was reduced by pretreatment with proglumide.

**Discussion/Conclusions**: Our results indicate that interacting with cagemates in pain enhanced the perception of painful stimuli. Additionally, reduction of licking/chewing by proglumide suggests that CCK plays a role in the enhancement of pain due to social signals.

### ORCID

Sandra J. Poulson http://orcid.org/0000-0002-5533-4599
Loren J. Martin http://orcid.org/0000-0003-0579-2441

© 2021 The Author(s). Published with license by Taylor & Francis Group, LLC.
This is an Open Access article distributed under the terms of the Creative Commons Attribution-NonCommercial License (http://creativecommons.org/licenses/by-nc/4.0/), which permits unrestricted non-commercial use, distribution, and reproduction in any medium, provided the original work is properly cited.

### Deriving and Validating a Brain-Based Biomarker for Chronic Pain

Matt Fillingim[a], Christophe Tanguay-Saborin[a], and Etienne Vachon-Presseau[b]

[a]Mcgill University, Neuroscience, Montreal, Quebec, Canada; [b]Mcgill University, Dentistry, Montreal, Quebec, Canada

**Introduction/Aim**: There is a critical need for the development of better diagnostic and prognostic tools to accurately identify and predict the genesis, development, and trajectory of chronic pain. This study applied multivariate machine on neuroimaging data from the UKBiobank (UKBB, n=2,335) data set to train and validate a biomarker that distinguishes people without pain from people reporting multiple sites of chronic pain.

**Methods**: A 'high impact' chronic pain group was engineered from a UKBB sample (n=20,000) by selecting individuals with multi-site pain (num. pain sites > 1) and then conditionally filtering their scores on four psychosocial variables that are positively correlated with chronic pain (overall health, fatigue, neuroticism, sleep). The UKBB dataset was divided into a train set (70%) and a validation set (30%). Machine learning pipelines were applied to neuroanatomical (~771) and neurophysiological (38,781) brain features of individuals with multi-site chronic pain (n=1121) and healthy controls (n=1214).

**Results**: A gradient boosting decision tree classifier determined the highest test set accuracy of 60%, with a micro average area under the curve score of 0.66.

**Discussion/Conclusions**: The classifier reports well-above chance ability to discriminate healthy controls from individuals with multiple sites of chronic pain (ROC=.66). Given the large sample size the classifier was trained on, it should generalize well to unseen data. The derived classifier will next be tested in an independent dataset, OpenPain, to assess the generalizability of the biomarker. This biomarker may serve as an effective tool for the diagnosis of chronic pain.

© 2021 The Author(s). Published with license by Taylor & Francis Group, LLC.
This is an Open Access article distributed under the terms of the Creative Commons Attribution License (http://creativecommons.org/licenses/by/4.0/), which permits unrestricted use, distribution, and reproduction in any medium, provided the original work is properly cited.

### Using Visual Arts-Based Methods to Explore Chronic Pain: A Photovoice Exploration into Chronic Pain Experiences of Immigrant Indian Women in Canada

Nida Mustafa[a], Margaret MacNeill[b], Judy Watt-Watson[c], and Gillian Einstein[d,e]

[a]University of Toronto, Dalla Lana School of Public Health, Toronto, Ontario, Canada; [b]University of Toronto, Graduate Department of Exercise Sciences, Toronto, Ontario, Canada; [c]University of Toronto, Lawrence S. Bloomberg Faculty of Nursing, Toronto, Ontario, Canada; [d]University of Toronto, Department of Psychology, Toronto, Ontario, Canada; [e]Linköping University, Department of Gender Studies, Linköping, Sweden

**Introduction/Aim**: Although quantitative measures are useful in identifying pain prevalence and severity, qualitative methods, and especially visual arts-based ones, are now receiving attention as a valuable means to understand lived experiences of pain. Photovoice – a method which utilizes photography to document individuals' lives – aims to enhance the understanding of experiences by creating opportunities for story-sharing using the individual's own voice.

**Methods**: The current study utilized photovoice methods to explore twelve immigrant Indian women's chronic pain experiences in Canada.

**Results**: Women's photographs, and description of these photographs, provided a visual entry into their lives and the socio-cultural dimensions of their pain experiences. Findings revealed women's representations of pain were shaped by a clash between culturally shaped gender expectations and changing roles due to immigration processes.

**Discussion/Conclusions**: The use of photovoice not only visually contextualized and represented pain experiences, but also proved to be a valuable tool for self-reflection and change in the lives of the women in this study.

### ORCID

Gillian Einstein http://orcid.org/0000-0002-0770-5471

© 2021 The Author(s). Published with license by Taylor & Francis Group, LLC.
This is an Open Access article distributed under the terms of the Creative Commons Attribution License (http://creativecommons.org/licenses/by/4.0/), which permits unrestricted use, distribution, and reproduction in any medium, provided the original work is properly cited.

### Qualitative Analysis of Chronic Pain Program Management Outcomes: Veterans and Civilians

Christina Hanna[a], Eleni Hapidou[b], Victoria Borg Debono[c], Jennifer Anthonypillai[d], Lisa Patterson[e], Sonya Altena[f], Eric Pham[g], and Ramesh Zacharias[h]

[a]McMaster University, Hamilton, Ontario, Canada; [b]Hamilton Health Sciences, Hamilton, Ontario, Canada; [c]McMaster University, Hamilton, Ontario, Canada; [d]Hamilton Health Sciences, McMaster University, Hamilton, Ontario, Canada; [e]Niagara Health, B.A., St. Catharines, Ontario, Canada; [f]Pediatric Complex Care, McMaster Children's Hospital, Hamilton Health Sciences, Hamilton, Ontario, Canada; [g]Department of Psychology, Neuroscience and Behaviour, McMaster University, Hamilton, Ontario, Canada; [h]Hamilton Health Sciences, McMaster University, Hamilton, Ontario, Canada

**Introduction/Aim**: The objective of this study was to evaluate the long-term effectiveness of a 4-week adult interdisciplinary chronic pain management program through qualitative analysis, while also conducting a sub-group analysis identifying differences between veterans and civilians.

**Methods**: This study used a mixed-methods approach. The quantitative data for this study are descriptive statistics for patient demographics and satisfaction measures using SPSS Statistics software. The qualitative data were collected through patient comments on two satisfaction measures, Pain Program Satisfaction Questionnaire (PPSQ) and the Self-Evaluation Scale (SES). Of 205 participants who completed the program, 67 returned FU measures. Comments were independently organized into themes using an inductive approach by four researchers

**Results**: Ten main themes were deduced: (1) Gratefulness for treatment and program, (2) improvement in ability, (3) new sense of resiliency, ability to cope, new confidence, (4) empowered over recovery, (5) on the path to rehabilitation and recovery - a process and journey, (6) the need for support groups, (7) while some patients are doing well, there are some that still struggle, (8) patients need continued follow-up to ensure they are on track and they don't regress – Continued follow-up needed, (9) timeliness of program - needed earlier in course of chronic pain, (10) program merits recommendation. Veteran comments were coded on (1), (2), (3), (5), (7), (8) and (10)

**Discussion/Conclusions**: Results emphasize the effectiveness of interdisciplinary care for managing chronic pain in both veterans and civilians The qualitative analysis provides opportunities for clinicians to improve quality and apply new services to the program.

© 2021 The Author(s). Published with license by Taylor & Francis Group, LLC.
This is an Open Access article distributed under the terms of the Creative Commons Attribution License (http://creativecommons.org/licenses/by/4.0/), which permits unrestricted use, distribution, and reproduction in any medium, provided the original work is properly cited.

### Alterations in brain functional connectivity in chronic pain patients using opioids

Azin Zare[a], Christophe Tanguay Sabourin[b], Gianluca Guglietti[a], Matthew Fillingim[b], Yueh En Wang[a], Marc O. Martel[a], Mathieu Roy[c], Luda Diatchenko[a], and Etienne Vachon-Presseau[a]

[a]McGill University, Dentistry, Montreal, Canada; [b]McGill University, Integrated Program in Neuroscience, Montreal, Canada; [c]McGill University, Department of Psychology, Montreal, Canada

**Introduction/Aim**: Opioids remain a leading treatment for chronic pain despite the serious risks associated with opioid use. Research has shown that chronic pain and opioid use are both related to abnormal brain functional connectivity, especially between the corticolimbic regions. Yet, these studies have been limited in generalizability by their use of small numbers. In this study, we investigated the extent to which global functional connectivity within the limbic brain regions are associated with opioid use and chronic pain in a large population cohort.

**Methods**: An observational study was conducted using data of 20,000 participants from the UK-Biobank. Data included self-reported regularly used medications, pain status and resting-state functional (rsFC) brain imaging. Global functional connectivity was calculated by averaging the correlations of BOLD signal between regions of interest within a defined corticolimbic circuitry. After controlling for potential covariates (i.e., sex and age), we compared functional connectivity across chronic pain patients using opioids (n=271), chronic pain patients not using opioids (n=6871) and healthy controls (n =12594) using one-way ANCOVAs.

**Commented:** Across identified regions, global connectivity strength for the hypothalamus and the cingulate cortex showed a small but significant positive correlation among groups (p=1.14e-5, f=19.2, p=6e-4, f=11.5, respectively). After Bonferroni correction, the correlation of these regions also survived the corrected threshold of p<7e-5.

**Discussion/Conclusions**: These findings suggest a pattern of altered rsFC in the hypothalamus with the rest of the corticolimbic circuitry in chronic pain patients reporting using opioids. These results build on previous findings suggesting that opioids may alter the brain circuitry regulating the neuroendocrine system.

### ORCID

Mathieu Roy http://orcid.org/0000-0002-3335-445X
Luda Diatchenko http://orcid.org/0000-0002-1350-6727

© 2021 The Author(s). Published with license by Taylor & Francis Group, LLC.
This is an Open Access article distributed under the terms of the Creative Commons Attribution License (http://creativecommons.org/licenses/by/4.0/), which permits unrestricted use, distribution, and reproduction in any medium, provided the original work is properly cited.

### Hyperalgesia corresponds with changes in amyloid-beta concentration in the spinal dorsal horn.

Laura A. Bennett and Robert P. Bonin

University of Toronto, Pharmaceutical Sciences, Toronto, Ontario, Canada

**Introduction/Aim**: The synaptic plasticity that allows for memory in the brain is mechanistically and functionally analogous to the synaptic plasticity that occurs at neurons in the dorsal horn of the spinal cord. The small peptide, amyloid beta (Aβ), is associated with memory loss in Alzheimer's disease and modulates synaptic plasticity towards synaptic depression. The exact mechanism by which Aβ acts on synapses is poorly understood and this is largely because Aβ is present at endogenously low concentrations in brains of healthy individuals. It is not currently known whether Aβ contributes to synaptic plasticity and sensory processing in the spinal dorsal horn.

**Methods**: We first investigated the relationship between hyperalgesia and spinal Aβ levels. Intraplantar injections of capsaicin induce hyperalgesia which is measured by paw withdrawal threshold using Von Frey filaments. Dorsal spinal cords were extracted, and Enzyme Immunosorbent Assay was used to quantify $A\beta_{1\text{-}42}$– the Aβ oligomer associated with synaptic depression.

**Results**: Since central processes of hyperalgesia and Aβ production are likely dependent on neuronal activity, we predicted that a decrease in paw withdrawal threshold in response to capsaicin injection would be paralleled by an increase Aβ levels. In preliminary results we observed an increase in dorsal spinal cord $A\beta_{1\text{-}42}$ concentration as paw withdrawal threshold decreased in male mice but not female mice.

**Discussion/Conclusions**: Future experiments will involve pharmacologically and genetically modifying

Aβ levels and evaluating hyperalgesia via changes in paw withdrawal thresholds. Our preliminary results suggest Aβ may play a role in synaptic plasticity of the dorsal spinal cord, contributing to hyperalgesia.

© 2021 The Author(s). Published with license by Taylor & Francis Group, LLC.
This is an Open Access article distributed under the terms of the Creative Commons Attribution License (http://creativecommons.org/licenses/by/4.0/), which permits unrestricted use, distribution, and reproduction in any medium, provided the original work is properly cited.

### Pain-related and situational catastrophizing indices of sensitivity to physical activity are associated with daily life pain intensity for back pain

Arthur Woznowski-Vu[a], Marc O. Martel[b], Alexandre Gervais[a], Sara Ahmed[a], Michael Sullivan[c], and Timothy Wideman[a]

[a]McGill University, School of Physical and Occupational Therapy, Montreal, Quebec, Canada; [b]Faculties of Dentistry & Medicine, Montreal, Quebec, Canada; [c]McGill University, Department of Psychology, Montreal, Quebec, Canada

**Introduction/Aim**: Certain individuals with back pain experience negative responses to acute bouts of physical activity. Previous research has studied this phenomenon using indices of sensitivity to physical activity (SPA) evoked by a standardized physical task. SPA indices have been shown to have prognostic value for pain-related outcomes, but their ecological validity in relation to the daily life pain experience remains unknown. This study aims to address this gap.

**Methods**: Adults with back pain (<six-month onset) completed questionnaires and a SPA task. This task consisted of ten repeated lifts, whereby the first lift was individually tailored to evoke clinically important pain (≥20 on 0-100 scale). In relation to this task, SPA indices were recorded: SPA-Pain (changes in evoked pain intensity), SPA-Sensory (changes in pressure pain threshold at the back and hands), and SPA-Psych (Situational Catastrophizing Questionnaire). Afterwards, smartphone-based ecological momentary assessment (EMA) collected daily life momentary ratings of pain intensity, mood, and physical activity, using randomized prompts (8am-12pm;12-4pm;4-8pm) for nine days. Multilevel linear model (random intercept) analyses were carried out per independent variable of interest (SPA indices, centered) while controlling for significant covariates.

**Results**: Based on 62 participants (EMA median completion rate 59%), analyses revealed that SPA-Pain (estimate of fixed effect=0.467,p-value<0.001) and SPA-Psych (estimate of fixed effect=1.350,p=0.021) were associated with daily life pain intensity, even after controlling for scores in Pain Catastrophizing Scale and daily life physical activity and mood.

**Discussion/Conclusions**: This study provides initial ecological validation support for SPA, showing it is uniquely associated with EMA-based daily life pain intensity.

### ORCID

Sara Ahmed http://orcid.org/0000-0001-5172-6790
Michael Sullivan http://orcid.org/0000-0002-4228-1678

© 2021 The Author(s). Published with license by Taylor & Francis Group, LLC.
This is an Open Access article distributed under the terms of the Creative Commons Attribution License (http://creativecommons.org/licenses/by/4.0/), which permits unrestricted use, distribution, and reproduction in any medium, provided the original work is properly cited.

### Understanding the consequences of the COVID-19 pandemic in the daily living of people with chronic pain: A pan-Canadian qualitative study

Lise Dassieu PhD[a], M. Gabrielle Pagé PhD[a,b,c], Anaïs Lacasse PhD[d], Maude Laflamme MSc[a,e], Vickie Perron MSc[f], Audrée Janelle-Montcalm MSc[a], Maria Hudspith MA[f], Gregg Moor BA[f], Kathryn Sutton[f], James M Thompson MD[g], and Manon Choinière PhD[a,b]

[a]Research Center of the Centre Hospitalier de l'Université de Montréal (CRCHUM), Saint-Antoine Building, 850 Saint Denis St., Montreal, Quebec, H2X 0A9, Canada; [b]Université de Montréal, Faculty of Medicine, Department of Anesthesiology and Pain Medicine, Pavillon Roger-Gaudry, C.P. 6128, succursale Centre-ville, Montreal, Québec, H3C 3J7, Canada; [c]Université de Montréal, Faculty of Arts and Science, Department of Psychology, Pavillon Marie-Victorin, 90 avenue Vincent d'Indy, Montreal, Québec, H2V 2S9, Canada; [d]Université du Québec en Abitibi-Témiscamingue (UQAT), Department of Health Sciences, Rouyn-Noranda, Quebec, Canada; [e]Université de Montréal, Faculty of Arts and Sciences, Department of Sociology, Montreal, Québec, H3C 3J7, Canada; [f]Pain BC Society, Vancouver, British Columbia, Canada; [g]Department of Public Health Sciences, Queens University and Department of Family Medicine, Dalhousie University

**Introduction/Aim**: The COVID-19 pandemic has had disruptive effects on daily living and caring routines for people with chronic pain (CP). This qualitative study aimed to understand the impacts of the pandemic on CP management, psychological well-being and social situation for people with CP.

**Methods**: In-depth semi-structured interviews were conducted with 22 individuals living with CP (>3 months) from six Canadian provinces (May-August 2020). To capture diverse perspectives, participants from ethnic and/or gender minorities represented approximately half of the sample.

**Results**: Firstly, the study findings underscored the complexity of accessing pain management during the pandemic, as well as the reluctance to use health services for participants feeling vulnerable to the virus because of their CP condition. Several participants reported pain flares due to increased anxiety and reduced physical activity. Furthermore, the pandemic intensified their psychological distress, with many reporting suicidal ideation during the lockdown period. Social isolation, poorly managed pain, financial difficulties and concomitant mental health problems seemed to increase their psychological vulnerability. Finally, several participants reported positive outcomes of the pandemic, such as a "slower" way of life more adapted to their CP condition. However, participants from deprived social groups were less likely to benefit from positive aspects.

**Discussion/Conclusions**: This study underscores the intermingling of physical, psychological and social impacts of the pandemic for people with CP. In the perspective of future COVID-19 waves and public health crises, psycho-social interventions supporting people with CP facing both social inequities and psychological difficulties are highly needed.

### ORCID

Lise Dassieu PhD http://orcid.org/0000-0002-6311-3681
M. Gabrielle Pagé PhD http://orcid.org/0000-0002-7742-2717
Anaïs Lacasse PhD http://orcid.org/0000-0002-3992-5145
James M Thompson MD http://orcid.org/0000-0001-7443-8732
Manon Choinière PhD http://orcid.org/0000-0001-9593-8883

© 2021 The Author(s). Published with license by Taylor & Francis Group, LLC.
This is an Open Access article distributed under the terms of the Creative Commons Attribution License (http://creativecommons.org/licenses/by/4.0/), which permits unrestricted use, distribution, and reproduction in any medium, provided the original work is properly cited.

### Smartphone apps targeting menstrual pain and symptoms: A scoping review

Lindsey C. M. Trépanier[a], Nicole M. Alberts[b], and Michelle M. Gagnon[c]

[a]University of Saskatchewan, Department of Psychology and Health Studies, Saskatoon, SK, Canada; [b]Concordia University, Department of Psychology, Montreal, QC, Canada; [c]University of Saskatchewan, Department of Psychology and Health Studies, Saskatoon, SK, Canada

**Introduction/Aim**: Menstrual pain occurs in up to 90% of individuals during menstruation and can impact functioning. Smartphones apps are often used by individuals who menstruate to track facets of their menstrual experience and present a viable self-management tool for individuals who experience menstrual pain. Inclusion of self-management content (e.g., mindfulness, relaxation) in apps has not been previously examined. We examine the content and quality of self-management apps targeting menstrual pain and symptoms.

**Methods**: A scoping review of apps targeting facets of the menstrual experience was conducted by searching the App Store using search terms related to menstruation, menstrual tracking, and menstrual pain. App engagement, functionality, design aesthetics, and information quality were evaluated using the Mobile App Rating Scale. Frequency and quality of pain tracking and pain self-management content quality was evaluated.

**Results**: After removal of duplicates and screening, 162 apps were retained. Apps varied greatly in terms of engagement and functionality. A stereotypically "feminine" esthetic (e.g., pink, flowers) was central to the design of most apps. Many apps incorporated incorrect information and spelling/grammar errors. Apps rarely described inclusion of health providers in the development process. Pain and associated symptoms tracking was an option in 112 apps. Six apps included self-management options such as meditation, yoga, pain-management, and wellness coaching.

**Discussion/Conclusions**: Although pain and symptom tracking functions are common, most apps do not incorporate a self-management function. Improving self-management content in menstrual apps may present a potential future direction to improve management of menstrual pain.

© 2021 The Author(s). Published with license by Taylor & Francis Group, LLC.
This is an Open Access article distributed under the terms of the Creative Commons Attribution-NonCommercial License (http://creativecommons.org/licenses/by-nc/4.0/), which permits unrestricted non-commercial use, distribution, and reproduction in any medium, provided the original work is properly cited.

### Chronic pain, stress and gender in the context of the COVID-19 pandemic

Catherine Côté[a], Élise Develay[b], Mathieu Roy[c], Étienne Vachon-Presseau[d], Sonia Lupien[e], Pierre Rainville[e], and M Gabrielle Pagé[e]

[a]Université de Montréal, Psychologie, Montréal, Québec, Canada; [b]CRCHUM, Montréal, Québec, Canada; [c]McGill University, Psychologie, Montréal, Québec, Canada; [d]McGill University, Dentistry, Montréal, Québec, Canada; [e] Université de Montréal, Psychologie, Montréal, Québec, Canada

**Introduction/Aim**: Chronic pain affects up to 25% of Canadians and its prevalence is higher among women. Living with chronic pain can impact mental health. The COVID-19 pandemic could also put individuals living with chronic pain at risk of deterioration of their pain and psychological status, especially among women. This study explores stress characteristics and gender differences in pain in the context of the COVID-19 pandemic.

**Methods**: Forty-nine participants completed online questionnaires before the pandemic as well as during and after the 1st wave. Self-reported measures of pain characteristics, general and pandemic-specific stress levels, and psychological distress were administered electronically to individuals living with chronic pain. Descriptive statistics, chi-square tests and logistic regressions were used to analyze data.

**Results**: Two thirds of participants reported a deterioration in their pain status since the beginning of the pandemic. Perceiving the pandemic as stressful due to its novelty, uncontrollability, unpredictability and threat to the ego was associated. The proportion of individuals with moderate to severe psychological distress remained relatively stable from pre-pandemic (37%) to during the first wave of the pandemic (42%); however this proportion decreased to 32% after the first wave. There were no gender difference in pain status, perceived stress, loneliness, or psychological distress during the pandemic.

**Discussion/Conclusions**: These results suggest the importance of stress characteristics as they can influence pain deterioration. More research is needed regarding the association between pain and gender, especially in the context of the COVID-19 pandemic.

### ORCID

Sonia Lupien http://orcid.org/0000-0002-8763-393X
M Gabrielle Pagé http://orcid.org/0000-0002-7742-2717

© 2021 The Author(s). Published with license by Taylor & Francis Group, LLC.
This is an Open Access article distributed under the terms of the Creative Commons Attribution License (http://creativecommons.org/licenses/by/4.0/), which permits unrestricted use, distribution, and reproduction in any medium, provided the original work is properly cited.

### Perioperative Rehabilitation for Chronic Pain Patients Using Online Approach at a Community Based Clinic.

Brenda Lau, Emmanuel Abreu, and Himanshu Khetarpal

CHANGEpain Clinic, Vancouver, British Columbia, Canada

**Introduction/Aim**: CHANGEpain in collaboration with Doctors Of BC, implemented a virtual peri-operative optimization program to support existing BC efforts in improving outcomes after surgery. This program offers expedited access to consultations with a pain specialist in which 7 components are critically assessed. The current study aimed to assess the feasibility and sustainability of online services for peri-optimization. It is also aimed at gathering preliminary understanding of the post-surgical complications faced by chronic pain patients who underwent optimization.

**Methods**: Chronic pain patients were referred to the clinic by their primary care provider or surgeon. 176 Patients were triaged during September to November 2020. These patients were triaged and prepared by a research assistant and consultations were completed with a pain specialist. Data collection methods included standardized questionnaires that were analyzed using SPSS.

**Results**: 176 Patients were triaged during September to November 2020. Most patients (74%) required some sort of optimization after initial consult. Approximately half (49%) of patients were within the 61-80 years old age group. The post-surgical outcomes, included, unplanned operation and hospital readmission (4%), cardiac arrest requiring, CPR and acute renal failure (2%).

**Discussion/Conclusions**: Virtual optimization significantly improved access to peri-surgical optimization services by overcoming geographical barriers. However, remote care presented multiple new administrative challenges such as inadequate information transfer that resulted in delay of patients' access to the program. To date, there are no other baseline statistics on post-surgical complications faced by BC chronic pain patients let alone how these changes with optimization. Subsequent studies are needed to understand the robustness of the statistical baselines captured.

### ORCID

Brenda Lau http://orcid.org/0000-0002-0090-016X

Emmanuel Abreu http://orcid.org/0000-0002-0295-214X

© 2021 The Author(s). Published with license by Taylor & Francis Group, LLC.
This is an Open Access article distributed under the terms of the Creative Commons Attribution License (http://creativecommons.org/licenses/by/4.0/), which permits unrestricted use, distribution, and reproduction in any medium, provided the original work is properly cited.

### The use of Peripheral Nerve Imaging and Quantitative Sensory Testing to Classify Pediatric Participants with Neuropathic Pain

Anastasia Karapanagou[a], Christine Sieberg[a], Laura Simons[b], Alyssa Lebel[a], David Zurakowski[a], David Borsook[a], and Scott Holmes[a]

[a]Boston Children's Hospital, Department of Anesthesiology, Critical Care and Pain Medicine, Boston, Massachusetts, United States; [b]Stanford University School of Medicine, Department of Anesthesiology, Perioperative and Pain Medicine, Palo Alto, California, United States

**Introduction:** Persons with neuropathic pain resulting from peripheral nerve damage may experience changes in thermal sensitivity, pain symptom reporting, and altered nerve fiber characteristics. The aim of this investigation was to evaluate changes in pain thresholds of thermal sensitivity and their relationship with nerve fiber characteristics and pain symptoms to classify pediatric subjects with neuropathic pain.

**Methods:** We recruited 41 patients with clinically confirmed neuropathic pain (age range:10-25) within 3 months of an ankle injury and 17 age and sex-matched healthy controls. Participants underwent diffusion tensor imaging (DTI) of the sciatic, tibial and fibular nerves. Participants completed a psychological battery and Quantitative Sensory Testing (QST). Independent Samples T-Test was used to investigate group differences. All metrics were normalized and submitted to Principal Component Analysis (PCA). Logistic regression was used to classify persons with neuropathic pain from healthy controls.

**Results:** Patients had greater visual analogue scale (VAS) scores than healthy controls for hot ($p<0.03$) and cold stimuli ($p<0.021$) and scored higher on measures of fear of pain ($p=0.007$), pain catastrophizing ($p=0.047$) and pain screening ($p<0.001$). Significant correlations were found between the VAS and QST Heat Scores, and the PCA analysis found 7 components of interest. Logistic regression showed that component 5 (Nerve Fiber: FA(sciatic), MD(tibial) and RD(tibial), QST: Heat pain) was found to be the only significant component with a value of .009.

**Discussion:** Findings underscore the complementary role for QST and peripheral nerve imaging to be used in evaluating persons with neuropathic pain.

### ORCID

Laura Simons http://orcid.org/0000-0002-3395-9483

© 2021 The Author(s). Published with license by Taylor & Francis Group, LLC.
This is an Open Access article distributed under the terms of the Creative Commons Attribution License (http://creativecommons.org/licenses/by/4.0/), which permits unrestricted use, distribution, and reproduction in any medium, provided the original work is properly cited.

### Kinesiophobia and its relation to activity limitation after multidisciplinary rehabilitation in patients with chronic pain

Myranda Rocha Martinez[a], Eleni G. Hapidou[b], Adria Fransson[c], and Eric Pham[a]

[a]Department of Psychology, Neuroscience and Behavior (PNB), McMaster University; [b]Hamilton Health Sciences and McMaster University, Michael G. DeGroote Pain Clinic, Hamilton, ON, Canada; [c]Hamilton Health Sciences, Michael G. DeGroote Pain Clinic, Hamilton, ON, Canada

**Introduction/Aim**: The purpose of this study was to examine changes in kinesiophobia in relation to activity limitation, pain catastrophizing and pain self-efficacy in an interdisciplinary rehabilitation program in patients with heterogeneous chronic pain. This is a partial replication of a study by Lüning Bergsten, Lundberg, Lindberg and Elfving (2012).

**Methods**: Data from baseline ($T_0$) and four weeks after rehabilitation ($T_1$) were analyzed for 189 patients who completed the intensive program at the Michael G. DeGroote Pain Clinic from July 2015 to January 2020. Outcome measures were the Tampa Scale for Kinesiophobia (TSK-11, the Patient Disability Index (PDI), the Pain Catastrophizing Scale (PCS), and the Pain Self-Efficacy Questionnaire (PSEQ).

**Results**: The effect of three levels of kinesiophobia (low, medium and high baseline TSK scores) was examined on the above measures, and for those patients who did or did not reach the smallest detectable change (SDC) on the TSK.

Kinesiophobia, pain catastrophizing and pain disability levels decreased from $T_0$ to $T_1$ ($p<.001$) whereas pain self-efficacy increased ($p<.001$). There were significant differences across the three levels of the TSK scores at both $T_{0 \text{ and}}$ $T_1$ in all variables. Limited effects of gender were obtained. About half (44.95%) of the participants reached the SDC with most having high $T_0$ TSK scores.

**Discussion/Conclusions**: Results demonstrate that interdisciplinary rehabilitation programs effectively reduce kinesiophobia, activity limitation, and catastrophizing, and increase self-efficacy in individuals with chronic pain. These results can help therapists who provide rehabilitation and pain management therapies. Partial replication of the study by Lüning Bergsten et al. (2012) was obtained.

© 2021 The Author(s). Published with license by Taylor & Francis Group, LLC.
This is an Open Access article distributed under the terms of the Creative Commons Attribution-NonCommercial License (http://creativecommons.org/licenses/by-nc/4.0/), which permits unrestricted non-commercial use, distribution, and reproduction in any medium, provided the original work is properly cited.

### Chronic Pain Program Management Outcomes: Long Term Follow-up for Veteran and Civilian Men and Women

Eleni Hapidou Ph.D., C. Psych.[a], Eric Pham[b], Kate Bartley MSc.[c], Jennifer Anthonypillai BSc.[d], Sonya Altena[e], Lisa Patterson B.A.[f], and Ramesh Zacharias M.D.[g]

[a]Hamilton Health Sciences and McMaster University, Michael G. DeGroote Pain Clinic, Hamilton, ON, Canada; [b]Department of Psychology, Neuroscience and Behavior (PNB), McMaster University; [c]Hamilton Health Sciences and McMaster University, Michael G. DeGroote Pain Clinic, Hamilton, ON, Canada; [d]Chronic Pain, Hamilton, ON, Canada; [e]System Navigator, Pediatric Complex Care, McMaster Children's Hospital; [f]Niagara Health System, Niagara Falls; [g]Hamilton Health Sciences and McMaster University, Michael G. DeGroote Pain Clinic, Hamilton, ON, Canada,

**Introduction/Aim**: It has been established in the literature that interdisciplinary pain management programs are the most effective treatments for chronic pain. Often, patients see improvements in many pain-related variables, such as pain intensity and disability upon discharge from the program. Previous studies have overlooked exploring different subgroups in terms of their response to treatment. The present study explored how veterans and civilians and males and females, differ in their treatment outcomes from the 4-week interdisciplinary pain management program of the Michael G. DeGroote Pain Clinic.

**Methods**: Data were obtained from psychometric measures administered at admission and discharge from the program, and mailed to all completers at follow up (FU), four months to four years after discharge from the Program.

**Results**: A total of 67 participants (31 veterans and 36 civilians) out of the 204 contacted completed all psychometric measures at FU. Analysis of variance (ANOVA), 3 x 2 x 2 repeated measures were conducted to examine session (admission, discharge, FU), veteran status (veteran, civilian), and gender (male, female). Post-hoc tests were completed with Bonferroni correction. Results showed that the program was effective for all patients. For most measures, patients improved significantly at discharge and maintained their gains at FU. Females reported overall higher depression scores, and higher pain-related disability at follow up.

**Discussion/Conclusions**: The findings of the present study add support for the long-term efficacy of interdisciplinary pain management programs for all patients.

© 2021 The Author(s). Published with license by Taylor & Francis Group, LLC.
This is an Open Access article distributed under the terms of the Creative Commons Attribution License (http://creativecommons.org/licenses/by/4.0/), which permits unrestricted use, distribution, and reproduction in any medium, provided the original work is properly cited.

### Long-term Safety and Tolerability of Atogepant Following Once Daily Dosing Over 1 Year for the Preventive Treatment of Migraine

Messoud Ashina[a], Stewart Tepper[b], Uwe Reuter[c], Andrew Blumenfeld[d], Susan Hutchinson[e], Jing Xia[f], Rosa Miceli[g], Lawrence Severt[g], Michelle Finnegan[g], and Joel Trugman[g]

[a]Danish Headache Center, Neurology, Rigshospitalet Glostrup, University of Copenhagen, Denmark; [b]Dartmouth Geisel School of Medicine, Neurology, Hanover, NH, USA; [c]Charité University Hospital, Neurology, Berlin, Germany; [d]Headache Center of Southern California, Carlsbad, CA, USA; [e]Orange County Migraine and Headache Center, Irvine, CA, USA; [f]AbbVie, Biostatistics, Madison, NJ, USA; [g]AbbVie, Clinical Development, Madison, NJ, USA

**Introduction/Aim**: Atogepant is an oral, small molecule, CGRP receptor antagonist in development for the preventive treatment of migraine. We evaluated the safety and tolerability of atogepant over 52 weeks.

**Methods**: Multicenter, open-label trial (NCT03700320). Adults with migraine were randomized 5:2 to atogepant 60 mg once-daily or oral standard-of-care migraine prevention medicine. The primary objective was to assess the safety and tolerability of atogepant. Standard-of-care was included to primarily help contextualize hepatic safety data.

**Results**: The trial included 744 randomized participants (n=546 atogepant) with 739 in the safety population (n=543 atogepant). Adverse events (AEs) were reported by 67.0% of atogepant-treated participants; 18.0% reported AEs that were considered treatment-related. Most commonly reported AEs (≥5% of participants) were upper respiratory tract infection (10.3%), constipation (7.2%), nausea (6.3%), and urinary tract infection (5.2%) following atogepant treatment. Serious AEs were reported by 4.4% of atogepant-treated participants; no event was seen in >1 participant and no event was considered treatment-related. Two deaths were reported in atogepant-treated participants (homicide victim and a group A beta-hemolytic streptococcal sepsis [toxic shock syndrome]); both were considered not related to atogepant. Discontinuation due to AEs was 5.7% following atogepant treatment. Cases of alanine aminotransferase/aspartate aminotransferase levels ≥3 times the upper limit of normal were reported for 2.4% of atogepant-treated participants (n=13/531) and 3.2% for standard-of-care (n=6/190). No cases of potential Hy's Law were reported.

**Discussion/Conclusions**: Long-term, once daily use of atogepant 60 mg for the preventive treatment of migraine over 1 year was safe and well-tolerated with no safety concerns identified.

### Ubrogepant is Effective in the Acute Treatment of Migraine with Mild Pain

Richard B. Lipton[a], David W. Dodick[b], Peter J. Goadsby[c], Rami Burstein[d], Aubrey M. Adams[e], Jeff Lai[f], Sung Yun Yu[g], Michelle Finnegan[g], and Joel M. Trugman[g]

[a]Albert Einstein College of Medicine and Montefiore Headache Center, Neurology, Bronx, NY, USA; [b] Mayo Clinic, Phoenix, AZ, USA; [c] NIHR-Wellcome Trust King's Clinical Research Facility, Neurology, London, England, UK; [d] Harvard Medical School, Beth Israel Deaconess Medical Center, Boston, MA, USA; [e] Medical Affairs, Irvine, CA, USA; [f] Biostatistics, Madison, NJ, USA; [g]Clinical Development, Madison, NJ, USA

**Introduction/Aim**: Ubrogepant, approved for the acute treatment of migraine, demonstrated efficacy in treating migraine with moderate/severe pain. Clinical guidance recommends treatment when pain is mild, a strategy studied here for ubrogepant.

**Methods**: Phase-3, open-label, 52-week trial (NCT02873221). Adults randomized 1:1:1 to usual care, or in blinded fashion to ubrogepant-50mg or ubrogepant-100mg, treated up to 8 migraine attacks every 4 weeks. Efficacy measures, collected only for ubrogepant, included 2-hour pain freedom and absence of migraine-associated symptoms.

**Results**: Participants' (n=808) mean age was 42 years; most were white and female. During the trial, 21,454 attacks were treated. Pain free rates were higher for the treatment of mild versus moderate/severe pain (average percentage across all treated attacks): 39% vs 19% ubrogepant-50mg (P<0.0001) and 43% vs. 21% ubrogepant-100mg (P<0.0001). Absence of photophobia was achieved in a higher proportion of attacks treated with mild pain versus moderate/severe pain: 55% both doses vs 34% both doses (P<0.0001). Absence of phonophobia was achieved in a higher proportion of attacks treated with mild pain versus moderate/severe pain: 64% vs 42% ubrogepant-50mg (P<0.0001) and 70% vs 45% ubrogepant-100mg (P<0.0001). Absence of nausea was achieved in a higher proportion of attacks treated with mild pain versus moderate/severe pain: 83% vs 67%

ubrogepant-50mg (P<0.0001) and 82% vs 68% ubrogepant-100mg (P<0.0001). Overall, 10% of participants reported treatment-related adverse events; one was considered serious (exacerbation of sinus tachycardia).

**Discussion/Conclusions**: Treating migraine patients with ubrogepant when headaches are mild rather than moderate/severe increases the likelihood of rendering them free of pain and associated symptoms.

© 2021 The Author(s). Published with license by Taylor & Francis Group, LLC.
This is an Open Access article distributed under the terms of the Creative Commons Attribution License (http://creativecommons.org/licenses/by/4.0/), which permits unrestricted use, distribution, and reproduction in any medium, provided the original work is properly cited.

### Migration of Online Services in a Community-Based Pain Clinic Catalyzed by the COVID-19 Pandemic

Brenda Lau[a] and Emmanuel Abreu[b]

[a]CHANGEpain Clinic, Medical Director, Vancouver, British Columbia, Canada; [b]CHANGEpain Clinic, Research Director, Vancouver, British Columbia, Canada

The use of telehealth dramatically accelerated because of concerns for patient and healthcare provider safety during the SARS-CoV2-19 (COVID-19) pandemic. CHANGEpain's interdisciplinary online services highlighted important issues and learnings regarding the benefits and challenges for chronic pain patients. To examine the impact of onsite occupancy ratios and expanding virtual services, appointment numbers were analyzed between March to September 2020 at the height of COVID workflow changes and compared to the same period in 2019. While some services were largely unchanged or reduced, some experienced over 23000% increase in use.

**Introduction/Aim**: The following summarizes our experience in the transformation of our services with onsite and virtual services. the aims for this research were (1) To study the increase of reach in CHANGEpain Clinic regarding the treatment of chronic pain (2) To understand the number of appointments offered to CPC patients per week as the pandemic evolved.

**Methods**: The electronic medical record Input Health Acted as a database. Appointment volumes were analyzed using pre-defined criteria by the principal investigator and research director. The medical appointment types examined were onsite visits, individual and group visits and emails. The data was mined using the combination of billings data and the data-export comma- separated value format (.csv). This report was then imported in Statistical Package for the Social Sciences (SPSS) V.27, categories were drawn, and frequency tables were made. An algorithm was created to understand which patients could be seen through this modality.

**Results**: New patient consults were reduced by 15% while follow-ups increased by 65%. On-site procedures increased by 14% from 6240 to 7110, as onsite rooms were not used for consultations. Group Medical Visits substantially increased by 705% from 1230 visits to 9900 as up to 76 sessions were offered instead of 4 per week. There was a 180% increase in physician to healthcare provider phone support and 23,150% increase in email reach as part of patient care from 4 to 930 interventions.

**Discussion/Conclusions**: Services such as consults, follow-ups are potentially more accessible by virtual care and certain patients utilize group medical visits more than others. Patients noted that virtual individual and group therapies were deemed pivotal in reducing hopelessness and sense of isolation and they helped save time. These observations demonstrate that providing a mix of onsite and virtual care increased the reach of multi-disciplinary care. Further studies are needed to understand the economical repercussions, sustainability and long-term effectiveness of virtual access for patients with chronic pain and comorbid conditions such as mental health, sleep and movement dysfunction.

### ORCID

Brenda Lau http://orcid.org/0000-0002-0090-016X
Emmanuel Abreu http://orcid.org/0000-0002-0295-214X

© 2021 The Author(s). Published with license by Taylor & Francis Group, LLC.
This is an Open Access article distributed under the terms of the Creative Commons Attribution License (http://creativecommons.org/licenses/by/4.0/), which permits unrestricted use, distribution, and reproduction in any medium, provided the original work is properly cited.

### Developing a Better Measure of Helplessness for Chronic Conditions

Lindsey Yessick and Tim Salomons

Queen's University, Psychology, Kingston, Ontario, Canada

**Introduction/Aim**: As helplessness develops with exposure to repeated stressors and no perceived viable control strategy, helplessness is relevant to chronic pain (CP) populations. This study has developed a helplessness scale for chronic conditions distinct from previous scales that blur the conceptualization of control constructs. Extant measures commonly examine controllability, not the three pillars of helplessness identified by Maier and Seligman (1976): cognitive, emotional, and motivational/motor deficits.

**Methods**: Individuals who self-report a CP condition (N = 350) or diabetes (N = 36) responded to a Chronic Disease Helplessness Survey (CDHS) constructed to capture cognitive, motor, and emotion deficits. Exploratory factor analysis (EFA; N = 200) and confirmatory factor analysis (CFA; N = 150) we performed. The CDHS was assessed for convergent and discriminant (comparison of CP to diabetes) validity.

**Results**: Following EFA, a three-factor solution of the CDHS was selected. The three-factor model exhibited sufficient model fit on two of three indices. The cognitive (α = .812), emotional (α = .762), and motor (α = .909) factors exhibited satisfactory reliability. The CDHS was significantly associated with greater pain intensity ($r$ = .377, $p < .001$) and interference ($r$ = .604, $p < .001$), PCS helplessness ($r$ = .620, $p < .001$), and lower general self-efficacy (r = -.386, $p < .001$). Individuals with diabetes displayed significantly lower CDHS scores in comparison to individuals with CP $t(384) = 3.44$, $p = .001$; $d = 0.59$.

**Discussion/Conclusions**: This study provides preliminary evidence that the three-factor CDHS is psychometrically a sound measure of CP helplessness.

© 2021 The Author(s). Published with license by Taylor & Francis Group, LLC.
This is an Open Access article distributed under the terms of the Creative Commons Attribution License (http://creativecommons.org/licenses/by/4.0/), which permits unrestricted use, distribution, and reproduction in any medium, provided the original work is properly cited.

### Coping responses and pediatric chronic pain: Uncovering the need for theory, conceptual clarity, and conceptual consistency

A. Natisha Nabbijohn[a], Rachel Tomlinson[a], Soeun Lee[a], Barbara Morrongiello[a], and C. Meghan McMurtry[b]

[a]University of Guelph, Clinical Child and Adolescent Psychology, Guelph, Ontario, Canada; [b] University of Guelph, Clinical Child and Adolescent Psychology, Guelph; Pediatric Chronic Pain Program, McMaster Children's Hospital, Hamilton, Ontario, Canada

**Introduction/Aim**: Coping is a crucial aspect of pediatric chronic pain management. Inconsistent conceptualizations and unclear descriptions of coping responses are barriers to consolidating and interpreting findings. Within a larger scoping review, this poster will: 1) summarize the use of theoretical frameworks to guide the measurement of coping responses; 2) evaluate the use descriptors (i.e., definitions/examples) to explain the copings responses assessed (conceptual clarity); and 3) evaluate the consistency of descriptions used with the original scale (conceptual consistency).

**Methods**: Descriptors and theoretical frameworks of coping responses were mapped using a scoping review methodology. Searches were conducted through MEDLINE (PubMed) and PsycINFO. Following title/abstract screening, full-text extractions were performed on 125 English-language publications on coping in youth with chronic pain.

**Results**: Only 12.8% ($n$ = 16) of studies used a theoretical framework to explain the coping responses assessed and fewer ($n$ = 9) used theory to guide measure selection. Conceptual clarity was rated "low/very low" for 47.2% ($n$ = 59) (i.e., no definitions and/or examples). For conceptual consistency, 33.7% ($n$ = 32) of studies were rated "low" (i.e., one or more discrepancies) and 30.5% ($n$ = 29) were rated as "unclear" (i.e., no descriptors to compare).

**Discussion/Conclusions**: This review highlights the lack of clear descriptions and theoretical frameworks of coping responses for pediatric chronic pain. As a field, we need to strive towards using more explicit and well-developed descriptions and theories of coping responses to serve as an essential knowledge base for construct validation, synthesizing research findings, and knowledge translation.

### ORCID

C. Meghan McMurtry http://orcid.org/0000-0002-3278-1169

© 2021 The Author(s). Published with license by Taylor & Francis Group, LLC.
This is an Open Access article distributed under the terms of the Creative Commons Attribution License (http://creativecommons.org/licenses/by/4.0/), which permits unrestricted use, distribution, and reproduction in any medium, provided the original work is properly cited.

### The known unknowns: A scoping review of literature on family physicians' gaps in knowledge and skills for managing chronic pain

Mihal Esterlis[a], Virginia McEwen[b], and Anuj Bhatia[c]

[a]Ryerson University, Nursing, Toronto, Ontario, Canada; [b]McGill University, Alan Edwards Pain Management, Montreal, Quebec; [c]Toronto Western Hospital- University Health Network, Department of Anesthesia and Pain Management, Toronto, Ontario, Canada

**Introduction/Aim**: Primary care providers (PCPs), who provide the bulk of care for patients with chronic noncancer pain (CNCP), often report knowledge gaps, limited resources, and difficult patient encounters while managing chronic pain. This scoping review seeks to evaluate the extent of PCPs' identified knowledge/skill gaps in chronic pain management.

**Methods**: A broad literature search was conducted for relevant articles in the area of knowledge and skill gaps of PCP for managing chronic pain, with multiple search term derivatives of both concepts. The initial search yielded 182 articles which were then screened for relevance, yielding 23 studies.

**Results**: The 23 articles included in this review reflected a variety of study designs, settings, and methods. Consistent themes emerged with respect to knowledge gaps in assessment, diagnosis, treatment, and allied health roles in chronic pain. A general lack of confidence in approaching discontinuation of high dose or ineffective opioid regimes, professional isolation and managing complex pain patients with limited access to pain specialists were also reported.

**Discussion/Conclusions**: Studies summarized in this review demonstrate commonalities that will be useful in creating a targeted, useful and concise curriculum for primary care providers. Findings from these studies will be used to generate a curriculum of priority topics for PCPs caring for chronic noncancer pain patients. The current scoping review synthesizes information from clinical studies that can inform us in creating a Pain Day for PCPs that will maximize learning and ultimately benefit chronic pain patients.

### ORCID

Mihal Esterlis http://orcid.org/0000-0002-1249-0671

© 2021 The Author(s). Published with license by Taylor & Francis Group, LLC.
This is an Open Access article distributed under the terms of the Creative Commons Attribution License (http://creativecommons.org/licenses/by/4.0/), which permits unrestricted use, distribution, and reproduction in any medium, provided the original work is properly cited.

**Table 1.** A summary of types of studies that assessed primary care providers' deficits in knowledge and skills for chronic pain management.

| Study Type | Number |
|---|---|
| Qualitative study design | 9 |
| Randomized control trial | 1 |
| Cross sectional survey | 10 |
| Mixed methods | 3 |

### Motor control of the spine in pregnancy-related lumbopelvic pain: a systematic review

Desgagnés Amélie[a], Patricio Philippe[a], Bérubé Noémie[a], Lamothe Mélanie[b], Bernard Stéphanie[b], and Massé-Alarie Hugo[a]

[a]Centre interdisciplinaire de recherche en réadaptation et en intégration sociale (CIRRIS), Rehabilitation Department, Quebec City, Quebec, Canada; [b]Centre interdisciplinaire de recherche en réadaptation et en intégration sociale (CIRRIS), Rehabilitation Department, Quebec City, Quebec, Canada

**Introduction/Aim**: Some studies observed differences in motor control of the spine between women with pregnancy-related lumbopelvic pain (PLPP) and matched controls, although not all. The aim of this study was to perform a systematic review of the literature to determine if there are consistent differences in motor control of the spine in women with PLPP compared to matched controls.

**Methods**: Five databases were searched (MEDLINE, EMBASE, CINAHL, Web of Science and Evidence-Based Medicine Reviews). Observational studies comparing motor control of the lumbopelvic spine between PLPP subjects and matched controls were included. Risk of bias was assessed with a modified version of STROBE statement for cross-sectional studies.

**Results**: Fifteen studies were included. Pregnant women with PLPP showed some differences in lumbar spine kinematic during walking and lifting. Higher *transversus abdominis muscle* (TrA) activation during leg movements in post-partum PLPP compared to controls was present but difference in pelvic floor muscle function was inconsistent. Large heterogeneity in variables, tasks and results limited the analysis.

**Discussion/Conclusions**: This systematic review identified multiple differences in motor control in PLPP population compared to matched controls, predominantly in dynamic tasks. Results also suggest the importance of assessing different muscles in multiple tasks when evaluating motor control in women with PLPP. Giving the high proportion of women afflicted by PLPP and the lack of understanding of underlying mechanisms, this review highlights the need to deepen research in motor control in this specific population to optimize interventions.

© 2021 The Author(s). Published with license by Taylor & Francis Group, LLC.
This is an Open Access article distributed under the terms of the Creative Commons Attribution License (http://creativecommons.org/licenses/by/4.0/), which permits unrestricted use, distribution, and reproduction in any medium, provided the original work is properly cited.

### Risk of Bias Assessment in Epidemiological Studies of Chronic Pain in Children and Adolescents

Charlotte L. Langley[a], Perri R. Tutelman[b], Jennifer A. Parker[a], Christine T. Chambers[c], Brittany Cormier[a], Nicole A. Proudfoot[d], Darlene Chapman[e], G. Allen Finley[a], Gareth T. Jones[f], Gary J. Macfarlane[f], and Justina Marianayagam[g]

[a]IWK Health Centre, Centre for Pediatric Pain Research, Halifax, NS, Canada; [b]Dalhousie University, Department of Psychology and Neuroscience, Halifax, NS, Canada; [c]Dalhousie University, Departments of Psychology and Neuroscience and Pediatrics, Halifax, NS, Canada; [d]Nicole A. Proudfoot, PhD, IWK Health Centre, Centre for Pediatric Pain Research, Halifax, NS, Canada; [e]IWK Health Centre, Health Sciences Library, Halifax, NS, Canada; [f]University of Aberdeen, Epidemiology Group and Aberdeen Centre for Arthritis and Musculoskeletal Health, Aberdeen, Aberdeen, UK; [g]Northern Ontario School of Medicine, Thunder Bay, ON, Canada

**Introduction/Aim**: Risk of bias (RoB) assessment is an important component of a systematic review, as the review's results depend on the quality of the primary studies included. As part of a systematic review update, we assessed the RoB in epidemiological studies examining the prevalence of chronic pain in children and adolescents.

**Methods**: 130 studies were assessed for RoB using a 10-item tool developed by Hoy et. al, (2012). Four domains of bias were evaluated: selection bias (Items 1-3), nonresponse bias (Item 4), measurement bias (Items 5-9), bias related to the analysis (Item 10). Response options for each item are "0" (low risk) or "1" (high risk). A summary score was calculated for each study. Each study's overall RoB was categorized as low (0-2), moderate (3-4), or high (5-10).

**Results**: Overall, 52 (40.0%) studies were categorized as having a low RoB, 68 (52.3%) were of moderate risk, and 10 (7.7%) had a high risk. Lack of national representativeness (Item 1) and nonresponse bias (Item 4) were the most common areas of bias across studies (81.5% and 56.2%, respectively). Consistent data collection (Item 8) was the least common with no studies scoring as high risk in this area.

**Discussion/Conclusions**: Although most studies had low or moderate overall RoB, only one study was rated as low risk in all 10 areas. Confidence in the estimate of chronic pain prevalence in children and adolescents would be improved in future studies by the adoption of designs that include nationally representative samples and minimize nonresponse bias.

### ORCID

Charlotte L. Langley http://orcid.org/0000-0002-0291-8734
Perri R. Tutelman http://orcid.org/0000-0001-6013-7402
Jennifer A. Parker http://orcid.org/0000-0001-9900-4703
Christine T. Chambers http://orcid.org/0000-0002-7138-916X
G. Allen Finley http://orcid.org/0000-0003-4579-7749
Gareth T. Jones http://orcid.org/0000-0003-0016-7591
Gary J. Macfarlane http://orcid.org/0000-0003-2322-3314

© 2021 The Author(s). Published with license by Taylor & Francis Group, LLC.
This is an Open Access article distributed under the terms of the Creative Commons Attribution License (http://creativecommons.org/licenses/by/4.0/), which permits unrestricted use, distribution, and reproduction in any medium, provided the original work is properly cited.

### Knowledge, Beliefs, and Attitudes of Emergency Nurses Toward People With Chronic Pain

Geraldine Martorella[a], Michelle Kostic[b], Anaïs Lacasse[c], Glenna Schluck[a], and Laurie Abbott[a]

[a]TMH Center for Research and Evidence-Based Practice, College of Nursing, Florida State University,Tallahassee, FL, USA; [b]College of Nursing, Florida State University, Tallahassee, FL, USA; [c]Laboratoire de recherche en épidémiologie de la douleur chronique Département des sciences de la santéUniversité du Québec en Abitibi-Témiscamingue, Rouyn-Noranda, Quebec, Canada

**Introduction/Aim**: People with chronic pain (CP) visit more and more the emergency department (ED) and report unmet needs. Although stigmatization could partially explain this gap, most studies have been focused on nurses' pain management knowledge. Hence, understanding of the determinants of the stigma related to CP is needed. The objectives were to 1) describe the knowledge, beliefs and attitudes of ED nurses toward people with CP, and 2) identify nurses' characteristics associated with these perceptions.

**Methods**: A cross-sectional Web-based survey design was conducted using the KnowPain-12 questionnaire and the Chronic Pain Myth Scale.

**Results**: 571 participants from twenty states across the United States responded, among whom 482 completed the entire survey. The sample included about one third of ED nurses suffering from CP. Negative beliefs and attitudes toward people with CP were present in a considerable proportion of participants (up to 64%), even in nurses suffering from CP (up to 47.5%). Nevertheless, our results suggest that higher levels of education ($p$=.01) and suffering from CP ($p$<.001) were associated with better beliefs and attitudes toward people with CP.

**Discussion/Conclusions**: The ED presents a risk of stigmatization of people with CP. Identifying determinants of the stigma associated with CP is crucial for tailoring awareness campaigns. Additionally, CP patients utilizing the ED have complex needs which are difficult to address in this clinical environment, thus contributing to negative perceptions. Given the scarcity of specialized care for this population, healthcare stakeholders should devise solutions to improve continuity of care between primary care settings and the ED.

### ORCID

Geraldine Martorella http://orcid.org/0000-0001-9383-7114
Anaïs Lacasse http://orcid.org/0000-0002-3992-5145
Glenna Schluck http://orcid.org/0000-0001-5311-9636
Laurie Abbott http://orcid.org/0000-0002-4017-1416

© 2021 The Author(s). Published with license by Taylor & Francis Group, LLC.
This is an Open Access article distributed under the terms of the Creative Commons Attribution-NonCommercial License (http://creativecommons.org/licenses/by-nc/4.0/), which permits unrestricted non-commercial use, distribution, and reproduction in any medium, provided the original work is properly cited.

### Clusters of conditioned pain modulation responses in a large pediatric sample with chronic pain

Don Daniel Ocay[a], Stéphane Potvin[b], Serge Marchand[c], and Catherine E. Ferland[d]

[a]Department of Experimental Surgery, Faculty of Medicine and Health Sciences, McGill University, Montreal, Canada; [b] Department of Psychiatry, Faculty of Medicine, Université de Montréal, Montreal, Canada; [c]Centre de recherche du Centre Hospitalier de l'Université de Sherbrooke, Sherbrooke, Canada; [d]Department of Anesthesia, Faculty of Medicine and Health Sciences, McGill University, Montreal, Canada

**Introduction/Aim**: Due to the heterogeneity and role of endogenous facilitatory and inhibitory pain mechanisms such as conditioned pain modulation (CPM) efficiency and temporal summation within the different chronic pain populations, it is important to take both into consideration in a single experimental model to give as much information as possible on subgroups of patients. Therefore, the objective of this study was to identify subgroups in a large population of pediatric patients with chronic pain based on their endogenous facilitatory and inhibitory pain mechanisms and compare them with healthy controls.

**Methods**: Six hundred and eight patients and sixty age-matched healthy controls underwent a CPM assessment using tonic noxious heat stimulation as the test stimulus and the cold pressor task (CPT) as the conditioning stimulus.

**Results**: The best partition of clusters of the patient sample was three clusters accounting for 27.15% of the total variation in the data. Cluster 1 (n = 271) was best characterized by high levels of pain intensity during the cold pressor task (CPT), lack of temporal summation during the test stimuli, and efficient inhibitory CPM.

Cluster 2 (n = 186) was best characterized by low levels of pain intensity during the CPT, lack of temporal summation during the test stimuli, and efficient inhibitory CPM. Cluster 3 (n = 151) was best characterized by high levels of pain intensity during the CPT, presence of temporal summation during the test stimuli, and inefficient inhibitory CPM.

**Discussion/Conclusions**: In conclusion, a single thermal CPM experimental design can identify multiple combinations of excitatory and inhibitory pain mechanisms. Findings from the current study add to the literature by describing different clinical phenotypes of central pain mechanisms of youth with chronic pain.

© 2021 The Author(s). Published with license by Taylor & Francis Group, LLC.
This is an Open Access article distributed under the terms of the Creative Commons Attribution License (http://creativecommons.org/licenses/by/4.0/), which permits unrestricted use, distribution, and reproduction in any medium, provided the original work is properly cited.

### Investigating the Relationship Between Medical Cannabis Use and Biomarkers Among Adult Chronic Pain Patients

Prabjit Ajrawat[a], Yi Yang[b], Ewa Wasilewski[c], Timothy Leroux[d], Mohit Kapoor[d], Lakshmi P. Kotra[b], and Hance Clarke[e]

[a]University of Toronto Temerty Faculty of Medicine, Department of Anesthesia, Toronto General Hospital, Toronto, ON, Canada; [b]Leslie Dan Faculty of Pharmacy, University of Toronto, Toronto, Ontario, Canada; [c]Krembil Research Institute, University Health Network, Toronto, Ontario, Canada; [d]Osteoarthritis Research Program, Division of Orthopedics, Schroeder Arthritis Institute, University Health Network, Toronto, Ontario, Canada; [e]Director Pain Services / Toronto General Hospital, Department of Anesthesiology and Pain Medicine, University of Toronto, Toronto, Ontario, Canada

**Introduction/Aim**: Cannabis is a potential therapeutic option for some chronic pain (CP) patients. However, investigations of cannabis' potential immunomodulatory effects in humans with CP remain scarce. We sought to characterize the relationship between cannabis use and biomarkers in patients legally authorized cannabis (pre-2018) among CP patients.

**Methods**: Adult CP patients with medical authorization of cannabis with a registered licensed producer in Canada were enrolled. Patients completed questionnaires while providing blood and cannabis samples. Blood plasma samples were analyzed for the presence of 1) cannabinoids and their metabolites using UPLC/MS and 2) 29 different cytokines/chemokines and cortisol using Milliplex® assay platforms. Descriptive statistics were utilized to summarize demographic, clinical, and blood sample variables.

**Results**: Fifty-six patients (48±15 years; 64% females; 52% arthritic conditions) were included with dried cannabis (53%) being the most common type of cannabis consumed. Seventy percent of products were considered THC dominant (daily dose:1.7±1.3 grams). Majority of participants (96%) reported effective pain management and 76% self-reported a decrease in other analgesic medication usage. Females tended to have greater concentrations of IL-1Ra, IL-4, IL-1α, and IFNα2 when compared to males. Patients using CBD products had lower concentrations of IL-1Ra, IL-4, IL-6, IL-15, IL-12p40, IFNα2, MCP-1, VEGF, EGF, TNF-α, TNF-β, and cortisol when compared to THC products.

**Conclusions**: Given the patient-perceived effectiveness of cannabis to manage CP symptoms, the above biomarkers suggest a potential immunomodulatory effect associated with CBD based products. This preliminary data needs further validation by using larger well-controlled studies.

© 2021 The Author(s). Published with license by Taylor & Francis Group, LLC.
This is an Open Access article distributed under the terms of the Creative Commons Attribution License (http://creativecommons.org/licenses/by/4.0/), which permits unrestricted use, distribution, and reproduction in any medium, provided the original work is properly cited.

### Exercise-induced hypoalgesia in chronic low back pain subgroups stratified by pain mechanisms

Catherine Mailloux[a], Timothy H. Wideman[b], Pierre Langevin[a], Nathalie Mathieu[c], Martin Descarreaux[d], David Walton[e], Louis-David Beaulieu[f], and Hugo Massé-Alarie[a]

[a]Centre interdisciplinaire de recherche en réadaptation et intégration sociale (Cirris), Université Laval, Quebec City, QC, Canada; [b]School of Physical and Occupational Therapy, McGill University, Montreal, QC, Canada; [c]Centre Interdisciplinaire en Gestion de la Douleur (CIGD), Hôpital Hôtel-Dieu de Lévis, Centre intégré de santé et de services sociaux de Chaudière-Appalaches (CISSSCA), Lévis, QC, Canada; [d]Département des sciences de l'activité physique, Université du Québec à Trois-Rivières, Trois-Rivières, QC, Canada; [e]School of physical therapy, Western University, London, ON, Canada; [f]BioNR Research Lab, Université du Québec à Chicoutimi, Chicoutimi, QC, Canada

**Introduction/Aim**: The use of pain mechanism-based classification (e.g. nociceptive vs. nociplastic) is promising to improve the intervention effect. Exercise reduces pain sensitivity (exercise-induced hypoalgesia [EIH]) and may explain the beneficial effect of back exercises in chronic low back pain (CLBP). Since (i) an alteration of the efficacy of the pain modulation system has been suggested in nociplastic pain conversely to nociceptive pain and (ii) the pain modulation system is involved in EIH, people with nociplastic pain may respond less to exercise than those with nociceptive pain. The objectives were to compare EIH between nociceptive and nociplastic subgroups, and pain-free participants, and to compare CLBP subgroups characteristics (e.g. psychological factors).

**Methods**: Sixteen participants with nociceptive CLBP, 12 with a component of nociplastic CLBP and 24 pain-free participants were recruited. Participants performed wrist and back exercises. Pressure pain threshold was assessed pre/post-exercise, and percentage of change (%PPT) was measured. Questionnaires measuring disability, "central sensitization", anxiety, self-efficacy, fear of movement and coping strategies were also completed by participants suffering CLBP.

**Results**: The subgroup with a nociplastic component presented lower %PPT compared to HC. It also presented higher level of physiological anxiety, lower level of self-efficacy and used more coping strategies compared to the nociceptive subgroup.

**Discussion/Conclusions**: Our results suggest that a subgroup with a component of nociplastic pain presents an alteration in pain processing related to exercise and a different clinical profile than the nociceptive subgroup.

### ORCID

Louis-David Beaulieu http://orcid.org/0000-0003-4831-2380

© 2021 The Author(s). Published with license by Taylor & Francis Group, LLC.
This is an Open Access article distributed under the terms of the Creative Commons Attribution License (http://creativecommons.org/licenses/by/4.0/), which permits unrestricted use, distribution, and reproduction in any medium, provided the original work is properly cited.

### Antibodies from fibromyalgia patients induce pain-related behaviours in mice

Emerson Krock[a], Alexandra Jurczak[a], Carlos Morado Urbina[a], Katalin Sandor[a], Clive Gentry[b], Mathilde R. Israel[b], Nisha Vastani[b], Ulku Cuhadar[b], Margot Maurer[b], Serena Sensi[c], Yuki Nomura[a], Joana Menezes[a], Azar Baharpoor[a], Louisa Brieskorn[a], Angelica Sandström[d], Jeanette Tour[d], Diana Kadetoff[d], Lisbet Haglund[e], Eva Kosek[d], Stuart Bevan[b], Andreas Goebel[c], David A. Andersson[b], and Camilla I. Svensson[a]

[a]Karolinska Institutet, Department of Physiology and Pharmacology, Stockholm, Sweden; [b]King's College London, Wolfson CARD, Institute of Psychiatry, Psychology & Neuroscience, Guy's Campus, London, England; [c]University of Liverpool, Pain Research Institute, Department of Translational Medicine, Liverpool, England; [d]Karolinska Institutet, Department of Clinical Neuroscience, and the Stockholm Spine Centre, Stockholm, Sweden; [e]McGill University, Department of Surgery, Montreal, Canada

**Introduction/Aim**: The underlying pathophysiology of fibromyalgia (FM) is unknown, but FM is often comorbid with autoimmune diseases. We hypothesized that IgG contributes to FM symptoms.

**Methods**: Purified IgG from primary FM patients and aged-matched controls (HC) was injected into mice and we assessed paw pressure (Randall-Selitto) and punctate (von Frey) mechanical sensitivities and locomotor activity (CLAMS). IgG localization following injection, satellite glia cell (SGC) reactivity and intraepidermal nerve fiber density were assessed with immunofluorescence. Mouse dorsal root ganglia (DRG) cell cultures were used to confirm FM IgG binding profiles. IgG autoreactivity was examined by immunofluorescence in human DRGs.

**Results**: Transfer of FM IgG, but not HC IgG, induced hypersensitivity to pressure and punctate mechanical stimulation and reduced locomotor activity. FM IgG accumulated in the DRG, but not the brain or spinal cord, and colocalized primarily with SGCs and to some extent with neurons. SGCs displayed signs of increased activity in FM IgG injected mice and FM IgG immunoreactivity was greater in cultured SGCs and neurons compared to HC IgG. Intraepidermal nerve fiber density was decreased in hind paw glabrous skin of FM IgG-injected mice. Finally, we found that FM IgG binds to human DRG tissue, including SGCs and neurons, more intensely than HC IgG.

**Discussion/Conclusions**: FM IgG transfer drives evoked and non-evoked pain-like behaviour and pain-related neuroanatomical alterations while binding mouse and human SGCs and neurons. These findings suggest that FM patients have autoreactive antibodies that generate a subset of FMS symptoms, thus opening the door to antibody targeting therapies.

© 2021 The Author(s). Published with license by Taylor & Francis Group, LLC.
This is an Open Access article distributed under the terms of the Creative Commons Attribution License (http://creativecommons.org/licenses/by/4.0/), which permits unrestricted use, distribution, and reproduction in any medium, provided the original work is properly cited.

### A Retrospective Cohort Study of Healthcare Utilization Following Paravertebral Blocks in Ontario, Canada

George Deng[a], Michael Gofeld[b], Jennifer N Reid[c], Blayne Welk[d], Anne MR Agur[e], and Eldon Loh MD[f]

[a]Vivo Cura Health, Calgary, Alberta, Canada; [b]Silver Pain Care, Toronto, Ontario, Canada; [c]Ontario, Canada; [d]Department of Surgery, Western University, London, Ontario, Canada; [e]Division of Anatomy, Department of Surgery, University of Toronto, Toronto, Ontario, Canada; [f]Department of Physical Medicine and Rehabilitation, Western University, London, Ontario, Canada

**Introduction/Aim**: Paravertebral blocks (PVBs) are frequently performed in Ontario, Canada for the management of chronic pain despite limited evidence. Although these procedures are suspected to be resource intensive, their impact on healthcare utilization has not been formally evaluated. The goal of this study is to assess the effect of PVBs on physician visits, the need for other interventional procedures, and opioid prescribing.

**Methods**: A retrospective cohort study was conducted in Ontario, Canada, between July 1, 2013 and March 31, 2018, using administrative data from the ICES database. Ontario residents who received an initial PVB in this timeframe were included. Use of other interventions, physician visits, and opioid doses were compared in the 12-month periods before and after index PVBs.

**Results**: 47,723 patients received their initial PVBs in the study period. Family physicians (N=25,042) and anesthesiologists (N=14,195) performed the highest number of index PVBs. 12,474 patients (26.15%) received 10 or more repeat PVBs in the 12-month period after index PVB. The number of non-image guided interventions (excluding PVBs) received per patient (mean±SD) increased from 2.19±9.35 to 31.68±52.26 in the 12 months before and after index PVBs. Physician visits per patient (mean±SD) increased from 2.92±3.61 to 9.64±11.77 in the same timeframes. Mean opioid dosing did not change significantly (13.01± 32.03 mg pre; 13.17 ± 30.61 mg post).

**Discussion/Conclusions**: PVBs are associated with a significant increase in healthcare utilization and no change in opioid dosing. Their routine use should be re-evaluated to ensure appropriate allocation of healthcare resources.

© 2021 The Author(s). Published with license by Taylor & Francis Group, LLC.
This is an Open Access article distributed under the terms of the Creative Commons Attribution License (http://creativecommons.org/licenses/by/4.0/), which permits unrestricted use, distribution, and reproduction in any medium, provided the original work is properly cited.

### Preliminary acceptability of a patient-oriented music intervention (POMI) among clinicians and music experts for pain management in critically ill adults

Melissa Richard-Lalonde[a], Linda L. Chlan[b], Sylvie Cossette[c], Nancy Feeley[d], and Céline Gélinas[d]

[a]McGill University, Ingram School of Nursing, Montreal, Quebec, Canada; [b]Mayo Clinic, Rochester, MN, USA; [c]SUniversité de Montréal, Nursing and Montreal Heart Institute, Montreal, QC, Canada; [d]McGill University, Ingram School of Nursing and Jewish General Hospital, Montreal, QC, Canada

**Introduction/Aim**: Multimodal approaches for pain management in the critically ill adult are recommended. Music listening can effectively reduce pain; however, existing interventions are time- and resource-consuming. A patient-oriented music intervention (POMI) was developed based on theoretical and empirical knowledge. This study aims to describe the POMI acceptability through experiential knowledge from ICU clinicians and music experts for the refinement of the POMI.

**Methods**: A mixed-method study is being conducted to determine the acceptability of the POMI. Ten ICU clinicians and music experts with at least two years of experience are being recruited using a purposive and snowball sampling strategy. Individual Zoom meetings are held where the POMI is presented to participants, who then answer a 6-item questionnaire and interview questions on acceptability. Acceptability is evaluated on six attributes, each rated from 0 (not acceptable) to 4 (most acceptable).

**Results**: So far, six participants have been recruited (two music therapists, three nurses and one physician). All acceptability attributes had a median of ≥3. In addition to asking the patient's preferred music, participants suggested considering music that the patient dislikes and requested a pre-programmed playlist for patients who are alone and unable to express their preferences. Participants spontaneously reported using music with COVID-19 patients, due to the increased isolation in the ICU.

**Discussion/Conclusions**: The POMI appears acceptable. Refinements (e.g., allowing specific music to be excluded) to the POMI will be made in preparation for pilot testing. Additionally, the POMI could provide a comforting ICU environment during the pandemic.

© 2021 The Author(s). Published with license by Taylor & Francis Group, LLC.
This is an Open Access article distributed under the terms of the Creative Commons Attribution License (http://creativecommons.org/licenses/by/4.0/), which permits unrestricted use, distribution, and reproduction in any medium, provided the original work is properly cited.

### Sensitivity to pain traumatization and its relationship to the stress-pain connection in youth with chronic pain

Larah Maunder[a], Melanie Noel[b], Joel Katz[c], Maria Pavlova[d], Jaimie K. Beveridge[b], and Tim Salomons[a]

[a]Queen's University, Psychology, Kingston, Ontario, Canada; [b]University of Calgary, Psychology, Calgary, Alberta, Canada; [c]York University, Psychology, Toronto, Ontario, Canada; [d]MSc, University of Calgary, Psychology, Calgary, Alberta, Canada

**Introduction/Aim**: Previous studies have identified psychological factors that exacerbate chronic pain. Recently, Sensitivity to Pain Traumatization (SPT), the propensity for an individual to develop posttraumatic emotional responses to pain, has been proposed as one factor that magnifies chronic pain. This study investigated this hypothesis by examining whether SPT exacerbates chronic pain in children.

**Methods**: Using longitudinal self-report data from youth aged 10-18 years seen at a pediatric chronic pain clinic, we conducted a multiple linear regression to investigate whether SPT moderated the relationship between anxiety levels at baseline and pain levels three months later (*criterion variable*: pain intensity at 3 months; *predictor variables*: average baseline anxiety level over a 7-day period, SPT severity at baseline; *covariate*: baseline pain intensity).

**Results**: SPT significantly moderated the relationship between anxiety level and pain intensity. Simple-effects showed that as anxiety level increased, those high on SPT (i.e., 1 SD above mean SPT score) reported a more pronounced increase in pain intensity level. Conversely, those low on SPT (i.e., 1 SD below mean SPT score) reported decreased levels of pain intensity as mean anxiety level increased. Johnson-Neyman analyses indicated that the relationship between anxiety level and pain intensity became significant when SPT score was 21.45 (out of 48) or higher.

**Discussion/Conclusions**: High SPT moderates the relationship between anxiety and pain, such that children who experience their pain as highly traumatizing are more likely to experience high pain intensity when their anxiety levels are high than are children who do not experience their pain as traumatizing.

### ORCID

Melanie Noel http://orcid.org/0000-0003-3752-8055
Joel Katz http://orcid.org/0000-0002-8686-447X

© 2021 The Author(s). Published with license by Taylor & Francis Group, LLC.
This is an Open Access article distributed under the terms of the Creative Commons Attribution License (http://creativecommons.org/licenses/by/4.0/), which permits unrestricted use, distribution, and reproduction in any medium, provided the original work is properly cited.

### Use of technology for chronic pain management: experiences of adults and Occupational Therapists

Tamara Wong[a], Kellie Lothiaphing[a], Shital Desai[b], and Arlene Astell[c]

[a]University of Toronto, Occupational Therapy, Toronto, Ontario, Canada; [b]York University, School of the Arts Media Performance & Design, Toronto, Ontario, Canada; [c]Toronto Rehabilitation Institute – KITE Research Institute, DATE Lab, Toronto, Ontario, Canada

**Introduction/Aim**: Chronic pain (CP) is a prominent health issue that can negatively affect individuals' well-being and ability to engage in activities of daily living. With technology evolving rapidly, there is an increased interest in using technology to manage CP. The objectives of this study are to (i) determine the factors influencing adults' use of technology for CP management and (ii) explore the experiences of Occupational Therapists (OTs) who use technology to help patients with CP.

**Methods**: For this qualitative descriptive study, online semi-structured interviews were conducted with adults (over 18 years old) that self-identify with CP to gather their experiences with CP and technology. OTs were interviewed to discuss their use of technology to support adults managing CP. No formal definition of technology was provided, allowing participants to use their interpretation of technology (i.e. it did not have to be digital).

**Results**: Preliminary Thematic Analysis indicates that adult participants use a diverse range of technologies for self-management of CP, reflecting their experience of pain as highly individualized. OTs typically use technology as a form of education to teach and reinforce CP self-management strategies. Common factors among adults with CP and OT participants for liking technology to manage CP were convenience, simplicity and effectiveness.

**Discussion/Conclusions**: Participants' experiences obtained from this study will help establish knowledge of the types of technology used and the design features that are effective for CP self-management technology.

### ORCID

Shital Desai http://orcid.org/0000-0003-1851-0597

© 2021 The Author(s). Published with license by Taylor & Francis Group, LLC.
This is an Open Access article distributed under the terms of the Creative Commons Attribution License (http://creativecommons.org/licenses/by/4.0/), which permits unrestricted use, distribution, and reproduction in any medium, provided the original work is properly cited.

### How chronic pain patients determine meaningfulness in their rehabilitation: a phenomenological study

Katrina J. Liddiard[a], Annette J. Raynor[b], Helen M. DeJong[c], and Cary A. Brown[d]

[a]Edith Cowan University, School of Medical & Health Science, Joondalup, Western Australia, Australia; [b]Annette J. Raynor, PhD, Edith Cowan University, School of Medical & Health Science, Joondalup, Western Australia, Australia; [c]Helen M. DeJong, PhD, Perth Scar & Pain Clinic, Mount Pleasant, Western Australia, Australia; [d]Cary A. Brown, PhD, University of Alberta, Faculty of Rehabilitation Medicine, Edmonton, Alberta, Canada

**Purpose**: People with chronic pain often seek occupational or physical therapy to reduce pain or restore function and wellbeing. In theory patients perceive therapy to be more meaningful if a biopsychosocial approach is adopted, however, much of the literature only describes meaningful rehabilitation from the perspective of the therapist or researcher, and little is known about how people with pain determine meaningfulness. This phenomenological study investigated meaningfulness in rehabilitation from the perspective of people with chronic pain.

**Methods**: A purposive sample of ten Australian adults with chronic pain, and who recent completed occupational or physical therapy, were recruited. Semi-structured phone or in-person interviews were conducted between February and October 2020. A hybrid technique of theory-driven and data-driven thematic analysis was applied.

**Results**: Emergent themes suggest that patients find rehabilitation more meaningful when it is "personally valued"; "self-defined"; and related to their "sense of self-identity". In addition, participants in this study were seeking a "genuine connection" with their therapist; and that the therapist should be "credible"; and a "guiding partner" in their chronic pain rehabilitation.

**Conclusion**: Emergent themes suggest that meaningfulness is an important construct in treatment adherence and outcomes for people with chronic pain. Contemporary practice encourages therapists to maintain professional boundaries, a tenet that requires more nuanced examination so it is not at odds with the "genuine connection" that participants described as a meaningful aspect of their rehabilitation. This study lays a foundation to better understand client perceptions of meaningfulness in rehabilitation and thus advance best-practice.

### ORCID

Katrina J. Liddiard http://orcid.org/0000-0003-3826-2631
Annette J. Raynor http://orcid.org/0000-0002-6517-3872
Helen M. DeJong http://orcid.org/0000-0002-7390-4110
Cary A. Brown http://orcid.org/0000-0002-5282-4170

© 2021 The Author(s). Published with license by Taylor & Francis Group, LLC.
This is an Open Access article distributed under the terms of the Creative Commons Attribution License (http://creativecommons.org/licenses/by/4.0/), which permits unrestricted use, distribution, and reproduction in any medium, provided the original work is properly cited.

### The Analgesic Effectiveness of Repeated Administration of Sucrose for Procedural Pain Relief in Hospitalized Preterm Infants

Mariana Bueno[a], Marilyn Ballantyne[b], Marsha Campbell-Yeo[c], Kimberley Dionne[d], Carole Estabrooks[e], Sharyn Gibbins[f], Denise Harrison[g], Carol McNair[h], Shirine Riahi[i], Souraya Sidani[j], Janet Squires[k], Anne Synnes[l], Anna Taddio[m], Charles Victor[n], Kimberley Widger[o], Janet Yamada[p], and Bonnie Stevens[q]

[a]The Hospital for Sick Children, Child Health Evaluative Sciences, Toronto, Ontario, Canada; [b] Holland Bloorview Kids Rehabilitation Hospital, and University of Toronto, Lawrence S. Bloomberg Faculty of Nursing, Toronto, Ontario, Canada; [c] Dalhousie University, School of Nursing, Faculty of Health, Departments of Pediatrics and Psychology & Neuroscience, and IWK Health Centre, Centre for Pediatric Pain Research, Halifax, Nova Scotia, Canada; [d] The Hospital for Sick Children, Toronto, Ontario, Canada; [e]University of Alberta, Edmonton Health Clinic Academy, Edmonton, Alberta; [f] Trillium Health Partners, Professional Practice, Mississauga, Ontario, Canada; [g] University of Melbourne, School of Health Sciences, Department of Nursing, Melbourne, Australia, and School of Nursing, Faculty of Health Sciences, University of Ottawa, Ottawa, Ontario, Canada; [h] The Hospital for Sick Children, Toronto, Ontario, Canada; [i]Shirine Riahi, RN MN, The Hospital for Sick Children, Child Health Evaluative Sciences, Toronto, Ontario, Canada; [j] Ryerson University, Daphne Cockwell School of Nursing, Toronto, Ontario, Canada; [k] School of Nursing, Faculty of Health Sciences, University of Ottawa, and Ottawa Hospital Research Institute, Ottawa, Ontario, Canada; [l] University of British Columbia, Pediatrics, Vancouver, British Columbia, Canada; [m]The Hospital for Sick Children, and University of Toronto, Leslie Dan Faculty of Pharmacy, Toronto, Ontario, Canada; [n]Strategic Partnerships & Digital Services, University of Toronto, Institute for Clinical Evaluative Sciences (ICES), The Institute of Health Policy,Management and Evaluation, Toronto, Ontario, Canada; [o] University of Toronto, Lawrence S. Bloomberg Faculty of Nursing, Toronto, Ontario, Canada; [p] Ryerson University, Daphne Cockwell School of Nursing, Toronto, Ontario, Canada; [q] University of Toronto, Lawrence S. Bloomberg Faculty of Nursing & Faculties of Medicine and Dentistry, and The Hospital for Sick Children, Child Health and Evaluative Sciences, Toronto, Ontario, Canada

**Introduction/Aim**: To determine the analgesic effectiveness of repeated sucrose administration for painful procedures in hospitalized preterm infants.
**Methods**: Prospective single group cohort conducted in four level III Canadian NICUs. Eligible infants were <32 weeks gestational age at birth, and <10 days of life at enrollment. From admission to discharge, infants received 24% sucrose (0.12ml) prior to all painful procedures that was repeated if the procedure lasted >1 minute and/or the infant showed signs of distress. No maximum sucrose dose limit was established. Pain intensity was measured using the Premature Infant Pain Profile-Revised (PIPP-R), at 30 and 60 seconds after a painful procedure. The first measurement occurred at enrolment and weekly for regularly scheduled blood work up to 4 measurements.
**Results**: 172 infants (57.3% male, mean GA at birth 28.35 (±2.31) weeks, mean birth weight 1,096.54 (±382.02) grams) underwent, on average, 91.5 (±141.66) painful procedures during hospitalization. The, mean 30 second PIPP-R scores were 6.11 (±3.68), 5.76 (±3.41), 6.48 (±3.67), and 6.81 (±3.69); there were no differences over time (p=0.15) nor study site by time (p=0.31). At 60 seconds, PIPP-R scores were 6.05 (±4.09), 5.74 (±3.67), 6.19 (±3.7), and 5.99 (±3.76); there were no differences over time (p=0.52) or site by time (p=0.14).
**Discussion/Conclusions**: Consistently low PIPP-R scores indicate infants experienced minimal pain across all timepoints. The analgesic effectiveness of sucrose appears to be sustained over time for procedural pain in preterm infants. Further studies on the effects of long-term use of sucrose for repeated painful procedures on development are required.

### ORCID

Mariana Bueno http://orcid.org/0000-0002-1470-1321
Marilyn Ballantyne http://orcid.org/0000-0003-4003-7126
Denise Harrison http://orcid.org/0000-0001-7549-7742
Bonnie Stevens http://orcid.org/0000-0001-5387-2302

© 2021 The Author(s). Published with license by Taylor & Francis Group, LLC.
This is an Open Access article distributed under the terms of the Creative Commons Attribution License (http://creativecommons.org/licenses/by/4.0/), which permits unrestricted use, distribution, and reproduction in any medium, provided the original work is properly cited.

### Access to chiropractic care during the COVID–19 lockdown in Spain influences pain and cognition: a pragmatic non-randomized controlled trial

Carlos Gevers-Montoro [a,b], Zoha Deldar [b], Francisco M. Conesa-Buendía [c], Eric A. Lazar [d], Ignacio Mahillo-Fernandez [e], Ali Khatibi [f], and Arantxa Ortega de Mues [g]

[a]RCU María Cristina, Chiropractic, San Lorenzo de El Escorial, Madrid, Spain; [b]Université du Québec à Trois-Rivières, Anatomy, Trois-Rivières, Québec, Canada; [c]Andalusian Network for Design and Translation of Advanced Therapies – Fundación Progreso y Salud, Cell Expansion and Therapy Unit, CTTC of Málaga, Málaga, Spain; [d]María Cristina, Chiropractic, San Lorenzo de El Escorial, Madrid, Spain; [e]IIS-Fundación Jiménez Díaz, Epidemiology and Biostatistics Unit, Madrid, Spain; [f]University of Birmingham, Centre of Precision Rehabilitation for Spinal Pain, Birmingham, United Kingdom; [g]RCU María Cristina, Chiropractic, San Lorenzo de El Escorial, Madrid, Spain

**Introduction/Aim**: Spain was one of the hardest-hit countries by the first wave of the COVID–19 pandemic. In March 2020, the Spanish government declared a state of emergency and the country went into full lockdown, restricting access to healthcare services such as chiropractic. Upon easing of the lockdown restrictions in May 2020, we aimed to examine the effects of access to chiropractic care on pain-related and psychological outcomes.

**Methods**: Upon reopening, 706 patients from 51 private chiropractic clinics throughout Spain were randomly invited to participate in this pragmatic controlled trial. Participants were exposed to hands-on chiropractic care (one or more encounters), or no care, for a 14-day period. Self-reported online outcomes were measured at baseline and follow-up after 14 days. Coprimary outcomes included pain intensity, interference, catastrophizing and kinesiophobia.

**Results**: Follow-up values were compared to baseline for 385 patients whose responses were deemed valid and paired (99 receiving no care; 177 one encounter; 109 multiple encounters). Pain intensity, interference and kinesiophobia significantly decreased, irrespective of the number of exposures. Baseline to follow-up evolution was significantly different for patients without access to care. Pain catastrophizing mediated the relationship between kinesiophobia, changes in pain intensity and pain interference.

**Discussion/Conclusions**: Exposure to hands-on chiropractic care during the COVID–19 pandemic may reduce pain, interference and fear of movement, partially by influencing pain catastrophizing. To help counterbalance the detrimental consequences of COVID–19 and lockdown on pain, physical activity and mental well-being, in-person care may be prioritized for patients with maladaptive pain cognitions.

### ORCID

Carlos Gevers-Montoro http://orcid.org/0000-0001-5031-8808
Zoha Deldar http://orcid.org/0000-0001-6532-8929
Francisco M. Conesa-Buendía http://orcid.org/0000-0001-8404-4382
Eric A. Lazar http://orcid.org/0000-0001-8863-3061
Ignacio Mahillo-Fernandez http://orcid.org/0000-0003-0558-6541
Ali Khatibi http://orcid.org/0000-0003-0679-0499
Arantxa Ortega de Mues http://orcid.org/0000-0002-4730-0129

© 2021 The Author(s). Published with license by Taylor & Francis Group, LLC.
This is an Open Access article distributed under the terms of the Creative Commons Attribution License (http://creativecommons.org/licenses/by/4.0/), which permits unrestricted use, distribution, and reproduction in any medium, provided the original work is properly cited.

### Circadian rhythmicity of chronic low back pain: a cross-sectional study

Mitra Knezic[a], Ana Constantin[a], Jesse Joynt[a], Elizabeth Brown[b], Daenis Camiré[c], Rosemary Wilson[d], Courtney Bannerman[a], Julia Segal[a], Cortney Haird[a], Tracey Stevenson[e], Chloé Nobis[a], Gabrielle Pagé[f], Scott Duggan[c], Ian Gilron [g], Etienne J. Bisson[c], and Nader Ghasemlou [c]

[a]Queen's University, Biomedical & Molecular Sciences, Kingston, Ontario, Canada; [b]Kingston Health Sciences Centre, Chronic Pain Clinic, Kingston, Ontario, Canada; [c]Queen's University, Anesthesiology & Perioperative Medicine, Kingston, Ontario, Canada; [d]Queen's University, School of Nursing, Kingston, Ontario, Canada; [e]Kingston Health Sciences Centre, Chronic Pain Clinic, Kingston, Ontario, Canada; [f]Research center of the Centre hospitalier de l'Université de Montréal (CRCHUM), Montréal, Québec, Canada; [g]Ian Gilron, Queen's University, Anesthesiology & Perioperative Medicine, Kingston, Ontario, Canada

**Introduction/Aim**: Understanding fluctuations in pain intensity is central to a thoughtful clinical assessment and may provide clues to the underlying causes. We sought to characterize the rhythmicity of pain and related symptoms in adults with chronic low back pain (CLBP) using ecological momentary assessment.

**Methods**: Adults with CLBP as their primary complaint were recruited from a tertiary chronic pain clinic prior to intake assessment. Following a baseline assessment, daily symptoms were tracked for 7 days with electronic diaries, where participants rated their pain, mood, and fatigue on a 0-10 numerical scale at 8:00AM, 2:00PM, and 8:00PM. Descriptive and comparative statistics were completed for several measures (e.g. pain intensity, depression, fatigue, pain catastrophizing, neuropathic pain qualities).

**Results**: While the majority of participants (n=63, 57% female, mean age=51± 14) displayed a constant pain profile, a subset (30%; nocturnal group) reported more intense pain at night relative to in the morning and a smaller group (13%; diurnal group) reported an inverse cyclical pattern (classified by percent change in pain score/day). Female participants with constant pain reported a significantly greater depressed mood (p=0.02) and fatigue (p=0.05) compared with their rhythmic counterparts. No correlation observed between neuropathic/inflammatory pain type and pain rhythmicity.

**Discussion/Conclusions**: We identified 3 differing patterns of daily pain intensity throughout the day in our population of adults with CLBP. As CLBP can arise from various inflammatory biological mechanisms which have been shown to have opposing rhythmic patterns, these findings may help influence future research and identify new therapeutic strategies to decrease or eliminate fluctuations in pain.

### ORCID

Ian Gilron http://orcid.org/0000-0002-5293-8792
Nader Ghasemlou http://orcid.org/0000-0002-1696-7342

© 2021 The Author(s). Published with license by Taylor & Francis Group, LLC.
This is an Open Access article distributed under the terms of the Creative Commons Attribution License (http://creativecommons.org/licenses/by/4.0/), which permits unrestricted use, distribution, and reproduction in any medium, provided the original work is properly cited.

### A critical scoping review of patient engagement in research of people with lived experience of poverty and opioid use

Bayan Alilyyani[a], Ryan Chan[b], Fiona Webster[c], and Laura Connoy[d]

[a]University of Western Ontario, Arthur and Sonia Labatt Family School of Nursing, London, Ontario, Canada; [b]University of Western Ontario, Arthur and Sonia Labatt Family School of Nursing, London, Ontario, Canada; [c]University of Western Ontario, Arthur and Sonia Labatt Family School of Nursing, London, Ontario, Canada; [d]University of Western Ontario, Arthur and Sonia Labatt Family School of Nursing, London, Ontario, Canada

**Introduction/Aim**: This scoping review represents one component of a multi-year research project on the experiences of people living with chronic pain who are also struggling with various forms of marginalization, including poverty, mental illness and addiction. The specific focus of this review is on patient engagement in relation to poverty and opioid use, with the aim of 'mapping' the literature in a way that brings coherence to this complex field. The guiding research question for this scoping review is: what frameworks and other tools have been developed in relation to engagement in research of of patients with chronic pain who are using opioids?

**Methods**: We followed the approach for scoping reviews proposed by Arksey and O'Malley, and Levac (2005). Key search terms were developed and databases screened, resulting in 483 articles. Following the removal of duplicates and application of inclusion/exclusion criteria, the final database was reduced to 34 articles. This process included a calibration exercise to ensure reliability. A data abstraction form was developed to compare all included articles. Data analysis involved quantitative (i.e. frequency analysis) and qualitative (i.e. thematic analysis) methods.

**Results**: The scoping review generated 5 themes : inadequate use of theory; poor conceptualization of patient engagement; a focus on opioids through a biomedical lens; negative and/or narrow conceptualizations of patients; and a focus that excluded social aspects of the pain experience.

**Discussion/Conclusions**: While the experiences of people with lived experience of chronic pain and marginalization are increasingly included in research studies, we found that there is still enormous room for improvement. We were limited by a narrow focus on one aspect of marginalization, poverty, and one aspect of pain management, opioid use. Nevertheless, we believe our findings have relevance more broadly to other research in the area of chronic pain.

© 2021 The Author(s). Published with license by Taylor & Francis Group, LLC.
This is an Open Access article distributed under the terms of the Creative Commons Attribution License (http://creativecommons.org/licenses/by/4.0/), which permits unrestricted use, distribution, and reproduction in any medium, provided the original work is properly cited.

### Inequitable outcomes: understanding chronic pain experiences among marginalized groups

Laura Connoy, Craig Dale, Joel Katz, Andrew Pinto, Kathleen Rice, Abhimanyu Sud, Ross Upshur, and Fiona Webster

University of Western Ontario, Arthur and Sonia Labatt Family School of Nursing, London, Ontario, Canada

**Introduction/Aim**: Our study focuses on the experiences of people living with chronic pain and social inequities (i.e. low socio-economic status, mental illnesses, and addictions) to better understand how they manage their chronic pain during the pandemic.

**Methods**: We use institutional ethnography (IE) to gain insights into how institutional priorities orient people's everyday experiences. It is important to note that IE refers to an approach to inquiry rather than a set of methods; the methods for this study are telephone interviews, given pandemic restrictions. Participants were asked to describe what they do as they go about their everyday lives.

**Results**: We highlight the following issues defining the lives of participants: affordability of prescribed medications and alternative therapies/medicines; food; housing; and general descriptions of struggle. For patients who have already been struggling with social isolation, the pandemic did not bring added burden but instead made them feel more connected to others who now "know what it's like". For others, the pandemic rules exacerbated mental health issues and further undermined access to social benefits and medical care.

**Discussion/Conclusions**: Our study highlights the interconnectivity between the social and the medical as they impact the lives of people with chronic pain. While studies are addressing how chronic pain is managed during the pandemic, they historically have not included those who are also marginalized by poverty, mental health issues or addictions. In highlighting these voices we draw attention to the importance of social and structural inequities that impact those living with chronic pain during COVID-19.

### ORCID

Craig Dale http://orcid.org/0000-0002-5042-0332
Joel Katz http://orcid.org/0000-0002-8686-447X
Abhimanyu Sud http://orcid.org/0000-0001-8354-6153

© 2021 The Author(s). Published with license by Taylor & Francis Group, LLC.
This is an Open Access article distributed under the terms of the Creative Commons Attribution License (http://creativecommons.org/licenses/by/4.0/), which permits unrestricted use, distribution, and reproduction in any medium, provided the original work is properly cited.

### How does Placebo and Nocebo Effects Modulate Pain in Children with ADHD: Preliminary Results

Carmen-Édith Belleï-Rodriguez[a], Serge Marchand[b], and Guillaume Léonard[a]

[a]University of Sherbrooke, Medicine and Health Science, Sherbrooke, Quebec, Canada; [b]Lucine, Sherbrooke, Quebec, Canada

**Introduction/Aim**: Attention deficit disorder with or without hyperactivity (ADHD) has significant negative consequences in the medical context and increases accidents and mortality risk[1]. Children with ADHD generally have higher pain levels and more widespread pain[2]. Children are highly influenced by the placebo/nocebo effect, an important factor influencing most treatments including pain[3,4]. The study aims to (1) compare the placebo/nocebo effect on pain between children with and without ADHD and to (2) characterize the association between placebo/nocebo effects and secondary variables (sleep, ADHD symptoms, anxiety and pain catastrophizing).

**Methods**: The data is collected from 44 children with/without ADHD. Placebo/nocebo effects are induced using inactive pills that "increase" (placebo) or "reduce" (nocebo) pain. Pain is induced using a thermode (thermal stimulation), and pain perception is continuously recorded using a computerized visual analogue scale.

**Results:** The preliminary results will be presented at the CPS congress. We anticipate that children with

ADHD should respond less to the placebo and more to the nocebo compared to children without ADHD. Independent samples t-tests will be used to compare placebo/nocebo effects between children with and without ADHD, and simple and multiple regression analysis to characterize the association between the placebo/nocebo effects on pain and the secondary variables.

**Conclusions**: This study will help to better understand the role of placebo/nocebo effects in children with and without ADHD and get a grasp on how children with ADHD can experience pain differently. These results will help to improve the intervention strategies to be implemented to guarantee better support for children.

### ORCID

Serge Marchand http://orcid.org/0000-0002-4234-5977
Guillaume Léonard http://orcid.org/0000-0002-3625-3072

### References

1. Dalsgaard, S., Østergaard, S. D., Leckman, J. F., Mortensen, P. B. & Pedersen, M. G. Mortality in children, adolescents, and adults with attention deficit hyperactivity disorder: a nationwide cohort study. *The Lancet* **385**, 2190–2196 (2015).
2. Stray, L. L. *et al.* Motor regulation problems and pain in adults diagnosed with ADHD. *Behav Brain Funct* **9**, 1–10 (2013).
3. Janiaud, P. *et al.* Is the perceived placebo effect comparable between adults and children? A meta-regression analysis. *Pediatr. Res.* **81**, 11–17 (2017).
4. Trouilloud, D. & Sarrazin, P. Note de synthèse: Les connaissances actuelles sur l'effet Pygmalion: processus, poids et modulateurs. *Revue française de pédagogie* **145**, 89–119 (2003).

© 2021 The Author(s). Published with license by Taylor & Francis Group, LLC.
This is an Open Access article distributed under the terms of the Creative Commons Attribution-NonCommercial License (http://creativecommons.org/licenses/by-nc/4.0/), which permits unrestricted non-commercial use, distribution, and reproduction in any medium, provided the original work is properly cited.

### Co-creating with TUNGSTEN: feasibility study to understand role of technology in chronic pain management

Shital Desai[a], Thusanthy Gunaseelan[b], Abhinayaa Jeyapragash[b], and Arlene Astell[c]

[a]York University, Social and Technological Systems lab, Toronto, Ontario, Canada; [b]University of Toronto, Occupational Science & Occupational Therapy, Toronto, Ontario, Canada; [c]KITE, University Health Network, Toronto, Ontario, Canada

**Introduction/Aim**: Technological advances have opened new avenues to support people with chronic pain (CP) and other stakeholders such as healthcare practitioners and caregivers. We conducted a feasibility study of the Tools for User Needs Gathering to Sustain Technology ENgagement (TUNGSTEN) as a co-creation approach to the role of technology in CP management.

**Methods**: Three people - one senior with CP and two occupational therapists working in CP services – participated in a virtual Show and Tell, a TUNGSTEN activity designed to understand what makes people love or abandon technology. Each was asked to show (if possible) and describe one loved and one abandoned technology. This was complemented with semi-structured interviews. The sessions were video recorded and thematically analysed in NVIVO.

**Results**: Data synthesis identified that technology is expected to be simple, versatile and include support for learning. Four facilitators of technology use for CP management were training, competency of the health care provider, support groups and peer mentoring and opportunity to trial it out. Barriers to technology use were transition care, learnability issues, lack of access to technology, perception of generational unfit and resulting aggravated pain. Additionally, expectations from healthcare providers included provision of visual instructions to use technology, recommendation of technologies that are appropriate to patients' environmental and personal circumstances and follow-up on the use of technology.

**Discussion/Conclusions**: This study confirmed the suitability of TUNGSTEN for co-creating technology in CP management and will inform future studies with larger sample size of people with CP and will include various types of CP such as arthritis. We have identified resources and platforms to use TUNGSTEN methods in virtual formats.

### ORCID

Shital Desai http://orcid.org/0000-0003-1851-0597
Arlene Astell http://orcid.org/0000-0002-6822-9472

© 2021 The Author(s). Published with license by Taylor & Francis Group, LLC.
This is an Open Access article distributed under the terms of the Creative Commons Attribution License (http://creativecommons.org/licenses/by/4.0/), which permits unrestricted use, distribution, and reproduction in any medium, provided the original work is properly cited.

### The clinical relevance of attentional biases in pediatric chronic pain: A longitudinal eye-tracking study

Sabine Soltani[a], Dimitri M. L. van Ryckeghem[b], Tine Vervoort[c], Lauren C. Heathcote[d], Keith Owen Yeates[e], Christopher Sears[f], and Melanie Noel[g]

[a]Department of Psychology, University of Calgary, Alberta, Canada; [b]Department of Experimental Clinical and Health Psychology, Ghent University; Department of Clinical Psychological Science, Maastricht University; Department of Behavioural and Cognitive Sciences, University of Luxembourg; [c]Department of Experimental Clinical and Health Psychology, Ghent, Belgium; [d]Department of Anesthesiology, Perioperative & Pain Medicine, Stanford Medicine, Stanford, California, United States; [e]Keith Owen Yeates, PhD, Department of Psychology, University of Calgary, Alberta, Canada; [f]Christopher Sears, PhD, Department of Psychology, University of Calgary, Alberta, Canada; [g]Melanie Noel, PhD, Department of Psychology, University of Calgary, Alberta, Canada

**Introduction/Aim**: Attentional biases have been posited as a key mechanism underlying the development and maintenance of chronic pain and co-occurring internalizing mental health symptoms. Despite theoretical prominence, a comprehensive understanding of the nature of biased attentional processing in chronic pain and its relationship to theorized antecedents and clinical outcomes is lacking, particularly in youth. The current study used eye-tracking technology to assess attentional biases for pain-related stimuli and their relationship to theorized antecedents of chronic pain and clinical outcomes.

**Methods**: Youth with chronic pain ($n$ = 125) and without chronic pain ($n$ = 52) viewed face images of varying levels of pain expressiveness while their eye gaze was tracked and recorded. At baseline, youth completed questionnaires to assess pain characteristics, theorized antecedents (pain catastrophizing, fear of pain, anxiety sensitivity), and clinical outcomes (pain intensity, interference, anxiety, depression, posttraumatic stress). For youth with chronic pain, outcomes were re-assessed at 3 months to assess for relationships with attentional bias while controlling for baseline symptoms.

**Results**: In both groups, youth exhibited an attentional bias for painful facial expressions, with group differences in patterns of attending. For youth with chronic pain, attentional bias was not significantly associated with theorized antecedents or clinical outcomes at baseline or 3- month follow-up.

**Discussion/Conclusions**: These results lend support to and also challenge conceptual models of chronic pain. Additional studies employing more comprehensive and contextual paradigms for the assessment of attentional bias are needed to clarify the ways in which such biases may manifest and relate to clinical outcomes.

### ORCID

Melanie Noel http://orcid.org/0000-0003-3752-8055

© 2021 The Author(s). Published with license by Taylor & Francis Group, LLC.
This is an Open Access article distributed under the terms of the Creative Commons Attribution License (http://creativecommons.org/licenses/by/4.0/), which permits unrestricted use, distribution, and reproduction in any medium, provided the original work is properly cited.

### "Maybe I Would've Been More Comfortable in my Ignorance?": Exploring Future Physical Therapist's Lived Experience of an Elective Pain Course

Zoe A. Leyland[a], David Walton[b], and Elizabeth Anne Kinsella[c]

[a]Western University, Health Rehabilitation Sciences, London, Ontario, Canada; [b]Western University, School of Physical Therapy, London, Ontario, Canada; [c]McGill University, Institute of Health Sciences Education, Montreal, QC

**Introduction/Aim**: The Master of Physical Therapy program at Western University (Canada), an elective course, Understanding Pain in Rehabilitation has been an option for physical therapy students. The primary intention of the course is to engage in critical thinking on their assumptions of pain. A secondary (curricular) intention is to facilitate student reflection on how they see themselves as 'providers of pain management', at this point, the students' experiences of learning in this way has not been rigorously explored.

**Methods**: This study followed an interpretivist research design, which aims to uncover the lived experiences of a person or several people of a particular phenomenon of interest. Single semi-structured in-depth 45–90-minute interviews of recent students were audio recorded and transcribed. In addition, the reflective diaries of students were included in the data set with students' permission.

**Results**: The analysis process followed van Manen's (2016) detailed approach to analysis by reading transcriptions line by line, coding, and identifying emergent themes in the data. Reviewing reflective diaries of the participants and notes from the primary researcher after each interview facilitated reflexivity about assumptions, emergent interpretations, and facilitated critical consideration of data.

**Discussion/Conclusions**: Pain science education provided to future novice physiotherapists was described as an important component of physiotherapy education by those that participated in the elective course. Reflective diaries not only provided a way to conceptualize new information learned from the course, but also served as a tool for participants to reflect on their personal and professional experiences and transformed understandings of pain.

© 2021 The Author(s). Published with license by Taylor & Francis Group, LLC.
This is an Open Access article distributed under the terms of the Creative Commons Attribution License (http://creativecommons.org/licenses/by/4.0/), which permits unrestricted use, distribution, and reproduction in any medium, provided the original work is properly cited.

### Effect of Gender on Chronic Pain Prevalence in a Large Population Cohort

Gianluca Guglietti[a], Christophe Tanguay Sabourin[b], Luda Diatchenko[a], and Etienne Vachon-Presseau[a]

[a]McGill University, Faculty of Dentistry, Montreal, QC, Canada; [b]McGill University, Integrated Program in Neuroscience, Montreal, QC, Canada

**Introduction/Aim**: Women are overrepresented in nearly all chronic pain conditions and exhibit worse symptoms and response to treatment, yet the cause of this discrepancy is poorly understood. One obstacle to understanding this phenomenon is discriminating the influence of biological sex from gender identity on chronic pain prevalence.

**Methods**: We conducted an observational study with data obtained from the UKBB where we have access to pain status, hormones, and psychosocial data across multiple visits for a diverse population of 500,000 individuals. Using gender related psychosocial variables in a logistic regression model predicting sex, we developed a weighted gender index (WGI) for each participant.

**Results**: As expected, biological sex was associated with the prevalence of chronic pain, with women having an odds ratio (OR) of 1.35 (95% CI 1.29-1.42). Chronic pain was also independently associated with the WGI even after controlling for age and sex as covariates in an ANCOVA model ($p<0.005$), suggesting that gender identity may have an impact beyond biological sex on the prevalence of chronic pain. Increased femininity as measured by our WGI was associated with increased odds of have chronic pain (OR 1.14, CI 1.12-1.17); an effect that was specific to women when stratifying by sex (OR 1.17, CI 1.13-1.22). Gender was also associated with development of chronic pain at follow up among women who were healthy at study entry (OR 1.10, CI 1.04-1.17).

**Discussion/Conclusions**: This study addresses a knowledge gap in the etiology of chronic pain and could lead toward new personalized sex-based treatment.

### ORCID

Luda Diatchenko http://orcid.org/0000-0002-1350-6727

© 2021 The Author(s). Published with license by Taylor & Francis Group, LLC.
This is an Open Access article distributed under the terms of the Creative Commons Attribution-NonCommercial License (http://creativecommons.org/licenses/by-nc/4.0/), which permits unrestricted non-commercial use, distribution, and reproduction in any medium, provided the original work is properly cited.

### Social Activity and School Withdrawal in Adolescents with Dysmenorrhea

Kayla M. Wall[a] and Michelle M. Gagnon[b]

[a]Kayla M. Wall, M.Sc., University of Saskatchewan, Psychology and Health Studies, Saskatoon, Saskatchewan, Canada; [b]Michelle M. Gagnon, Ph. D., University of Saskatchewan, Psychology and Health Studies, Saskatoon, Saskatchewan, Canada

**Introduction/Aim**: Dysmenorrhea is the most prevalent gynecological concern among adolescents (Bernardi et al., 2017). Previous research has demonstrated that dysmenorrhea interferes with school attendance and participation in social activities (Söderman et al., 2018). However, the relationship between dysmenorrhea symptom severity and school absenteeism and social activity withdrawal has not been examined. As such, we examined the impact of dysmenorrhea on school attendance and social activity in a sample of North American adolescents. We hypothesized that more severe dysmenorrhea symptoms would be associated with more school absenteeism and withdrawal from social activities.

**Methods**: We recruited adolescents aged 14 to 18 living in North America to complete an online survey.

Following receipt of parental consent and adolescent assent, adolescents completed measures of menstrual characteristics, menstrual symptom severity, social functioning, anxiety and depression.

**Results**: One hundred and thirteen adolescent girls completed the survey (*M*age = 15.98). There was a significant positive relationship between menstrual symptom severity and school absenteeism due to menstrual pain in the last three months, $r = .451$, $p < .0001$, and 42.6% of participants reported missing school because of menstrual pain within the past three months. There was also a significant positive relationship between menstrual symptom severity and limited participation in social activities in the past three months, $r = .544$, $p < .0001$, and menstrual pain limited participation in social activities for 76.2% of teens.

**Discussion/Conclusions**: Menstrual symptom severity is positively associated with school absenteeism and limited participation in social activities. Implications of dysmenorrhea on functioning are discussed.

### ORCID

Michelle M. Gagnon http://orcid.org/0000-0002-2400-1898

© 2021 The Author(s). Published with license by Taylor & Francis Group, LLC.
This is an Open Access article distributed under the terms of the Creative Commons Attribution-NonCommercial License (http://creativecommons.org/licenses/by-nc/4.0/), which permits unrestricted non-commercial use, distribution, and reproduction in any medium, provided the original work is properly cited.

### Analysis of single cell RNA expression of NMDA receptor subunits across the spinal cord dorsal horn

Newton Martin[a], Ariel Levine[b], and Michael Hildebrand[c]

[a]Carleton University, Neuroscience, Ottawa, Ontario, Canada; [b]NIH, National Institute of Neurological Disorders and Stroke (NINDS), Bethesda, Maryland, United States; [c]Carleton University, Neuroscience, Ottawa, Ontario, Canada

**Introduction/Aim**: Differential patterns of N-methyl-D-aspartate receptor (NMDAR) subtype expression in the spinal cord superficial dorsal horn (SDH) mediate neuronal excitability in both physiological and pathological pain processing. Immunohistochemical analyses reveal differences in NMDAR subunit expression across lamina I and II of the SDH, as well as asymmetrical expression within laminae. The integrated nature of excitatory and inhibitory SDH neurocircuitry necessitates more precise cellular and molecular investigations. It is, therefore, critical to explore NMDAR subunit expression profiles for distinct cellular subpopulations in the SDH of rodent and human spinal cord.

**Methods**: The initial dataset was sourced from a recent meta-analysis combining 6 single-cell RNA sequencing studies on mouse lumbar spinal cord. The mRNA expression levels of seven NMDAR subunits: GRIN1, GRIN2A–D, and GRIN3A–B, were investigated within SDH neurons as well as within excitatory or inhibitory subpopulations of SDH neurons. Using R programming, both the percent of cells expressing a gene and the average expression levels were averaged and compared between excitatory and inhibitory neuronal populations.

**Results**: Expression levels widely varied across excitatory and inhibitory neuronal populations. Surprisingly, GRIN3A, a regulator of NMDAR synaptic trafficking and function, is highly expressed, but enriched in excitatory SDH neurons. Conversely, both SDH neuronal populations highly express GRIN2B, which is a key molecular determinant of spinal pain processing.

**Discussion/Conclusions**: The unique expression profiles of GRIN3A and GRIN2B in the SDH could underlie molecular mechanisms of spinal pain processing, which diverge from canonical NMDAR regulation and function at most mature CNS synapses.

© 2021 The Author(s). Published with license by Taylor & Francis Group, LLC.
This is an Open Access article distributed under the terms of the Creative Commons Attribution License (http://creativecommons.org/licenses/by/4.0/), which permits unrestricted use, distribution, and reproduction in any medium, provided the original work is properly cited.

### Partnering with School Principals to Improve the Experience of School-Based Vaccinations

Anna Taddio[a], Victoria Gudzak[b], Marlene Jantzi-Bauman[c], Charlotte Logeman[d], Lucie Bucci[e], and Noni MacDonald[f]

[a]Leslie Dan Faculty of Pharmacy, University of Toronto, Toronto, Ontario, Canada; [b]Leslie Dan Faculty of Pharmacy, University of Toronto, Canada; [c]Wellington Dufferin Guelph Public Health, Guelph, Ontario, Canada; [d]Child Health Evaluative Sciences, SickKids, Toronto, Ontario, Canada; [e]Immunize Canada, Canadian Public Health Association, Ottawa, Canada; [f]Dalhousie University, Halifax, Nova Scotia, Canada

**Introduction/Aim**: While an efficient way to vaccinate large numbers of children, school vaccinations commonly cause pain and fear. In prior studies, public health staff have identified that school principals are important for trying to make vaccination clinics run smoothly. However, they do not systematically involve principals in planning vaccination clinics. The objective was to document school principal willingness to support various interventions to facilitate more positive vaccination experiences.
**Methods**: This study was part of a randomized cluster trial (n=40 schools) designed to evaluate the impact of a complex intervention (vs. control) on the vaccination experience. Intervention school principals met with school nurses to plan upcoming school vaccinations. Nurses inquired about the ability of principals to accommodate a variety of pain and fear mitigation strategies using a checklist.
**Results**: All 20 (100%) intervention school principals agreed to sending information to parents and staff ahead of time about pain and fear mitigation strategies. All agreed to provision of a main clinic space in a large and comfortable room (e.g., library) as well as a separate private clinic space, and 95% offered a separate clinic waiting area. All principals were agreeable to electronic devices as distraction agents; however, 40% limited them to school devices (rather than personal devices).
**Discussion/Conclusions**: Principals are agreeable to integration of pain and fear strategies in school vaccination clinics. Public health units are encouraged to partner with school principals to optimize vaccination clinic planning and delivery. This has the potential to improve the vaccination experience and support vaccination.

© 2021 The Author(s). Published with license by Taylor & Francis Group, LLC.
This is an Open Access article distributed under the terms of the Creative Commons Attribution License (http://creativecommons.org/licenses/by/4.0/), which permits unrestricted use, distribution, and reproduction in any medium, provided the original work is properly cited.

### Reduction of pain and spinal nociceptive transmission by working memory is load dependent

Zoha Deldar[a], Isabelle Blanchette[b], and Mathieu Piché[a]

[a]Université du Québec à Trois-Rivières, Department of Anatomy, Trois-Rivières, QC, Canada; [b]Université Laval, Department of Psychology, Québec, QC, Canada

**Introduction/Aim**: Cognitive-based approaches to pain regulation comprise attention and working memory (WM) engagement. Engaging WM leads to pain inhibition, but it is not clear if higher WM load enhances this effect. This study aimed at investigating: 1) the interaction between WM load and pain perception and 2) determining whether cerebrospinal mechanisms contribute to pain inhibition by WM, proportionally to WM load. We hypothesized that increasing the level of WM load decreases pain perception and nociception flexion reflex amplitude (NFR).

**Methods**: Thirty-eight healthy volunteers were randomly assigned to one of two n-back groups for which WM load was different (2-back or 3-back tasks). The experimental protocol comprised five counterbalanced conditions: 0-back, n-back (2-back or 3-back), pain, 0-back with pain, and n-back with pain. Pain and the NFR were evoked by transcutaneous electrical stimulation of the sural nerve.

**Results**: Pain was significantly different between conditions, but not between n-back groups. Both the 0-back and n-back tasks reduced pain compared with pain alone, but the n-back task produced stronger pain inhibition compared with the 0-back task. NFR amplitude was significantly different between conditions but not between n-back groups. NFR was inhibited by the 0-back and n-back tasks, with no difference between the two tasks.

**Discussion/Conclusions**: WM engagement can inhibit pain and increasing WM load enhances this effect, but to a certain point. Moreover, the NFR inhibition by WM suggests that inhibition of pain by WM depends, at least in part, on cerebrospinal mechanisms.

**ORCID**

Mathieu Piché http://orcid.org/0000-0003-4171-2226

© 2021 The Author(s). Published with license by Taylor & Francis Group, LLC.
This is an Open Access article distributed under the terms of the Creative Commons Attribution License (http://creativecommons.org/licenses/by/4.0/), which permits unrestricted use, distribution, and reproduction in any medium, provided the original work is properly cited.

### The influence of experimental low back pain on the central control of the back muscles

Antoine Rohel[a], Mikaël Desmons[a], Guillaume Léonard[b], and Hugo Massé-Alarie[a]

[a]Laval University, Rehabilitation, Quebec, Quebec, Canada; [b]Sherbrooke University, Rehabilitation, Sherbrooke, Quebec, Quebec

**Introduction/Aim**: Low back pain modifies spine motor control. These modifications might be explained by pain-driven plasticity in the central nervous system, notably in areas involved in the control of back muscles. Although pain modifies the function of the primary motor cortex, its influence on other motor systems also involved in the control of low back muscles has been barely studied in humans. The objective was to determine the impact of experimental low back pain on the different motor networks (intracortical, spinal) and descending tracts (corticospinal, vestibulospinal) involved in the control of the back muscles.

**Methods**: Twenty-two healthy subjects were recruited. Different neural networks were tested by evaluating the responses of lumbar erector spinae muscle to transcranial magnetic stimulation (TMS – corticospinal[single pulse TMS]/intracortical excitability[paired-pulse TMS]), electric vestibular stimulation (EVS-vestibulospinal excitability) and a stretch reflex (spinal excitability). These measurements were made before, during and after the induction of experimental low back pain (capsaicin and heat – Pain group – n=12) or during the application of heat alone (Control group - n=10).

**Results**: The mean pain intensity during measurement was 4.9 (1.6) (Pain Group). A reduction in the EVS-induced short-latency responses was present during and post-pain compared to pre-pain only for the Pain group. No other pain effect was observed.

**Discussion/Conclusions**: Our results suggest that an experimental low back pain modulates the excitability of the vestibulospinal tract by a modification of the short latency response during and after the pain. The pain does not seem to influence intracortical and corticospinal excitability.

© 2021 The Author(s). Published with license by Taylor & Francis Group, LLC.
This is an Open Access article distributed under the terms of the Creative Commons Attribution License (http://creativecommons.org/licenses/by/4.0/), which permits unrestricted use, distribution, and reproduction in any medium, provided the original work is properly cited.

### Preliminary validation of the Pain Resilience Scale for individuals with postsurgical pain

P. Maxwell Slepian[a], Hance Clarke[b], Aliza Weinrib[a], and Joel Katz[c]

[a]Toronto General Hospital, Department of Anesthesia and Pain Management, Toronto, Ontario, Canada; York University, Department of Psychology, Toronto, Ontario, Canada; [b]Toronto General Hospital, Department of Anesthesia and Pain Management, Toronto, Ontario, Canada; University of Toronto, Department of Anesthesiology and Pain Medicine, Toronto, Ontario, Canada; [c]York University, Department of Psychology, Toronto, Ontario, Canada; Toronto General Hospital, Department of Anesthesia and Pain Management, Toronto, Ontario, Canada; University of Toronto, Department of Anesthesiology and Pain Medicine, Toronto, Ontario, Canada

**Introduction/Aim**: The Pain Resilience Scale (PRS) is a 14-item measure that captures an individual's ability to maintain behavioral engagement and regulate emotions and cognitions despite pain. The PRS has been validated in experimental settings as well as among individuals with chronic pain, and has been demonstrated to be protective against negative pain-related outcomes (e.g. pain intensity, pain-related disability, mental health). The current study sought to examine the validity of the PRS among patients with postsurgical pain.

**Methods**: Patients from the Transitional Pain Service (TPS) at Toronto General Hospital completed the PRS and measures of pain intensity, pain interference, quality of life, pain catastrophizing, depressive symptoms and anxiety. Cross-sectional analyses were conducted using patient responses completed prior to their initial TPS

visit after surgery (n = 57) to examine internal consistency and the pattern of bivariate correlations between PRS and other study variables.

**Results**: The PRS (α=0.91) and its subscales, Behavioral Perseverance (α=0.89), and Cognitive/Affective Positivity (α=0.89) demonstrated good internal consistency. As expected, the PRS was negatively correlated with pain catastrophizing (r=-0.55, $p<0.001$), depressive symptoms (r=-0.43, $p<0.001$), anxiety (r=-0.46, $p<0.001$), pain interference (r=-0.47, $p<0.001$), current pain intensity (r=-0.3, $p=0.01$), and positively associated with quality of life (r=0.36, $p<0.01$).

**Discussion/Conclusions**: These initial results are promising evidence of the validity of the PRS in postsurgical pain. Future research will examine further indices of reliability as well as predictive validity of the PRS in this population.

### ORCID

Hance Clarke http://orcid.org/0000-0003-4975-3823
Joel Katz http://orcid.org/0000-0002-8686-447X

© 2021 The Author(s). Published with license by Taylor & Francis Group, LLC.
This is an Open Access article distributed under the terms of the Creative Commons Attribution License (http://creativecommons.org/licenses/by/4.0/), which permits unrestricted use, distribution, and reproduction in any medium, provided the original work is properly cited.

### A Retrospective Study of Longitudinal Effectiveness of Repetitive Intravenous Lidocaine for Neuropathic Pain

Igor Wilderman[a] and Olga Pugacheva[a,b]

[a]Wilderman Medical Clinic; [b]Biological Sciences, Canadian Centre for Clinical Trials

**Introduction/Aim**: Intravenous lidocaine has peripheral and central analgesic and anti-inflammatory effect and has been reported as a safe and effective treatment in patients with neuropathic pain, including fibromyalgia. The aim of this retrospective study was to determine if repetitive intravenous lidocaine infusions have a cumulative or decremental effect on alleviating neuropathic pain.

**Methods**: Patients diagnosed with neuropathic pain were included in the analysis if they received at least four intravenous lidocaine infusions every two months on average and had follow up data recorded in their charts. Forty-two patients, 37 of whom were diagnosed with fibromyalgia, were eligible for the analysis.

Short-term lidocaine analgesia was evaluated by the reduction in NRS pain score (%). Long-term analgesia was evaluated at follow-up visit by the patient subjective impression of change in pain intensity (%) and duration of pain relief (days).

Statistical analysis was done using one-way repeated measure analysis of variance (ANOVA).

**Results**: The results indicated that there was no significant effect of number of previous lidocaine treatments on short-term or long-term analgesia (p-values = 0.200 - 0.989).

**Discussion/Conclusions:** Repetitive IV lidocaine for patients with chronic neuropathic pain don't have either cumulative, or decremental effect on pain relief. Other factors can influence the patient individual perception of pain and treatment outcome. They include worsening of co-morbid conditions, change in medications, stress, and mood disorders. For the clinical practice, our results can be interpreted as favourable for the use of repetitive lidocaine infusions for the patients who initially benefited from them.

© 2021 Wilderman Medical Clinic. Published with license by Taylor & Francis Group, LLC.
This is an Open Access article distributed under the terms of the Creative Commons Attribution License (http://creativecommons.org/licenses/by/4.0/), which permits unrestricted use, distribution, and reproduction in any medium, provided the original work is properly cited.

### A Qualitative Study to Explore Patient Perspectives of Prophylactic Treatment with Botox for Chronic Migraine

Igor Wilderman MD, Deborah Tallarigo PhD, and Olga Pugacheva PhD

Wilderman Medical Clinic, Thornhill, Ontario, Canada

**Introduction/Aim**: Currently, onabotulinumtoxinA (Botox) is the most used prophylactic treatment for chronic migraine. Evidence from large-scale clinical trials and more recent real-world clinical studies confirm that Botox can be an effective treatment to manage chronic migraine. The objectives of this study were to explore patient experience and perception of prophylactic treatment with Botox for chronic migraine.

**Methods**: Data were collected using semi-structured interviews using open-ended questions, to uncover rich descriptive data on patient experiences. Interviews were transcribed and analysed using NVivo data analysis software to code and identify themes across the dataset.

**Results**: For patients who received at least one Botox treatment, five main themes emerged, which described patients' expectations, experiences, and feelings towards their treatment decisions. Barriers to initiate or continue prophylactic treatment with Botox were also identified.

**Discussion/Conclusions**: Understanding the patients' perspective is an important part of clinical practice and may impact on decision-making. Although migraines were not eliminated in the majority of patients in this study, patients viewed their treatment as effective, supporting the need for qualitative research studies to accompany clinical efficacy studies, since patients may place more importance on factors other than clinical parameters that are measured in clinical trials. These qualitative data can provide a more holistic perspective of patient care when treating chronic migraine and insights that may not be evident from the results of a clinical trial. This study also revealed potential barriers to treatment that can inform future policy and practice.

© 2021 Wilderman Medical Clinic. Published with license by Taylor & Francis Group, LLC.
This is an Open Access article distributed under the terms of the Creative Commons Attribution License (http://creativecommons.org/licenses/by/4.0/), which permits unrestricted use, distribution, and reproduction in any medium, provided the original work is properly cited.

### Pain at Rest, Movement Evoked Pain, and their Ratio Predict Pain and Disability at Six- and 12-Months after Distal Radius Fracture

Maryam Farzad[a], Joy Macdermid[b], Saurabh Mehta[c], Ruby Grewal[d], and Erfan Shafiee[e]

[a]School of Physical Therapy, Department of Health and Rehabilitation Sciences, University of Western Ontario. Roth McFarlane Hand and Upper Limb Centre, St. Joseph's Hospital, London, Ontario, Canada; [b]Physical Therapy and Surgery, Western University, London, ON and Co-director Clinical Research Lab, Hand and Upper Limb Centre, St. Joseph's Health Centre, London, Ontario; Professor Rehabilitation Science McMaster University, Hamilton, ON, Canada; [c]School of Physical Therapy, Marshall University, Huntington, WV, USA and Clinical Associate Professor, Department of Orthopedic Surgery, Joan C Edwards School of Medicine, Marshall University, Huntington, WV, USA; [d]Roth McFarlane Hand and Upper Limb Center, Department of Surgery, Western University, London, ON, Canada; [e]School of Physical Therapy, Department of Health and Rehabilitation Sciences, University of Western Ontario. London, Ontario, Canada

**Introduction/Aim:** To examine the associations of indicators of pain such as pain at rest (PAR), movement evoked pain (MEP), and the ratio between those (MEPR) assessed at 8-weeks following distal radius fracture (DRF) with occurrence of chronic pain or disability at 6- and 12-months after the injury. The study also examined whether these pain indicators can classify individuals who do or do not transition to chronic pain and disability.

**Method**: Scores for the pain and function subscales of patient-rated wrist evaluation (PRWE) were extracted for 8-weeks, 6- and 12-months after DRF from an existing dataset. The associations between pain indicators with chronic pain and disability were examined using nonlinear quartile and logistic regressions. The area under the curve (AUC) obtained to examine the accuracy of the predictors in classifying those who experienced chronic pain and disability after DRF.

**Results**: A total of 229 participants with completed data were included in the study. The PAR ≥3 (AUC of 0.77), and MEP≥6 (AUC of 0.78) predicted moderate to severe pain at 6-months after DRF. MEP of ≥7 predicted disability (AUC of 0.79) at 6-months. A score of 2≤or ≥8 of MEPR was associated with adverse outcomes at 6- and 12-months, but prediction accuracy was low (AUC≤ 0.50).

**Conclusion**: Pain at rest was more predictive of chronic pain, while pain with movement was more predictive of disability. The scores for PAR ≥3, and MEP ≥7 at 8-weeks should serve as a red flag for risk of chronic pain and disability after DRF.

© 2021 The Author(s). Published with license by Taylor & Francis Group, LLC.
This is an Open Access article distributed under the terms of the Creative Commons Attribution License (http://creativecommons.org/licenses/by/4.0/), which permits unrestricted use, distribution, and reproduction in any medium, provided the original work is properly cited.

### A Scoping Review of the Evidence Regarding Assessment and Management of Psychological Features of Shoulder Pain

Maryam Farzad[a,b], Joy MacDermid[c], David Ring[d], and Erfan Shafiee[a,e]

[a]School of Physical Therapy, Department of Health and Rehabilitation Sciences, University of Western Ontario; [b]Roth McFarlane Hand and Upper Limb Centre, St. Joseph's Hospital, London, Ontario, Canada; [c]Physical Therapy and Surgery, Western University, London, ON and Co-director Clinical Research Lab, Hand and Upper Limb Centre, St. Joseph's Health Centre, London, Ontario; Rehabilitation Science McMaster University, Hamilton, ON, Canada; [d]Dell Medical School. The University of Texas at Austin, Texas, USA; [e]Occupational Therapy, University of Social Welfare and Rehabilitation Sciences, Tehran, Iran

**Background and aim**: Psychological factors, social factors and mental health can contribute to musculoskeletal pain. Consideration of these factors, in combination with physical factors, is needed to optimize management. Shoulder pain is second only to low back pain among costs associated with the care of musculoskeletal disorders. The purpose of this scoping review was to identify the nature of the research that has integrated psychological assessment and treatment in the management of shoulder pain.

**Methods:** A scoping review of research studies identified through Pubmed, EMBASE, and CINAHL and graduate theses identified using Google scholar was conducted to determine studies and systematic reviews that assess and manage psychological aspects of shoulder, neck and upper back pain in adults. The search terms were psychological factors, anxiety, depression, catastrophic thinking, fear of movement and cognitive behavioural treatment. Two investigators screened study titles and abstracts. Data extraction, content analysis and thematic coding focused on the dimensions of pain addressed (emotional, behavioural, cognitive) and treatment approaches used (dimensions targeted, specific treatment parameters).

**Results:** Ten studies (7 randomized trials, three cohorts) addressed the management of the psychological aspects of shoulder pain. Out of seven RCTs, four compared psychological interventions with usual cares. Eight studies used cognitive approaches and three used behavioural approaches. Pain intensity was addressed as their primary outcome in two studies and as secondary outcome in five studies. Cognitive factors were evaluated in 60% of articles using ten measures. Emotional factors were evaluated in 70% articles using seven measures. Reduction of pain intensity and catastrophic thinking with respect to pain was achieved in most of the studies (70%).

**Conclusions:** A small pool of studies evaluated measurement and treatment of mental health and the effect on symptom intensity and psychological outcomes, both of which generally improved compared to standard care. Cognitive behavioural therapy may have a broad benefit as this review indicted positive effects on a larger array of outcomes than other psychological interventions. Limited description and classification of treatment components hinders review and implementation. In-depth study of the importance and methods of psychological treatment approaches in pain reduction is merited.

© 2021 The Author(s). Published with license by Taylor & Francis Group, LLC.
This is an Open Access article distributed under the terms of the Creative Commons Attribution License (http://creativecommons.org/licenses/by/4.0/), which permits unrestricted use, distribution, and reproduction in any medium, provided the original work is properly cited.

### Inequitable outcomes: understanding chronic pain experiences among marginalized groups

Laura Connoy, Craig Dale, Joel Katz, Andrew Pinto, Kathleen Rice, Abhimanyu Sud, Ross Upshur, and Fiona Webster

**Introduction/Aim**: Our study focuses on the experiences of people living with chronic pain and social inequities (i.e. low socio-economic status, mental illnesses, and addictions) to better understand how they manage their chronic pain during the pandemic.

**Methods**: We use institutional ethnography (IE) to gain insights into how institutional priorities orient people's everyday experiences. It is important to note that IE refers to an approach to inquiry rather than a set of methods; the methods for this study are telephone interviews, given pandemic restrictions. Participants were asked to describe what they do as they go about their everyday lives.

**Results**: We highlight the following issues defining the lives of participants: affordability of prescribed medications and alternative therapies/medicines; food; housing; and general descriptions of struggle. For patients who have already been struggling with social isolation, the pandemic did not bring added burden but instead made them feel more connected to others who now "know what it's like". For others, the pandemic rules exacerbated mental health issues and further undermined access to social benefits and medical care.

**Discussion/Conclusions**: Our study highlights the interconnectivity between the social and the medical as they impact the lives of people with chronic pain. While studies are addressing how chronic pain is managed during the pandemic, they historically have not included those who are also marginalized by poverty, mental health issues or addictions. In highlighting these voices we draw attention to the importance of social and structural inequities that impact those living with chronic pain during COVID-19.

### ORCID

Craig Dale http://orcid.org/0000-0002-5042-0332
Joel Katz http://orcid.org/0000-0002-8686-447X
Abhimanyu Sud http://orcid.org/0000-0001-8354-6153

© 2021 The Author(s). Published with license by Taylor & Francis Group, LLC.
This is an Open Access article distributed under the terms of the Creative Commons Attribution License (http://creativecommons.org/licenses/by/4.0/), which permits unrestricted use, distribution, and reproduction in any medium, provided the original work is properly cited.
